# Complex fermatean fuzzy *N*-soft sets: a new hybrid model with applications

**DOI:** 10.1007/s12652-021-03629-4

**Published:** 2022-02-04

**Authors:** Muhammad Akram, Umaira Amjad, José Carlos R. Alcantud, Gustavo Santos-García

**Affiliations:** 1 Department of Mathematics, University of the Punjab, New Campus, Lahore, Pakistan; 2 BORDA Research Unit and IME, University of Salamanca, 37007 Salamanca, Spain; 3 IME, University of Salamanca, 37007 Salamanca, Spain

**Keywords:** Complex Fermatean fuzzy set, *N*-soft set, Decision making

## Abstract

Decision-making methods play an important role in the real-life of human beings and consist of choosing the best options from a set of possible choices. This paper proposes the notion of complex Fermatean fuzzy *N*-soft set ($\hbox {CFFNS}_f$S) which, by means of ranking parameters, is capable of handling two-dimensional information related to the degree of satisfaction and dissatisfaction implicit in the nature of human decisions. We define the fundamental set-theoretic operations of $\hbox {CFFNS}_f$S and elaborate the $\hbox {CFFS}_f$S associated with threshold. The algebraic and Yager operations on $\hbox {CFFNS}_f$ numbers are also defined. Several algorithms are proposed to demonstrate the applicability of $\hbox {CFFNS}_f$S to multi-attribute decision making. The advanced algorithms are described and accomplished by several numerical examples. Then, a comparative study manifests the validity, feasibility, and reliability of the proposed model. This method is compared with the Fermatean fuzzy Yager weighted geometric ($\hbox {FFY}_w$G) and the Fermatean fuzzy Yager weighted average ($\hbox {FFY}_w$A) operators. Further, we developed a remarkable $\hbox {CFFNS}_f$-TOPSIS approach by applying innovative $\hbox {CFFNS}_f$ weighted average operator and distance measure. The presented technique is fantastically designed for the classification of the most favorable alternative by examining the closeness of all available choices from particular ideal solutions. Afterward, we demonstrate the amenability of the initiated approach by analyzing its tremendous potential to select the best city in the USA for farming. An integrated comparative analysis with existing Fermatean fuzzy TOPSIS technique is rendered to certify the terrific capability of the established approach. Further, we decisively investigate the rationality and reliability of the presented $\hbox {CFFNS}_f$S and $\hbox {CFFNS}_f$-TOPSIS approach by highlighting its advantages over the existent models and TOPSIS approaches. Finally, we holistically describe the conclusion of the whole work.

## Introduction

Multi-attribute decision-making (MADM) methods play an important role in the real life of human beings. The process of choosing the best option among a set of possible options is present in all human activities. Decision making in the domain of crisp sets to handle exact and precise data has been a growing field of research for mathematicians.

### Related work

Given the dubious and erroneous nature of human decisions, the limitations of decision making in the area of crisp set have gained importance over time. Zadeh (1965) was the pioneer who coped with the fuzziness and ambiguity of human decisions in the field of decision making. Fuzzy set (FS) theory refined not only the decision making, but also the related fields like social sciences, production management, etc. (Abdullah et al. 2012; Alcantud and Andrés Calle 2017; Guiffrida and Nagi 1998). FS theory empowers the experts to use their complacency level (membership/belongingness degree) with attributes whose values are between 0 and 1.

Undoubtedly, FS theory allows to work with unsettling analysis in the field of decision making. Nevertheless, FS theory could not assess the nature of satisfaction and dissatisfaction with human decisions. To salvage these shortcomings, Atanassov (1986) extended the FS with intuitionistic fuzzy sets (IFS) and added the non-membership function which is limited to the interval [0,1] in order to express the level of discontent with human decisions. In his model the sum of satisfaction and dissatisfaction degrees is in the unit interval.

In 2013, Yager (2013a, 2013b) adapted the conditions of IFS, to present the novel concept of Pythagorean fuzzy set (PFS) with relaxing conditions that the sum of square of belongingness degree and non-membership degree should enclose in unit interval. Due to the constraints in PFS, Yager (2016) introduced the model of q-rung orthopair fuzzy set (q-ROFS) with conditions that sum of $\hbox {q}^{th}$ power of belongingness degree and non-membership degree should not exceed from 1. Later on, Senapati and Yager (2020) developed the theory of Fermatean fuzzy set (FFS) that is more general model than IFS and PFS in which the cubic sum of membership degree and non-membership degree should lie in unit interval. FFS as an extension of IFS and PFS can support more amount of inexactness and vagueness that provide more precise results in decision making framework.

Aforementioned models were not applicable in 2-dimensional problems. Thus, Ramot et al. (2002) introduced the complex fuzzy set (CFS) which was proposed by the emerging relationship of complex and FS theory having complex unit circle as the range of membership function that enables the CFS to handle the 2-dimensional information along with amplitude and phase terms. The amplitude part and phase part both are real-valued functions which can take values from the unit interval to show the vagueness of both dimensions. Later, Alkouri and Salleh (2012) put forward the idea of complex intuitionistic fuzzy set (CIFS), in order to describe the non-membership degree along with membership degree within the complex unit circle, where the sum of phase terms and amplitude terms of belongingness degree and falseness degree should not exceed from 1. Further, Akram and Naz (2019) & Ullah et al. (2020) presented the new model of complex Pythagorean fuzzy set (CPFS), as an extension of CIFS, which has more generalized structure than CFS and CIFS as it possesses more relaxed conditions on the phase and amplitude terms.

The idea of soft sets ($\hbox {S}_f$Ss) theory was proposed by Molodtsov (1999), who also presented its relevancy and remarkable significance in the fields of operational research, probability theory, game theory and smoothness of functions (Molodtsov 1999, 2004). Alcantud and Santos-García (2017) proposed a totally revised approach for $\hbox {S}_f$S based decision-making issues under imperfect information. Many researchers brought up many models to enhance the literature of $\hbox {S}_f$S, inclusive of fuzzy $\hbox {S}_f$Ss ($\hbox {FS}_f$Ss) (Maji et al. 2001b), Intuitionistic $\hbox {FS}_f$Ss ($\hbox {IFS}_f$Ss) (Maji et al. 2001a), Pythagorean $\hbox {FS}_f$Ss ($\hbox {PFS}_f$Ss) (Peng et al. 2015), Fermatean $\hbox {FS}_f$Ss ($\hbox {FFS}_f$Ss) (Sivadas and John 2020), et cetera. The idea of a new perspective for the selection of best alternatives problems based on $\hbox {FS}_f$Ss was given by Alcantud (2016). Fatimah et al. (2019) worked on a new structure of $\hbox {S}_f$Ss, namely, probabilistic $\hbox {S}_f$S. Alcantud et al. (2017) proposed a new hybrid model named as valuation fuzzy $\hbox {S}_f$S and used it for real case study that uses data from the Spanish real estate market.

From latest studies of hybrid $\hbox {S}_f$S models, it can be concluded that primarily work of the researchers was based on real numbers between [0,1] or binary evaluation in $\hbox {S}_f$S models (Ma et al. 2017). But nowadays, objects are evaluated by non-binary structures such as voting system and rating or ranking objects. Due to that, numerous researchers for instance Alcantud and Laruelle (2014), Chen et al. (2013), and Herawan and Deris (2009) have worked in formal models for non-binary evaluations. Stimulated by these concerns, Fatimah et al. (2018) proposed the model of *N*-soft set ($\hbox {NS}_f$S) which is an extension of $\hbox {S}_f$S and encapsulate the idea of parameterized characterization of the alternatives that depend on the finite number of ordered grades. Fatimah and Alcantud (2021) introduced the idea of multi-fuzzy $\hbox {NS}_f$S. Later on, Akram et al. (2018, 2021b, 2021d) combined the concept of $\hbox {NS}_f$S with FS and explored the new hybrid model, namely, fuzzy *N*-soft set ($\hbox {FNS}_f$S). This novel concept involves the finite number of ordered grades along with the vagueness in the conception of the attributes that are used for decision making. Another hybrid model called the hesitant *N*-soft set was introduced by Akram et al. (2019a). Akram et al. (2019b) extended the idea of $\hbox {FNS}_f$S and presented the hybrid model of intuitionistic fuzzy *N*-soft set ($\hbox {IFNS}_f$S) that can also capture the non-membership grades. Moreover, Zhang et al. (1965) extended $\hbox {IFNS}_f$S to Pythagorean fuzzy *N*-soft set ($\hbox {PFNS}_f$S) that possesses more relaxed conditions than existing models. Recently, Akram and his contributors set forth the hybrid models of bipolar $\hbox {FNS}_f$Ss (Akram et al. 2021a), complex spherical $\hbox {FNS}_f$Ss (Akram et al. 2021c) and complex Pythagorean $\hbox {FNS}_f$Ss ($\hbox {CPFNS}_f$Ss) (Akram et al. 2021e).

The characteristic comparison of proposed and existing models is organized in Table 1 that present a broad view concerning the superiority of the manifested model.

**Table 1:** Characteristic comparison of the proposed and existent models

Models	Capable to address imprecise information	Capable to address 2-D information	Capable to address rating-based parameterized information	Have the features of generalization
FS (Zadeh 1965)	$\checkmark$	$\times$	$\times$	$\times$
$\hbox {NS}_f$S (Fatimah et al. 2018)	$\times$	$\times$	$\checkmark$	$\times$
$\hbox {FNS}_f$S (Akram et al. 2018)	$\checkmark$	$\times$	$\checkmark$	$\checkmark$
$\hbox {IFNS}_f$S (Akram et al. 2019b)	$\checkmark$	$\times$	$\checkmark$	$\checkmark$
$\hbox {PFNS}_f$S (Zhang et al. 1965)	$\checkmark$	$\times$	$\checkmark$	$\checkmark$
$\hbox {CPNS}_f$S (Akram et al. 2021e)	$\checkmark$	$\checkmark$	$\checkmark$	$\checkmark$
$\hbox {CFFNS}_f$S (proposed)	$\checkmark$	$\checkmark$	$\checkmark$	$\checkmark$

In recent years, a technique for order preference by similarity to the ideal solution (TOPSIS) was proposed by Hwang and Yoon (1981) to solve the MADM problems. The basic idea of TOPSIS technique is to find out the best opt which is closest to the positive ideal solution (PIS) and farthest away from the negative ideal solution (NIS). Chen (2000) utilized the TOPSIS technique for multi-attribute group decision-making (MAGDM) under a fuzzy environment. Li et al. (2019) applied the fuzzy TOPSIS approach for the case-study of the Beijing rail transportation system. Boran et al. (2009, 2011, 2012) built up the theory of intuitionistic fuzzy TOPSIS (IF-TOPSIS) and presented various real applications related to technology and business. Akram and his collaborators proposed the methodologies of interval-valued hesitant fuzzy TOPSIS (Akram and Adeel 2019), Pythagorean Fuzzy TOPSIS (PF-TOPSIS) (Akram et al. 2019c), and complex Pythagorean fuzzy TOPSIS (CPF-TOPSIS) (Akram et al. 2020) to address the tricky MAGDM problems. Senapati and Yager (2020) put forward the Fermatean fuzzy TOPSIS (FF-TOPSIS) to capture the MADM problems. Eraslan (2015) redesigned the TOPSIS approach under the environment of $\hbox {S}_f$S ($\hbox {S}_f$-TOPSIS) and illustrated the methodology by means of its potential application. Eraslan and Karaaslan (2015) adapted the approach of TOPSIS under the framework of $\hbox {FS}_f$Ss ($\hbox {FS}_f$-TOPSIS) and demonstrated its cogent applications to select the suitable house. Han et al. (2019) extended the technique of TOPSIS under entropy on $\hbox {PFS}_f$Ss environment and implemented it for the selection of missile position. Salsabeela and John presented the TOPSIS method based on $\hbox {FFS}_f$Ss ($\hbox {FFS}_f$-TOPSIS) (Salsabeela and John 2021) and elaborated it with the practical application for the selection of supplier for five-star hotel.

The comparison of proposed and existing techniques based on TOPSIS method, according to their characteristics, is arranged in Table 2 which provide an extensive view about the dominance of the presented methodology.

**Table 2:** Characteristic comparison of proposed and existing TOPSIS techniques

Approaches	Have capability to deal uncertain information	Have capability to deal 2-D information	Have capability to deal parameterized information	Have capability to deal rating-based parameterized information
Fuzzy-TOPSIS technique (Chen 2000)	$\checkmark$	$\times$	$\times$	$\times$
IF-TOPSIS technique (Boran et al. 2009)	$\checkmark$	$\times$	$\times$	$\times$
PF-TOPSIS technique (Akram et al. 2019c)	$\checkmark$	$\times$	$\times$	$\times$
FF-TOPSIS technique (Senapati and Yager 2020)	$\checkmark$	$\times$	$\times$	$\times$
CPF-TOPSIS technique (Akram et al. 2020)	$\checkmark$	$\checkmark$	$\times$	$\times$
$\hbox {S}_f$-TOPSIS technique (Eraslan 2015)	$\times$	$\times$	$\checkmark$	$\times$
$\hbox {FS}_f$-TOPSIS technique (Eraslan and Karaaslan 2015)	$\checkmark$	$\times$	$\checkmark$	$\times$
$\hbox {FFS}_f$-TOPSIS technique (Salsabeela and John 2021)	$\checkmark$	$\times$	$\checkmark$	$\times$
$\hbox {CFFNS}_f$-TOPSIS technique (proposed)	$\checkmark$	$\checkmark$	$\checkmark$	$\checkmark$

### Motivation

The motivation of the proposed hybrid model is given by the following facts:

- The idea of $\hbox {NS}_f$S captures the graded parameterized information but it has no potential to handle the fuzziness and vagueness of the provided data.
- The brilliant models of CIFS and CPFS are competitive frameworks for capturing the 2-dimensional vague data simultaneously. But they also have some restrictions due to the inadequacy of ranking based criteria.
- Moreover, the $\hbox {FFS}_f$S theory outstandingly renders the binary parameterized mechanism that handles ambiguity and vagueness of information with fantastic universality. But still, it is a 1-dimensional model that cannot present the uncertain periodic information as well as unable to cope with the ordered graded parameters of tricky practical problems.
- The decision-making technique based models $\hbox {FNS}_f$S, $\hbox {IFNS}_f$S and $\hbox {PFNS}_f$S can only deal with 1-dimensional data. None of the described models can handle 2-dimensional problems.
- Further, the novel idea of $\hbox {CPFNS}_f$S is an efficacious model with splendid characteristics to handle the obscurity of parameterized fuzzy information. Despite that, it has some flaws that spring up due to its restricted space.
- Classical TOPSIS technique is specifically devised to determine the optimal solution based on the assessed closeness of the preferences choices from the ideal solution. But this hypothetical technique must be altered to tackle the ordered graded obscurity and vagueness of inexact information.

Because of all these constraints motivated us to put forward the idea of a ground-breaking hybrid model called $\hbox {CFFNS}_f$Ss along with $\hbox {CFFNS}_f$-TOPSIS approach which competently handles two-dimensional information with relaxed conditions that cubic sum of amplitude and phase terms belongs to the interval [0,1]. Moreover, $\hbox {CFFNS}_f$S efficiently deals with the finite order grades of the alternatives according to the attributes. Therefore, the proposed model is the extension of $\hbox {FNS}_f$S (Akram et al. 2018), $\hbox {IFNS}_f$S (Akram et al. 2019b), $\hbox {PFNS}_f$S (Zhang et al. 1965), and $\hbox {CPFNS}_f$S (Akram et al. 2021e) models and in fact dominates overall traditional models of literature as it has comparatively wide range.

### Outline of the article

The essence of the first part of this article is to propose the hybrid model of $\hbox {CFFNS}_f$Ss and the related concepts including score function and accuracy function. Further, we investigate the remarkable properties and basic operations of $\hbox {CFFNS}_f$Ss. We have also constructed the $\hbox {CFFNS}_f$S derived by the threshold. Furthermore, algebraic and Yager operations for $\hbox {CFFNS}_f$ numbers ($\hbox {CFFNS}_f$Ns) are also defined. The proposed model is supported by the construction of three algorithms of decision-making and the applications are presented in contemplation of comparing the results of our algorithms. The comparative results of the model with existing $\hbox {FFY}_w$A (Garg et al. 2020) and $\hbox {FFY}_w$G (Garg et al. 2020) operators are given in the paper.

On the other hand, we revamp the TOPSIS approach for the environment of $\hbox {CFFNS}_f$ to account for MAGDM problems. The innovative $\hbox {CFFNS}_f$ weighted average operator and the distance measure of alternatives from positive and negative ideal solutions are employed to examine the contiguity of optimal variables from ideal solutions. The accountability of the presented technique is illustrated by implementing its magnificent procedure to select the suitable city in the USA for farming. A comparative analysis with the existing FF-TOPSIS (Senapati and Yager 2020) approach has been demonstrated to endorse the phenomenal feasibility and viability of the set forth strategy. The merits of the developed model and TOPSIS approach are also narrated for the appropriate manifestation of its marvelous and incredible feasibility over the existing models and approaches.

We summarize the main contributions of our research work as follows:

1. The article sets up a new theory of $\hbox {CFFNS}_f$S to deal with imprecise information involving vagueness and periodicity of ordered graded parameterized structure.
2. The algorithms are developed to tackle multi-attribute decision making problems by using numerical examples.
3. The comparative analysis with $\hbox {FFY}_w$A operator and $\hbox {FFY}_w$G operator to show the adequacy of the presented method.
4. This research also accomplishes a MAGDM technique, namely $\hbox {CFFNS}_f$-TOPSIS method.
5. The proposed technique is corroborated by a numerical example related to selecting the most suitable city in the USA for farming.
6. The $\hbox {CFFNS}_f$-TOPSIS approach is dexterously accomplished by demonstrating a comparative analysis with FF-TOPSIS method.

### Layout of the paper

From this point on, the paper is organized as follows. Section 2 contains some definitions of existing models. In Sect. 3, we introduce the novel concept of $\hbox {CFFNS}_f$S followed by operations on $\hbox {CFFNS}_f$Ss. Section 4 scrutinizes the algebraic and Yager operations on $\hbox {CFFNS}_f$Ns. Section 5 describes the three proposed algorithms of the decision-making process and also provides some applications of multi-variable decision-making procedures. Section 6 carries out a comparative analysis with existing models and offers experimental results that illustrate the effectiveness of the proposed algorithms. Then, Sect. 7 introduces the $\hbox {CFFNS}_f$-TOPSIS method for MAGDM problems. A real example and a comparative study of its usefulness is shown in Sects. 8 and 9 . Finally, merits of the proposed model and conclusions are drawn in Sects. 10 and 11 .

## Preliminaries

### Definition 2.1

(Molodtsov (1999)) Let *U* be a universe of discourse under consideration and ${\mathcal {A}}$ be the set of all attributes, ${\mathcal {B}} \subseteq {\mathcal {A}}.$ A pair $(\rho ,{\mathcal {B}})$ is called *soft set* over *U* if $\rho :{\mathcal {B}}\longrightarrow P(U)$ where $\rho$ is a set-valued function.

### Definition 2.2

(Fatimah et al. (2018)) Let *U* be a universe of discourse and ${\mathcal {A}}$ be the set of all attributes, ${\mathcal {B}}\subseteq {\mathcal {A}}.$ Consider ${\mathcal {R}}=\{0,1,\ldots ,N-1\}$ be a set of ordered grades where $N \in \{2,3,\ldots \}.$ A triple $({\mathcal {F}},{\mathcal {B}},N)$ is an $\hbox {NS}_f$*S* on *U* if ${\mathcal {F}}:{\mathcal {B}}\longrightarrow 2^{U \times {\mathcal {R}}},$ with the property that for each $b_t \in {\mathcal {B}}$ there exists a unique $(u_g,r_a)\in U \times {\mathcal {R}}$ such that $(u_g,r_a)\in {\mathcal {F}}(b_t),u_g \in U, r_{a_{gt}}\in {\mathcal {R}}.$

### Definition 2.3

(Senapati and Yager (2020)) Consider *U* be a universe of discourse. An FFS *E* on *U* is defined as an object of the form

$$\begin{aligned} {\mathfrak {F}}=(\varrho _{E},\varpi _{E})=\{(u_g,\varrho _{E}(u_g), \varpi _{E}(u_g)\mid u_g \in U\}, \end{aligned}$$

where the functions $\varrho _E:U\longrightarrow [0,1]$ and $\varpi _E:U\longrightarrow [0,1]$ denote the degree of membership (namely $\varrho _E(u_g)$) and the degree of non-membership (namely $\varpi _E(u_g)$) of the element $u_g \in U$, respectively, and for all $u_g \in U, 0 \le (\varrho _E(u_g))^3+(\varpi _E(u_g))^3 \le 1.$ The value $\chi _E(u_g) = \root 3 \of {1 - (\varrho _{E}(u_g))^3 -(\varpi _{E}(u_g))^3}$ is called degree of uncertainty of the elements $u_g \in U$ to the FFS *E*.

### Definition 2.4

A *complex Fermatean fuzzy set* (CFFS, in short) ${\mathcal {B}}$, defined on the universal set *U*,  is characterized by the membership and non-membership functions $\mu _{\mathcal {B}}(u_g)$ and $\nu _{\mathcal {B}}(u_g)$, respectively, which assign to each element $u_g\in U$ a complex-valued grade of membership and non-membership functions in ${\mathcal {B}}.$ The CFFS may be represented as the set of triples:

$$\begin{aligned} {\mathcal {B}} = \{\langle u_g, \mu _{\mathcal {B}} (u_g), \nu _{\mathcal {B}} (u_g) \rangle : u_g \in U\}, \end{aligned}$$

where $\mu _{\mathcal {B}}(u_g): U \longrightarrow \{ u_g\mid u_g \in \mathbb {C}, \mid u_g\mid \le 1\}$, $\nu _{\mathcal {B}}(u_g): U \longrightarrow \{ u_g'\mid u_g' \in \mathbb {C}, \mid u_g\mid \le 1\}$, such that $\mu _{\mathcal {B}}(u_g)= s_{{\mathcal {B}}}(u_g)e^{i\omega _{{\mathcal {B}}}(u_g)}, \nu _{\mathcal {B}}(u_g)= k_{{\mathcal {B}}}(u_g)e^{i\psi _{{\mathcal {B}}}(u_g)},$ where $i = \sqrt{-1}$ and $s_{{\mathcal {B}}}(u_g), k_{{\mathcal {B}}}(u_g),$ $\omega _{{\mathcal {B}}}(u_g), \psi _{{\mathcal {B}}}(u_g)$ are real-valued functions such that $s_{{\mathcal {B}}}(u_g), k_{{\mathcal {B}}}(u_g) \in [0,1],~ \omega _{{\mathcal {B}}}(u_g), \psi _{{\mathcal {B}}}(u_g) \in [0,2\pi ].$ $s_{{\mathcal {B}}}(u_g), k_{{\mathcal {B}}}(u_g)$ are called the amplitude terms and $\omega _{{\mathcal {B}}}(u_g), \psi _{{\mathcal {B}}}(u_g)$ are called the phase terms with $0\le (s_{\mathcal {B}}(u_g))^3+(k_{\mathcal {B}}(u_g))^3\le 1,$ and $0\le (\frac{\omega _{{\mathcal {B}}}(u_g)}{2\pi })^3+(\frac{\psi _{{\mathcal {B}}}(u_g)}{2\pi })^3\le 1.$

The term $\pi _{\mathcal {B}}(u_g)= j_{{\mathcal {B}}}(u_g)e^{i2\pi \vartheta _{{\mathcal {B}}}(u_g)}$ is called degree of indeterminacy, where $j_{\mathcal {B}}(u_g)=\root 3 \of {1-(s_{\mathcal {B}}(u_g))^3-(k_{\mathcal {B}}(u_g))^3}$ and $\vartheta _{{\mathcal {B}}}=\root 3 \of {1-(\frac{\omega _{{\mathcal {B}}}(u_g)}{2\pi })^3-(\frac{\psi _{{\mathcal {B}}}(u_g)}{2\pi })^3}.$

### Definition 2.5

Let *U* be a universal set and ${\mathcal {A}}$ be the set of all attributes under consideration, ${\mathcal {B}} \subseteq {\mathcal {A}}.$ Let ${\mathcal {P}}(U)$ denotes the set of all complex Fermatean fuzzy (CFF) subsets of *U*. A pair $({\mathcal {P}},{\mathcal {B}})$ is called a $\hbox {CFFS}_f$*S* over *U*, where ${\mathcal {P}}$ is a function given by ${\mathcal {P}}:{\mathcal {B}}\longrightarrow {\mathcal {P}}(U),$ which is

$$\begin{aligned} {\mathcal {P}}_{e_{\mathcal {P}}}(b_t)=\{\langle u_g,s_{\mathcal {P}}(u_g)e^{i\omega _{{\mathcal {P}}}(u_g)}, k_{\mathcal {P}}e^{i\psi _{{\mathcal {P}}}(u_g)}\rangle ~|~ u_g\in U, b_t \in {\mathcal {B}}\}. \end{aligned}$$

## Complex Fermatean fuzzy *N*-soft sets

### Definition 3.1

Let *U* be a universal set and ${\mathcal {A}}$ be the set of all parameters under consideration, ${\mathcal {B}} \subseteq {\mathcal {A}}.$ Let ${\mathcal {R}}=\{0,1,2,\ldots ,N-1\}$ be a set of ordered grades where $N \in \{2,3,\ldots \}.$ A triple $({\mathcal {H}}, {\mathcal {Q}}, N)$ is called a *CFF N-soft set* (for short, $\hbox {CFFNS}_f$S), when ${\mathcal {Q}}=({\mathcal {F}},{\mathcal {B}},N)$ is an $\hbox {NS}_f$S on *U* and ${\mathcal {H}}$ is a mapping ${\mathcal {H}}:{\mathcal {B}}\longrightarrow CFF^{(U\times {\mathcal {R}})},$ where $CFF^{(U\times {\mathcal {R}})}$ is the collection of all *CFFSs* over $U\times {\mathcal {R}},$ which is

$$\begin{aligned} {\mathcal {H}}(b_t)=\{\langle (u_g,r_{a_{gt}}),s(u_g,r_{a_{gt}})e^{i\omega (u_g,r_{a_{gt}})}, k(u_g,r_{a_{gt}})e^{i\psi (u_g,r_{a_{gt}})}\rangle ~|~ b_t \in {\mathcal {B}}, (u_g,r_{a_{gt}})\in U\times {\mathcal {R}}\}. \end{aligned}$$

For convenience, ${\mathcal {H}}(b_t)=\langle (u_g,r_{a_{gt}}),s(u_g,r_{a_{gt}})e^{i\omega (u_g,r_{a_{gt}})}, k(u_g,r_{a_{gt}})e^{i\psi (u_g,r_{a_{gt}})}\rangle$ is denoted by $\alpha _{a_{gt}}=\langle r_{a_{gt}},(s_{a_{gt}}e^{i\omega _{a_{gt}}},k_{a_{gt}}e^{i\psi _{a_{gt}}}) \rangle$ which represents $\hbox {CFFNS}_f$ number ($\hbox {CFFNS}_f$N).

### Definition 3.2

Let $\alpha _{a_{gt}}=\langle r_{a_{gt}},(s_{a_{gt}}e^{i\omega _{a_{gt}}},k_{a_{gt}}e^{i\psi _{a_{gt}}}) \rangle$ be a $\hbox {CFFNS}_f$N then

$$\begin{aligned} \varLambda _{\alpha _{a_{gt}}}= \root 3 \of {1-((s_{a_{gt}})^3+(k_{a_{gt}})^3)}~e^{i 2\pi \root 3 \of {1-((\frac{\omega _{a_{gt}}}{2\pi })^3+(\frac{\psi _{a_{gt}}}{2\pi })^3)}} \end{aligned}$$

is called the degree of hesitancy/indeterminacy of $\hbox {CFFNS}_f$N.

### Definition 3.3

Let $\alpha _{a_{gt}}=\langle r_{a_{gt}},(s_{a_{gt}}e^{i\omega _{a_{{gt}}}},k_{a_{gt}},e^{i \psi _{a_{gt}}})\rangle$ be any $\hbox {CFFNS}_f$N over *U*. The score function and accuracy function of $\alpha _{a_{gt}}$ are defined as follows:

$$\begin{aligned} S(\alpha _{a_{gt}})= & {} (\frac{r_{a_{gt}}}{N-1})^3 +(s_{a_{gt}})^3 -(k_{a_{gt}})^3+\big ((\frac{\omega _{a_{gt}}}{2\pi })^3 -(\frac{\psi _{a_{gt}}}{2\pi })^3\big ), \\ A(\alpha _{a_{gt}})= & {} (\frac{r_{a_{gt}}}{N-1})^3+(s_{a_{gt}})^3+(k_{a_{gt}})^3 +\big ((\frac{\omega _{a_{gt}}}{2\pi })^3+(\frac{\psi _{a_{gt}}}{2\pi })^3\big ), \end{aligned}$$

respectively, where $S(\alpha _{a_{gt}}) \in [-2,3]$ and $A(\alpha _{a_{gt}}) \in [0,3].$

### Definition 3.4

For any two distinct $\hbox {CFFNS}_f$Ns $\alpha _{a_{gt}}$ and $\beta _{a_{lt}},$ we have:

1. if $$S(\alpha _{a_{gt}}) < S(\beta _{a_{lt}}),$$ then $$\alpha _{a_{gt}} < \beta _{a_{lt}},$$
2. if $$S(\alpha _{a_{gt}}) > S(\beta _{a_{lt}}),$$ then $$\alpha _{a_{gt}} > \beta _{a_{lt}},$$
3. if $$S(\alpha _{a_{gt}}) = S(\beta _{a_{lt}}),$$ then
  - if $$A(\alpha _{a_{gt}}) > A(\beta _{a_{lt}}),$$ then $$\alpha _{a_{gt}} > \beta _{a_{lt}},$$
  - if $$A(\alpha _{a_{gt}}) < A(\beta _{a_{lt}}),$$ then $$\alpha _{a_{gt}} < \beta _{a_{lt}},$$
  - if $$A(\alpha _{a_{gt}}) = A(\beta _{a_{lt}}),$$ then $$\alpha _{a_{gt}} = \beta _{a_{lt}}.$$

For a better understanding of the concept of our new model, we present the following example:

### Example 3.1

Consider that an auto broker decides to purchase the car from auto company. The best car is chosen by spade ratings endowed by an expert. These rankings are on the basis of launched cars in the last 5 years and their performances. Before purchasing the car, auto broker obtained some rating and ranking based information from an expert about four different models of vehicles having different manufacturing dates. Let ${X}=\{x_1,x_2,x_3,x_4\}$ be the set of vehicles and ${\mathcal {B}}=\{b_1 = \mathrm{Reliability}, b_2 = \mathrm{Maximum~payload}, b_3 = \mathrm{Purchasing~cost} \}\subseteq {\mathcal {A}}$ be the set of attributes, that are used to set grades for each vehicle with respect to each attribute. The expert assigned the rating of the cars according to the above-mentioned conflicting criteria and the initial review recapped in Table 3, where:

- four spades represent ‘excellent’,
- three spades represent ‘very good’,
- two spades represent ‘good’,
- one spade represents ‘regular’, and
- a bullet represents ‘bad’.

The set of grades ${\mathcal {R}}=\{0,1,2,3,4\}$ can be easily associated with rated assessment conducted by bullet and spades as follows:

- 0 stands for $$`\bullet$$’,
- 1 stands for $$`\spadesuit$$’,
- 2 stands for $$`\spadesuit \spadesuit$$’,
- 3 stands for $$`\spadesuit \spadesuit \spadesuit$$’,
- 4 stands for $$`\spadesuit \spadesuit \spadesuit \spadesuit$$’.

Based on the overall qualities of the cars, the auto broker gives evaluation scores of the cars which is shown as Table 3 and the tabular representation of its associated 5-soft set is given in Table 4.

**Table 3:** Information extracted from the expert

$X/{\mathcal {B}}$	$b_{1}$	$b_{2}$	$b_{3}$
$x_{1}$	$\spadesuit \spadesuit$	$\spadesuit \spadesuit \spadesuit$	$\spadesuit$
$x_{2}$	$\spadesuit$	$\spadesuit \spadesuit \spadesuit$	$\spadesuit \spadesuit$
$x_{3}$	$\spadesuit \spadesuit \spadesuit$	$\spadesuit \spadesuit \spadesuit \spadesuit$	$\bullet$
$x_{4}$	$\bullet$	$\spadesuit \spadesuit$	$\spadesuit \spadesuit \spadesuit \spadesuit$

**Table 4:** Tabular form of the 5-soft set

$({\mathcal {F}}, {\mathcal {B}}, 5)$	$b_{1}$	$b_{2}$	$b_{3}$
$x_{1}$	2	3	1
$x_{2}$	1	3	2
$x_{3}$	3	4	0
$x_{4}$	0	2	4

The grade data in the actual information can be easily extracted. However, according to the Definition 3.1 when the data possess fuzzy uncertainty characteristics, we need $\hbox {CFFNS}_f$N. It provides us information in which the auto brokers evaluate the cars and specify their rankings based on the same multiple fuzzy characteristics from the perspective of the two-dimensional membership degree and non-membership degree. This assessment of cars by auto brokers complies with the guidelines as follows:

$$\begin{aligned} -2.0\le & S(X)< -1.2 \, \mathrm{when\, grade~} 0,\\ -1.2\le & S(X)< -0.4\, \mathrm{when \, grade~} 1,\\ -0.4\le & S(X)<~~ 0.4\, \mathrm{when \,grade~} 2, \\ 0.4\le & S(X)<~~1.2~\,~ \mathrm{when \,grade~} 3, \\ 1.2\le & S(X) <~~ 2.0~~\, \mathrm{when \,grade~} 4. \end{aligned}$$

According to above criteria, we can obtain Table 5.

**Table 5:** Grading criteria

$r_{a_{gt}}/{\mathcal {H}}$	Amplitude term		Phase term	
Grades	${s_{a_{gt}}}$	${k_{a_{gt}}}$	$\omega _{a_{gt}}$	$\psi _{a_{gt}}$
$r_{a_{gt}}=0$	[0, 0.2)	(0.85, 1]	$[0,0.4\pi )$	$(1.7\pi ,2\pi ]$
$r_{a_{gt}}=1$	[0.2, 0.4)	(0.65, 0.85]	$[0.4\pi ,0.8\pi )$	$(1.3\pi ,1.7\pi ]$
$r_{a_{gt}}=2$	[0.4, 0.65)	(0.4, 0.65]	$[0.8\pi ,1.3\pi )$	$(0.8\pi ,1.3\pi ]$
$r_{a_{gt}}=3$	[0.65, 0.85)	(0.2, 0.4]	$[1.3\pi ,1.7\pi )$	$(0.4\pi ,0.8\pi ]$
$r_{a_{gt}}=4$	[0.85, 1)	(0, 0.2]	$[1.7\pi ,2\pi )$	$(0,0.4\pi ]$

Therefore, by Definition 3.1, the CFF5$\hbox {S}_f$S $({\mathcal {H}},{\mathcal {Q}},5)$ can be defined as follows:

$$\begin{aligned} h(b_1)= & {} \{\langle (x_1,2),0.5e^{i0.9\pi },0.6e^{i1.2\pi }\rangle ,\langle (x_2,1), 0.3e^{i0.5\pi },0.7e^{i1.5\pi }\rangle ,\langle (x_3,3),0.7e^{i1.4\pi }, 0.3e^{i0.5\pi }\rangle ,\\&\langle (x_4,0),0.1e^{i0.2\pi },0.9e^{i1.8\pi }\rangle \}, \\ h(b_2)= & {} \{\langle (x_1,3),0.7e^{i1.5\pi },0.3e^{i0.5\pi }\rangle , \langle (x_2,3),0.8e^{i1.6\pi },0.4e^{i0.6\pi }\rangle ,\langle (x_3,4), 0.9e^{i1.8\pi },0.1e^{i0.3\pi }\rangle ,\\&\langle (x_4,2),0.5e^{i0.9\pi },0.5e^{i1.0\pi }\rangle \}, \\ h(b_3)= & {} \{\langle (x_1,1),0.3e^{i0.6\pi },0.8e^{i1.6\pi }\rangle , \langle (x_2,2),0.6e^{i0.9\pi },0.5e^{i1.2\pi }\rangle ,\langle (x_3,0), 0.1e^{i0.2\pi },0.9e^{i1.9\pi }\rangle ,\\&\langle (x_4,4),0.9e^{i1.9\pi },0.2e^{i0.3\pi }\rangle \}. \end{aligned}$$

The CFF5$\hbox {S}_f$S $({\mathcal {H}},{\mathcal {Q}},5)$ can be represented more clearly in tabular form shown as in Table 6 as follows:

**Table 6:** Tabular form of the CFF5$\hbox {S}_f$S $({\mathcal {H}}, {\mathcal {Q}}, 5)$

$({\mathcal {H}}, {\mathcal {Q}}, 5)$	$b_{1}$	$b_{2}$	$b_{3}$
$x_{1}$	$\langle 2,(0.5e^{i0.9\pi },0.6e^{i1.2\pi })\rangle$	$\langle 3,(0.7e^{i1.5\pi },0.3e^{i0.5\pi })\rangle$	$\langle 1,(0.3e^{i0.6\pi },0.8e^{i1.6\pi })\rangle$
$x_{2}$	$\langle 1,(0.3e^{i0.5\pi },0.7e^{i1.5\pi })\rangle$	$\langle 3,(0.8e^{i1.6\pi },0.4e^{i0.6\pi })\rangle$	$\langle 2,(0.6e^{i0.9\pi },0.5e^{i1.2\pi })\rangle$
$x_{3}$	$\langle 3,(0.7e^{i1.4\pi },0.3e^{i0.5\pi })\rangle$	$\langle 4,(0.9e^{i1.8\pi },0.1e^{i0.3\pi })\rangle$	$\langle 0,(0.1e^{i0.2\pi },0.9e^{i1.9\pi })\rangle$
$x_{4}$	$\langle 0,(0.1e^{i0.2\pi },0.9e^{i1.8\pi })\rangle$	$\langle 2,(0.5e^{i0.9\pi },0.5e^{i1.0\pi })\rangle$	$\langle 4,(0.9e^{i1.9\pi },0.2e^{i0.3\pi })\rangle$

### Remark 1

The following observations are in order:

1. In Example 3.1, we consider the five assessment grades, but the assessment grades in practical problems do not necessarily utilize the 5 grades, it can be arbitrary. Generally, the range concerning the score function of CFF numbers can vary with actual grade requirements.
2. Any CFF2$\hbox {S}_f$S $({\mathcal {H}},{\mathcal {Q}},2)$ can be naturally associated with a CFFS${_f}$S. We identify a CFF2$\hbox {S}_f$S ${\mathcal {H}}:{\mathcal {B}}\longrightarrow CFF^{(U\times \{0,1\})}$ with a CFFS${_f}$S $(\wp ,{\mathcal {B}}),$ which is given by:
  $$\begin{aligned} \wp (b_t)= \{\langle u_g,s_\wp (u_g)e^{i\omega _{\wp }(u_g)},k_{\wp }(u_g)e^{i\psi _{\wp }(u_g)} \rangle ~|~\langle (u_g,1),s_\wp (u_g)e^{i\omega _{\wp }(u_g)},k_{\wp }(u_g)e^{i\psi _{\wp }(u_g)} \rangle \in {\mathcal {H}}(b_t)\}, \end{aligned}$$
  for every $b_t\in {\mathcal {B}},$ where $CFF^{(U\times \{0,1\})}$ is the collection of all CFF subsets of $U\times \{0,1\}.$
3. An arbitrary $\hbox {CFFNS}_f$S over a universe *U* can be identified as a CFF $(N+1)$-soft set. For example, from Table 6, a CFF5$\hbox {S}_f$S $({\mathcal {H}},{\mathcal {Q}},5)$ can be identified as a CFF6$\hbox {S}_f$S over *U*. In a CFF6$\hbox {S}_f$S, we consider that there is a 5 grade, which is never used in Example 3.1.
4. In Definition 3.1, grade 0 describes the lowest score. It does not mean that there is incomplete information or no assessment.

### Definition 3.5

A $\hbox {CFFNS}_f$S $({\mathcal {H}},{\mathcal {Q}},N)$ over universe of discourse *U*,  where ${\mathcal {Q}}=({\mathcal {F}},{\mathcal {B}},N)$ is an $\hbox {NS}_f$S, is said to be *efficient* if ${\mathcal {H}}(b_t)=\langle (u_g,N-1),1e^{i2\pi },0e^{i0\pi }\rangle$ for some $b_t\in {\mathcal {B}},u_g\in U.$

### Example 3.2

By inspection, it can be checked that the CFF5$\hbox {S}_f$S defined in Example 3.1 is not efficient. However, CFF5$\hbox {S}_f$S $({\mathcal {H}},{\mathcal {Q}},5)$ in Table 7 is efficient.

**Table 7:** Tabular form of the efficient CFF5$\hbox {S}_f$S $({\mathcal {H}}, {\mathcal {Q}}, 5)$

$({\mathcal {H}}, {\mathcal {Q}}, 5)$	$b_{1}$	$b_{2}$	$b_{3}$
$x_{1}$	$\langle 2,(0.5e^{i0.9\pi },0.6e^{i1.2\pi })\rangle$	$\langle 3,(0.7e^{i1.5\pi },0.3e^{i0.5\pi })\rangle$	$\langle 1,(0.3e^{i0.6\pi },0.8e^{i1.6\pi })\rangle$
$x_{2}$	$\langle 1,(0.3e^{i0.5\pi },0.7e^{i1.5\pi })\rangle$	$\langle 3,(0.8e^{i1.6\pi },0.4e^{i0.6\pi })\rangle$	$\langle 2,(0.6e^{i0.9\pi },0.5e^{i1.2\pi })\rangle$
$x_{3}$	$\langle 3,(0.7e^{i1.4\pi },0.3e^{i0.5\pi })\rangle$	$\langle 4,(0.9e^{i1.8\pi },0.1e^{i0.3\pi })\rangle$	$\langle 0,(0.1e^{i0.2\pi },0.9e^{i1.9\pi })\rangle$
$x_{4}$	$\langle 0,(0.1e^{i0.2\pi },0.9e^{i1.8\pi })\rangle$	$\langle 2,(0.5e^{i0.9\pi },0.5e^{i1.0\pi })\rangle$	$\langle 4,(1.0e^{i2.0\pi },0.0e^{i0.0\pi })\rangle$

### Definition 3.6

Let $({\mathcal {H}}_1,{\mathcal {Q}}_1,N_1)$ and $({\mathcal {H}}_2,{\mathcal {Q}}_2,N_2)$ be two $\hbox {CFFNS}_f$Ss over universe of discourse *U*,  where ${\mathcal {Q}}_1=({\mathcal {F}}_1,{\mathcal {B}}_1,N_1),{\mathcal {Q}}_2=({\mathcal {F}}_2,{\mathcal {B}}_2,N_2)$ are $\hbox {NS}_f$Ss, then $({\mathcal {H}}_1,{\mathcal {Q}}_1,N_1)$ and $({\mathcal {H}}_2,{\mathcal {Q}}_2,N_2)$ are said to be *equal* if and only if ${\mathcal {H}}_1={\mathcal {H}}_2$ and ${\mathcal {Q}}_1={\mathcal {Q}}_2.$

We now define the concept of complementarity $\hbox {CFFNS}_f$S:

### Definition 3.7

Let $({\mathcal {H}},{\mathcal {Q}},N)$ be a CFFNS${_f}$S over universe of discourse *U*,  where ${\mathcal {Q}}=({\mathcal {F}},{\mathcal {B}},N)$ is an $\hbox {NS}_f$S, then $({\mathcal {H}},{\mathcal {Q}}^c,N)$ is said to be *weak complement* if ${\mathcal {Q}}=({\mathcal {F}}^c,{\mathcal {B}},N)$ is a weak complement of ${\mathcal {Q}}=({\mathcal {F}},{\mathcal {B}},N).$ By this mean that ${\mathcal {F}}^c(b_t) \cap {\mathcal {F}}(b_t)=\emptyset$ for all $b_t\in {\mathcal {B}}.$

The term weak complement is used because this complement is not unique.

### Definition 3.8

Let $({\mathcal {H}},{\mathcal {Q}},N)$ be a CFFNS${_f}$S over universe of discourse *U*,  where ${\mathcal {Q}}=({\mathcal {F}},{\mathcal {B}},N)$ is an $\hbox {NS}_f$S, then a *CFF complement* is denoted by $({\mathcal {H}}^c,{\mathcal {Q}},N),$ such that ${\mathcal {H}}^c$ is defined as ${\mathcal {H}}^c:{\mathcal {B}}\longrightarrow CFF^{(U\times {\mathcal {R}})},$ which is given by:

$$\begin{aligned} {\mathcal {H}}^c(b_t)=\{\langle (u_g,r_{a_{gt}}),k(u_g,r_{a_{gt}})e^{i\omega (u_g,r_{a_{gt}})}, s(u_g,r_{a_{gt}})e^{i\psi (u_g,r_{a_{gt}})}\rangle ~|~ b_t \in {\mathcal {B}}, (u_g,r_{a_{gt}})\in U\times {\mathcal {R}}\}. \end{aligned}$$

In CFF complement, the grades are same as in the original $\hbox {NS}_f$S, however all their membership and non-membership degrees are complementary.

### Definition 3.9

Let $({\mathcal {H}},{\mathcal {Q}},N)$ be a CFFNS${_f}$S over universe of discourse *U*,  where ${\mathcal {Q}}=({\mathcal {F}},{\mathcal {B}},N)$ is an $\hbox {NS}_f$S, then $({\mathcal {H}}^c,{\mathcal {Q}}^c,N)$ is said to be *weak CFF complement* when $({\mathcal {H}},{\mathcal {Q}}^c,N)$ is a weak complement and $({\mathcal {H}}^c,{\mathcal {Q}},N)$ is a CFF complement.

In other words, a weak CFF complement of CFFNS${_f}$S is the CFF complement of any of its weak complement.

### Example 3.3

A weak complement $({\mathcal {H}},{\mathcal {Q}}^c,5)$ of CFF5$\hbox {S}_f$S in Example 3.1, Table 6, is represented by Table 8. The CFF complement of this CFF5$\hbox {S}_f$S is $({\mathcal {H}}^c,{\mathcal {Q}},5)$ defined by Table 9. A weak CFF complement of this CFF5$\hbox {S}_f$S is $({\mathcal {H}}^c,{\mathcal {Q}}^c,5)$ defined by Table 10.

**Table 8:** A weak complement of the CFF5$\hbox {S}_f$S $({\mathcal {H}}, {\mathcal {Q}}, 5)$ in Example 3.1

$(H, {\mathcal {Q}}^c, 5)$	$b_{1}$	$b_{2}$	$b_{3}$
$x_{1}$	$\langle 3,(0.5e^{i0.9\pi },0.6e^{i1.2\pi })\rangle$	$\langle 4,(0.7e^{i1.5\pi },0.3e^{i0.5\pi })\rangle$	$\langle 2,(0.3e^{i0.6\pi },0.8e^{i1.6\pi })\rangle$
$x_{2}$	$\langle 0,(0.3e^{i0.5\pi },0.7e^{i1.5\pi })\rangle$	$\langle 2,(0.8e^{i1.6\pi },0.4e^{i0.6\pi })\rangle$	$\langle 3,(0.6e^{i0.9\pi },0.5e^{i1.2\pi })\rangle$
$x_{3}$	$\langle 2,(0.7e^{i1.4\pi },0.3e^{i0.5\pi })\rangle$	$\langle 3,(0.9e^{i1.8\pi },0.1e^{i0.3\pi })\rangle$	$\langle 2,(0.1e^{i0.2\pi },0.9e^{i1.9\pi })\rangle$
$x_{4}$	$\langle 4,(0.1e^{i0.2\pi },0.9e^{i1.8\pi })\rangle$	$\langle 1,(0.5e^{i0.9\pi },0.5e^{i1.0\pi })\rangle$	$\langle 3,(0.9e^{i1.9\pi },0.2e^{i0.3\pi })\rangle$

**Table 9:** Tabular representation of the CFF complement of the CFF5$\hbox {S}_f$S $({\mathcal {H}}, {\mathcal {Q}}, 5)$ in Example 3.1

$(H^c, {\mathcal {Q}}, 5)$	$b_{1}$	$b_{2}$	$b_{3}$
$x_{1}$	$\langle 2,(0.6e^{i1.2\pi },0.5e^{i0.9\pi })\rangle$	$\langle 3,(0.3e^{i0.5\pi },0.7e^{i1.5\pi })\rangle$	$\langle 1,(0.8e^{i1.6\pi },0.3e^{i0.6\pi })\rangle$
$x_{2}$	$\langle 1,(0.7e^{i1.5\pi },0.3e^{i0.5\pi })\rangle$	$\langle 3,(0.4e^{i0.6\pi },0.8e^{i1.6\pi })\rangle$	$\langle 2,(0.5e^{i1.2\pi },0.6e^{i0.9\pi })\rangle$
$x_{3}$	$\langle 3,(0.3e^{i0.5\pi },0.7e^{i1.4\pi })\rangle$	$\langle 4,(0.1e^{i0.3\pi },0.9e^{i1.8\pi })\rangle$	$\langle 0,(0.9e^{i1.9\pi },0.1e^{i0.2\pi })\rangle$
$x_{4}$	$\langle 0,(0.9e^{i1.8\pi },0.1e^{i0.2\pi })\rangle$	$\langle 2,(0.5e^{i1.0\pi },0.5e^{i0.9\pi })\rangle$	$\langle 4,(0.2e^{i0.3\pi },0.9e^{i1.9\pi })\rangle$

**Table 10:** Tabular representation of a weak CFF complement of the CFF5$\hbox {S}_f$S $({\mathcal {H}}, {\mathcal {Q}}, 5)$ in Example 3.1

$(H^c, {\mathcal {Q}}^c, 5)$	$b_{1}$	$b_{2}$	$b_{3}$
$x_{1}$	$\langle 3,(0.6e^{i1.2\pi },0.5e^{i0.9\pi })\rangle$	$\langle 4,(0.3e^{i0.5\pi },0.7e^{i1.5\pi })\rangle$	$\langle 2,(0.8e^{i1.6\pi },0.3e^{i0.6\pi })\rangle$
$x_{2}$	$\langle 0,(0.7e^{i1.5\pi },0.3e^{i0.5\pi })\rangle$	$\langle 2,(0.4e^{i0.6\pi },0.8e^{i1.6\pi })\rangle$	$\langle 3,(0.5e^{i1.2\pi },0.6e^{i0.9\pi })\rangle$
$x_{3}$	$\langle 2,(0.3e^{i0.5\pi },0.7e^{i1.4\pi })\rangle$	$\langle 3,(0.1e^{i0.3\pi },0.9e^{i1.8\pi })\rangle$	$\langle 2,(0.9e^{i1.9\pi },0.1e^{i0.2\pi })\rangle$
$x_{4}$	$\langle 4,(0.9e^{i1.8\pi },0.1e^{i0.2\pi })\rangle$	$\langle 1,(0.5e^{i1.0\pi },0.5e^{i0.9\pi })\rangle$	$\langle 3,(0.2e^{i0.3\pi },0.9e^{i1.9\pi })\rangle$

### Definition 3.10

For a CFFNS${_f}$S $({\mathcal {H}},{\mathcal {Q}},N),$ where ${\mathcal {Q}}=({\mathcal {F}},{\mathcal {B}},N)$ is an $\hbox {NS}_f$S, the *top weak complement* of $({\mathcal {H}},{\mathcal {Q}},N)$ is $({\mathcal {H}},{\mathcal {Q}}^>,N),$ and the *top weak CFF complement* of $({\mathcal {H}},{\mathcal {Q}},N)$ is $({\mathcal {H}}^c,{\mathcal {Q}}^>,N),$ where ${\mathcal {Q}}^>=({\mathcal {F}}^>,{\mathcal {B}},N)$ is the top weak complement of ${\mathcal {Q}}=({\mathcal {F}},{\mathcal {B}},N)$ and defined as follows:

${\mathcal {F}}^>(u_g)(b_t)$ (Fatimah et al. 2018) $$=\left\{ \begin{array}{ll} N-1, &{} \hbox {if }{\mathcal {F}}(u_g)(b_t)<N-1, \\ 0, &{} \hbox {if }{\mathcal {F}}(u_g)(b_t)=N-1. \end{array} \right.$$

$$({\mathcal {H}},{\mathcal {Q}}^>,N)=\left\{ \begin{array}{ll} \langle N-1,(s_{a_{gt}}e^{i\omega _{a_{gt}}},k_{a_{gt}}e^{i\psi _{a_{gt}}}) \rangle , &{} \hbox {if }r_{a_{gt}}<N-1, \\ \langle 0,(s_{a_{gt}}e^{i\omega _{a_{gt}}},k_{a_{gt}}e^{i\psi _{a_{gt}}}) \rangle , &{} \hbox {if }r_{a_{gt}}=N-1. \end{array} \right.$$

$$({\mathcal {H}}^c,{\mathcal {Q}}^>,N)=\left\{ \begin{array}{ll} \langle N-1, (k_{a_{gt}}e^{i\psi _{a_{gt}}},s_{a_{gt}} e^{i\omega _{a_{gt}}})\rangle , &{} \hbox {if }r_{a_{gt}}<N-1, \\ \langle 0,(k_{a_{gt}}e^{i\psi _{a_{gt}}},s_{a_{gt}}e^{i\omega _{a_{gt}}})\rangle , &{} \hbox {if }r_{a_{gt}}=N-1. \end{array} \right.$$

### Example 3.4

The top weak complement and the top weak CFF complement of the CFF5$\hbox {S}_f$S Table 6 in Example 3.1 are given by Tables 11 and 12.

**Table 11:** Tabular representation of the top weak complement of CFF5$\hbox {S}_f$S $({\mathcal {H}}, {\mathcal {Q}}, 5)$ in Example 3.1

$(H, {\mathcal {Q}}^>, 5)$	$b_{1}$	$b_{2}$	$b_{3}$
$x_{1}$	$\langle 4,(0.5e^{i0.9\pi },0.6e^{i1.2\pi })\rangle$	$\langle 4,(0.7e^{i1.5\pi },0.3e^{i0.5\pi })\rangle$	$\langle 4,(0.3e^{i0.6\pi },0.8e^{i1.6\pi })\rangle$
$x_{2}$	$\langle 4,(0.3e^{i0.5\pi },0.7e^{i1.5\pi })\rangle$	$\langle 4,(0.8e^{i1.6\pi },0.4e^{i0.6\pi })\rangle$	$\langle 4,(0.6e^{i0.9\pi },0.5e^{i1.2\pi })\rangle$
$x_{3}$	$\langle 4,(0.7e^{i1.4\pi },0.3e^{i0.5\pi })\rangle$	$\langle 0,(0.9e^{i1.8\pi },0.1e^{i0.3\pi })\rangle$	$\langle 4,(0.1e^{i0.2\pi },0.9e^{i1.9\pi })\rangle$
$x_{4}$	$\langle 4,(0.1e^{i0.2\pi },0.9e^{i1.8\pi })\rangle$	$\langle 4,(0.5e^{i0.9\pi },0.5e^{i1.0\pi })\rangle$	$\langle 0,(0.9e^{i1.9\pi },0.2e^{i0.3\pi })\rangle$

**Table 12:** Tabular representation of the top weak CFF complement of the CFF5$\hbox {S}_f$S $({\mathcal {H}}, {\mathcal {Q}}, 5)$ in Example 3.1

$(H^c, {\mathcal {Q}}^>, 5)$	$b_{1}$	$b_{2}$	$b_{3}$
$x_{1}$	$\langle 4,(0.6e^{i1.2\pi },0.5e^{i0.9\pi })\rangle$	$\langle 4,(0.3e^{i0.5\pi },0.7e^{i1.5\pi })\rangle$	$\langle 4,(0.8e^{i1.6\pi },0.3e^{i0.6\pi })\rangle$
$x_{2}$	$\langle 4,(0.7e^{i1.5\pi },0.3e^{i0.5\pi })\rangle$	$\langle 4,(0.4e^{i0.6\pi },0.8e^{i1.6\pi })\rangle$	$\langle 4,(0.5e^{i1.2\pi },0.6e^{i0.9\pi })\rangle$
$x_{3}$	$\langle 4,(0.3e^{i0.5\pi },0.7e^{i1.4\pi })\rangle$	$\langle 0,(0.1e^{i0.3\pi },0.9e^{i1.8\pi })\rangle$	$\langle 4,(0.9e^{i1.9\pi },0.1e^{i0.2\pi })\rangle$
$x_{4}$	$\langle 4,(0.9e^{i1.8\pi },0.1e^{i0.2\pi })\rangle$	$\langle 4,(0.5e^{i1.0\pi },0.5e^{i0.9\pi })\rangle$	$\langle 0,(0.2e^{i0.3\pi },0.9e^{i1.9\pi })\rangle$

### Definition 3.11

For a CFFNS${_f}$S $({\mathcal {H}},{\mathcal {Q}},N),$ where ${\mathcal {Q}}=({\mathcal {F}},{\mathcal {B}},N)$ is an $\hbox {NS}_f$S, the *bottom weak complement* of $({\mathcal {H}},{\mathcal {Q}},N)$ is $({\mathcal {H}},{\mathcal {Q}}^<,N),$ and the *bottom weak CFF complement* of $({\mathcal {H}},{\mathcal {Q}},N)$ is $({\mathcal {H}}^c,{\mathcal {Q}}^<,N),$ where ${\mathcal {Q}}^<=({\mathcal {F}}^<,{\mathcal {B}},N)$ is the bottom weak complement of ${\mathcal {Q}}=({\mathcal {F}},{\mathcal {B}},N)$ and defined as follows:

${\mathcal {F}}^<(u_g)(b_t)$ (Fatimah et al. 2018) $$=\left\{ \begin{array}{ll} 0, &{} \hbox {if }{\mathcal {F}}(u_g)(b_t)>0, \\ N-1, &{} \hbox {if }{\mathcal {F}}(u_g)(b_t)=0. \end{array} \right.$$

$$({\mathcal {H}},{\mathcal {Q}}^<,N)=\left\{ \begin{array}{ll} \langle 0,(s_{a_{gt}}e^{i\omega _{a_{gt}}},k_{a_{gt}}e^{i\psi _{a_{gt}}}) \rangle , &{} \hbox {if }r_{a_{gt}}>0, \\ \langle N-1,(s_{a_{gt}}e^{i\omega _{a_{gt}}},k_{a_{gt}}e^{i\psi _{a_{gt}}})\rangle , &{} \hbox {if }r_{a_{gt}}=0. \end{array} \right.$$

$$({\mathcal {H}}^c,{\mathcal {Q}}^<,N)=\left\{ \begin{array}{ll} \langle 0,(k_{a_{gt}}e^{i\psi _{a_{gt}}},s_{a_{gt}}e^{i\omega _{a_{gt}}}) \rangle , &{} \hbox {if }r_{a_{gt}}>0, \\ \langle N-1,(k_{a_{gt}}e^{i\psi _{a_{gt}}},s_{a_{gt}}e^{i\omega _{a_{gt}}})\rangle , &{} \hbox {if }r_{a_{gt}}=0. \end{array} \right.$$

### Example 3.5

The bottom weak complement and the bottom weak CFF complement of the CFF5$\hbox {S}_f$S Table 6 in Example 3.1 are given by Tables 13 and 14 .

**Table 13:** Tabular representation of the bottom weak complement of CFF5$\hbox {S}_f$S $({\mathcal {H}}, {\mathcal {Q}}, 5)$ in Example 3.1

$({\mathcal {H}}, {\mathcal {Q}}^<, 5)$	$b_{1}$	$b_{2}$	$b_{3}$
$x_{1}$	$\langle 0,(0.5e^{i0.9\pi },0.6e^{i1.2\pi })\rangle$	$\langle 0,(0.7e^{i1.5\pi },0.3e^{i0.5\pi })\rangle$	$\langle 0,(0.3e^{i0.6\pi },0.8e^{i1.6\pi })\rangle$
$x_{2}$	$\langle 0,(0.3e^{i0.5\pi },0.7e^{i1.5\pi })\rangle$	$\langle 0,(0.8e^{i1.6\pi },0.4e^{i0.6\pi })\rangle$	$\langle 0,(0.6e^{i0.9\pi },0.5e^{i1.2\pi })\rangle$
$x_{3}$	$\langle 0,(0.7e^{i1.4\pi },0.3e^{i0.5\pi })\rangle$	$\langle 0,(0.9e^{i1.8\pi },0.1e^{i0.3\pi })\rangle$	$\langle 4,(0.1e^{i0.2\pi },0.9e^{i1.9\pi })\rangle$
$x_{4}$	$\langle 4,(0.1e^{i0.2\pi },0.9e^{i1.8\pi })\rangle$	$\langle 0,(0.5e^{i0.9\pi },0.5e^{i1.0\pi })\rangle$	$\langle 0,(0.9e^{i1.9\pi },0.2e^{i0.3\pi })\rangle$

**Table 14:** Tabulated form of the bottom weak CFF complement of the CFF5$\hbox {S}_f$S $({\mathcal {H}}, {\mathcal {Q}}, 5)$ in Example 3.1

$({\mathcal {H}}^c, {\mathcal {Q}}^<, 5)$	$b_{1}$	$b_{2}$	$b_{3}$
$x_{1}$	$\langle 0,(0.6e^{i1.2\pi },0.5e^{i0.9\pi })\rangle$	$\langle 0,(0.3e^{i0.5\pi },0.7e^{i1.5\pi })\rangle$	$\langle 0,(0.8e^{i1.6\pi },0.3e^{i0.6\pi })\rangle$
$x_{2}$	$\langle 0,(0.7e^{i1.5\pi },0.3e^{i0.5\pi })\rangle$	$\langle 0,(0.4e^{i0.6\pi },0.8e^{i1.6\pi })\rangle$	$\langle 0,(0.5e^{i1.2\pi },0.6e^{i0.9\pi })\rangle$
$x_{3}$	$\langle 0,(0.3e^{i0.5\pi },0.7e^{i1.4\pi })\rangle$	$\langle 0,(0.1e^{i0.3\pi },0.9e^{i1.8\pi })\rangle$	$\langle 4,(0.9e^{i1.9\pi },0.1e^{i0.2\pi })\rangle$
$x_{4}$	$\langle 4,(0.9e^{i1.8\pi },0.1e^{i0.2\pi })\rangle$	$\langle 0,(0.5e^{i1.0\pi },0.5e^{i0.9\pi })\rangle$	$\langle 0,(0.2e^{i0.3\pi },0.9e^{i1.9\pi })\rangle$

### Definition 3.12

Let *U* be a universe of discourse and $({\mathcal {H}}_1,{\mathcal {Q}}_1,N_1)$ and $({\mathcal {H}}_2,{\mathcal {Q}}_2,N_2)$ be two CFFNS${_f}$Ss over non-empty set *U*,  where ${\mathcal {Q}}_1=({\mathcal {F}}_1,{\mathcal {B}}_1,N_1)$ and ${\mathcal {Q}}_2=({\mathcal {F}}_2,{\mathcal {B}}_2,N_2)$ are $\hbox {NS}_f$Ss on *U*,  then their *restricted intersection* is denoted by $({\mathcal {H}}_1,{\mathcal {Q}}_1,N_1) \cap _{\mathfrak {R}} ({\mathcal {H}}_2,{\mathcal {Q}}_2,N_2)$ and is defined as $(\sigma ,{\mathcal {Q}}_1 ~\cap _{\mathfrak {r}}~ {\mathcal {Q}}_2 ,\min {(N_1,N_2)}),$ where ${\mathcal {Q}}_1 \cap _{\mathfrak {r}} {\mathcal {Q}}_2=({\mathcal {F}},{\mathcal {B}}_1\cap {\mathcal {B}}_2, \min (N_1,N_2))$ for all $b_t\in {\mathcal {B}}_1\cap {\mathcal {B}}_2,~ u_g\in U,\langle (u_g,r_{a_{gt}}),y,z\rangle \in \sigma (b_{t}) \Leftrightarrow r_{a_{gt}}=\min (r_{a_{gt}}^1,r_{a_{gt}}^2),~ y=\min (s_{{\mathcal {C}}}(u_g,r_{a_{gt}}^1),s_{{\mathcal {D}}} (u_g,r_{a_{gt}}^2)) e^{i\min (\omega _{{\mathcal {C}}}(u_g,r_{a_{gt}}^1),\omega _{{{\mathcal {D}}}} (u_g,r_{a_{gt}}^2))},~ z=\max (k_{{\mathcal {C}}}(u_g,r_{a_{gt}}^1),k_{{\mathcal {D}}} (u_g,r_{a_{gt}}^2))e^{i\max (\psi _{{\mathcal {C}}}(u_g,r_{a_{gt}}^1), \psi _{{{\mathcal {D}}}}(u_g,r_{a_{gt}}^2))},$ if

$\langle (u_g,r_{a_{gt}}^1), s_{{\mathcal {C}}}(u_g,r_{a_{gt}}^1)e^{i\omega _{{\mathcal {C}}} (u_g,r_{a_{gt}}^1)},k_{{\mathcal {C}}}(u_g,r_{a_{gt}}^1) e^{ i\psi _{{\mathcal {C}}}(u_g,r_{a_{gt}}^1)}\rangle \in {\mathcal {B}}_1(b_{t})$ and $\langle (u_g,r_{a_{gt}}^2),s_{{\mathcal {D}}}(u_g,r_{a_{gt}}^2) e^{i\omega _{{{\mathcal {D}}}}(u_g,r_{a_{gt}}^2)}, k_{{\mathcal {D}}}(u_g,r_{a_{gt}}^2)e^{ i\psi _{{\mathcal {D}}}(u,r_{a_{gt}}^2)}\rangle \in {\mathcal {B}}_2(b_{t}),~{\mathcal {C}}$ and ${\mathcal {D}}$ are CFFSs on ${\mathcal {F}}_{1}(b_t)$ and ${\mathcal {F}}_{2}(b_t),$ respectively.

### Example 3.6

Let $({\mathcal {H}}_1,{\mathcal {Q}}_1,5)$ be a CFF5$\hbox {S}_f$S and $({\mathcal {H}}_2,{\mathcal {Q}}_2,4)$ be a CFF4$\hbox {S}_f$S defined by Tables 15 and 16 , respectively, where ${\mathcal {Q}}_1=({\mathcal {F}}_1,{\mathcal {B}}_1,5)$ and ${\mathcal {Q}}_2=({\mathcal {H}}_2,{\mathcal {B}}_2,4)$ are $\hbox {NS}_f$Ss over *U*,  then their restricted intersection $({\mathcal {H}}_1,{\mathcal {Q}}_1,5) \cap _{\mathfrak {R}} ({\mathcal {H}}_2,{\mathcal {Q}}_2,4)=(\sigma ,{\mathcal {Q}}_1 ~\cap _{\mathfrak {r}}~ {\mathcal {Q}}_2 ,4)$ is defined by Table 17.

**Table 15:** Tabular representation of CFF5$\hbox {S}_f$S $({\mathcal {H}}_1,{\mathcal {Q}}_1,5)$ in Example 3.6

$({\mathcal {H}}_1, {\mathcal {Q}}_1, 5)$	$b_{1}$	$b_{2}$	$b_{3}$
$x_{1}$	$\langle 4,(0.92e^{i1.88\pi },0.08e^{i0.36\pi })\rangle$	$\langle 3,(0.77e^{i1.58\pi },0.37e^{i0.69\pi })\rangle$	$\langle 2,(0.61e^{i1.14\pi },0.42e^{i1.29\pi })\rangle$
$x_{2}$	$\langle 3,(0.73e^{i1.46\pi },0.27e^{i0.78\pi })\rangle$	$\langle 4,(0.92e^{i1.96\pi },0.15e^{i0.29\pi })\rangle$	$\langle 1,(0.37e^{i0.63\pi },0.59e^{i1.54\pi })\rangle$
$x_{3}$	$\langle 4,(0.89e^{i1.76\pi },0.09e^{i0.36\pi })\rangle$	$\langle 2,(0.49e^{i1.27\pi },0.57e^{i1.18\pi })\rangle$	$\langle 0,(0.05e^{i0.32\pi },0.92e^{i1.94\pi })\rangle$
$x_{4}$	$\langle 1,(0.25e^{i0.45\pi },0.75e^{i1.65\pi })\rangle$	$\langle 0,(0.15e^{i0.25\pi },0.95e^{i1.85\pi })\rangle$	$\langle 2,(0.55e^{i0.95\pi },0.61e^{i0.85\pi })\rangle$

**Table 16:** Tabulated form of the CFF4$\hbox {S}_f$S $({\mathcal {H}}_2, {\mathcal {Q}}_2, 4)$ in Example 3.6

$({\mathcal {H}}_2, {\mathcal {Q}}_2, 4)$	$b_{1}$	$b_{2}$	$\upsilon$
$x_{1}$	$\langle 0,(0.05e^{i0.42\pi },0.85e^{i1.76\pi })\rangle$	$\langle 2,(0.48e^{i1.57\pi },0.29e^{i0.67\pi })\rangle$	$\langle 3,(0.88e^{i1.76\pi },0.05e^{i0.19\pi })\rangle$
$x_{2}$	$\langle 1,(0.33e^{i0.57\pi },0.69e^{i1.48\pi })\rangle$	$\langle 3,(0.97e^{i1.96\pi },0.19e^{i0.39\pi })\rangle$	$\langle 2,(0.69e^{i0.97\pi },0.38e^{i0.65\pi })\rangle$
$x_{3}$	$\langle 2,(0.76e^{i1.43\pi },0.39e^{i0.66\pi })\rangle$	$\langle 0,(0.19e^{i0.25\pi },0.89e^{i1.94\pi })\rangle$	$\langle 1,(0.29e^{i0.72\pi },0.58e^{i1.33\pi })\rangle$
$x_{4}$	$\langle 3,(0.93e^{i1.88\pi },0.15e^{i0.25\pi })\rangle$	$\langle 1,(0.38e^{i0.67\pi },0.76e^{i1.29\pi })\rangle$	$\langle 0,(0.12e^{i0.37\pi },0.91e^{i1.87\pi })\rangle$

**Table 17:** Tabulated form of $({\mathcal {H}}_1,{\mathcal {Q}}_1,5) \cap _{\mathfrak {R}} ({\mathcal {H}}_2,{\mathcal {Q}}_2,4)$ defined in Example 3.6

$(\sigma ,{\mathcal {Q}}_1 ~\cap _{\mathfrak {r}}~ {\mathcal {Q}}_2 ,4)$	$b_{1}$	$b_{2}$
$x_{1}$	$\langle 0,(0.05e^{i0.42\pi },0.85e^{i1.76\pi })\rangle$	$\langle 2,(0.48e^{i1.57\pi },0.37e^{i0.69\pi })\rangle$
$x_{2}$	$\langle 1,(0.33e^{i0.57\pi },0.69e^{i1.48\pi })\rangle$	$\langle 3,(0.92e^{i1.96\pi },0.19e^{i0.39\pi })\rangle$
$x_{3}$	$\langle 2,(0.76e^{i1.43\pi },0.39e^{i0.66\pi })\rangle$	$\langle 0,(0.19e^{i0.25\pi },0.89e^{i1.94\pi })\rangle$
$x_{4}$	$\langle 1,(0.25e^{i0.45\pi },0.75e^{i1.65\pi })\rangle$	$\langle 0,(0.15e^{i0.25\pi },0.95e^{i1.85\pi })\rangle$

### Definition 3.13

Let *U* be a universe of discourse and $({\mathcal {H}}_1,{\mathcal {Q}}_1,N_1)$ and $({\mathcal {H}}_2,{\mathcal {Q}}_2,N_2)$ be two CFFNS${_f}$Ss over non-empty set *U*,  where ${\mathcal {Q}}_1=({\mathcal {F}}_1,{\mathcal {B}}_1,N_1)$ and ${\mathcal {Q}}_2=({\mathcal {F}}_2,{\mathcal {B}}_2,N_2)$ are $\hbox {NS}_f$Ss on *U*,  then their *extended intersection* is denoted by $({\mathcal {H}}_1,{\mathcal {Q}}_1,N_1) \cap _{\mathfrak {E}} ({\mathcal {H}}_2,{\mathcal {Q}}_2,N_2)$ and is defined as $(\Im ,{\mathcal {Q}}_1 ~\cap _{\mathfrak {e}}~ {\mathcal {Q}}_2 ,\max (N_1,N_2)),$ where ${\mathcal {Q}}_1 \cap _{\mathfrak {e}} {\mathcal {Q}}_2=({\mathcal {H}},{\mathcal {B}}_1\cup {\mathcal {B}}_2, \max (N_1,N_2)),$ and $\Im (b_t)$ is given by:

$$\Im (b_t)=\left\{ \begin{array}{ll} {\mathcal {H}}_1(b_t), &{} \hbox {if }b_t\in {\mathcal {B}}_1-{\mathcal {B}}_2, \\ {\mathcal {H}}_2(b_t), &{} \hbox {if } b_t\in {\mathcal {B}}_1-{\mathcal {B}}_2, \\ \langle (u_g,r_{a_{gt}}),y,z\rangle , &{} \hbox {such that }r_{a_{gt}}=\min (r_{a_{gt}}^1,r_{a_{gt}}^2),\\ &{}{y=\min (s_{{\mathcal {C}}}(u_g,r_{a_{gt}}^1),s_{{\mathcal {D}}} (u_g,r_{a_{gt}}^2))e^{i\min (\omega _{{{\mathcal {C}}}}(u_g,r_{a_{gt}}^1), \omega _{{{\mathcal {D}}}}(u_g,r_{a_{gt}}^2))},}\\ &{}{z=\max (k_{{\mathcal {C}}}(u_g,r_{a_{gt}}^1),k_{{\mathcal {D}}} (u_g,r_{a_{gt}}^2))e^{i\max (\psi _{{{\mathcal {C}}}}(u_g,r_{a_{gt}}^1), \psi _{{{\mathcal {D}}}}(u_g,r_{a_{gt}}^2))},}\\ &{}\hbox {where }\langle (u_g,r_{a_{gt}}^1),s_{{\mathcal {C}}}(u_g,r_{a_{gt}}^1) e^{i\omega _{{{\mathcal {C}}}}(u_g,r_{a_{gt}}^1)},k_{{\mathcal {C}}} (u_g,r_{a_{gt}}^1)e^{i\psi _{{{\mathcal {C}}}}(u_g,r_{a_{gt}}^1)}\rangle \in {\mathcal {B}}_1(b_{t}) \\ &{}\hbox {and }\langle (u_g,r_{a_{gt}}^2),s_{{\mathcal {D}}}(u_g,r_{a_{gt}}^2) e^{i\omega _{{{\mathcal {D}}}}(u_g,r_{a_{gt}}^2)},k_{{\mathcal {D}}} (u_g,r_{a_{gt}}^2)e^{i\psi _{{{\mathcal {D}}}}(u_g,r_{a_{gt}}^2)}\rangle \in {\mathcal {B}}_2(b_{t}),\\ &{}{\mathcal {C}} \hbox { and }{\mathcal {D}} are CFFSs on {\mathcal {F}}_{1}(b_t)\hbox { and }{\mathcal {F}}_{2}(b_t), \hbox { respectively}. \end{array} \right.$$

### Example 3.7

The extended intersection $(\Im ,{\mathcal {Q}}_1 ~\cap _{\mathfrak {e}}~ {\mathcal {Q}}_2 ,5)$ of $({\mathcal {H}}_1,{\mathcal {Q}}_1,5)$ (see Table 15) and $({\mathcal {H}}_2,{\mathcal {Q}}_2,4)$ (see Table 16) is shown by Table 18.

**Table 18:** Tabulated form of the extended intersection $({\mathcal {H}}_1,{\mathcal {Q}}_1,5) \cap _{\mathfrak {E}} ({\mathcal {H}}_2,{\mathcal {Q}}_2,4)$ in Example 3.7

$(\Im ,{\mathcal {Q}}_1 ~\cap _{\mathfrak {e}}~ {\mathcal {Q}}_2 ,5)$	$b_{1}$	$b_{2}$	$b_{3}$	$\upsilon$
$x_{1}$	$\langle 0,(0.05e^{i0.42\pi },0.85e^{i1.76\pi })\rangle$	$\langle 2,(0.48e^{i1.57\pi },0.37e^{i0.69\pi })\rangle$	$\langle 2,(0.61e^{i1.14\pi },0.42e^{i1.29\pi })\rangle$	$\langle 3,(0.88e^{i1.76\pi },0.05e^{i0.19\pi })\rangle$
$x_{2}$	$\langle 1,(0.33e^{i0.57\pi },0.69e^{i1.48\pi })\rangle$	$\langle 3,(0.92e^{i1.96\pi },0.19e^{i0.39\pi })\rangle$	$\langle 1,(0.37e^{i0.63\pi },0.59e^{i1.54\pi })\rangle$	$\langle 2,(0.69e^{i0.97\pi },0.38e^{i0.65\pi })\rangle$
$x_{3}$	$\langle 2,(0.76e^{i1.43\pi },0.39e^{i0.66\pi })\rangle$	$\langle 0,(0.19e^{i0.25\pi },0.89e^{i1.94\pi })\rangle$	$\langle 0,(0.05e^{i0.32\pi },0.92e^{i1.94\pi })\rangle$	$\langle 1,(0.29e^{i0.72\pi },0.58e^{i1.33\pi })\rangle$
$x_{4}$	$\langle 1,(0.25e^{i0.45\pi },0.75e^{i1.65\pi })\rangle$	$\langle 0,(0.15e^{i0.25\pi },0.95e^{i1.85\pi })\rangle$	$\langle 2,(0.55e^{i0.95\pi },0.61e^{i0.85\pi })\rangle$	$\langle 0,(0.12e^{i0.37\pi },0.91e^{i1.87\pi })\rangle$

### Definition 3.14

Let *U* be a universe of discourse and $({\mathcal {H}}_1,{\mathcal {Q}}_1,N_1)$ and $({\mathcal {H}}_2,{\mathcal {Q}}_2,N_2)$ be two CFFNS${_f}$Ss over non-empty set *U*,  where ${\mathcal {Q}}_1=({\mathcal {F}}_1,{\mathcal {B}}_1,N_1)$ and ${\mathcal {Q}}_2=({\mathcal {F}}_2,{\mathcal {B}}_2,N_2)$ are $\hbox {NS}_f$Ss on *U*,  then their *restricted union* is denoted by $({\mathcal {F}}_1,{\mathcal {Q}}_1,N_1) \cup _{\mathfrak {R}} ({\mathcal {F}}_2,{\mathcal {Q}}_2,N_2)$ and is defined as $(\hbar ,{\mathcal {Q}}_1 ~\cup _\mathfrak {\Re }~ {\mathcal {Q}}_2, \max (N_1,N_2)),$ where ${\mathcal {Q}}_1 \cup _\mathfrak {\Re } {\mathcal {Q}}_2=({\mathcal {S}},{\mathcal {B}}_1\cap {\mathcal {B}}_2, \max (N_1,N_2))$ for all $b_t\in {\mathcal {B}}_1\cap {\mathcal {B}}_2,~ u_g\in U, \langle (u_g,r_{a_{gt}}),y,z\rangle \in \hbar (b_{t}) \Leftrightarrow r_{a_{gt}}=\max (r_{a_{gt}}^1,r_{a_{gt}}^2),~ y=\max (s_{{\mathcal {C}}}(u_g,r_{a_{gt}}^1),s_{{\mathcal {D}}} (u_g,r_{a_{gt}}^2)) e^{i\max (\omega _{{{\mathcal {C}}}}(u_g,r_{a_{gt}}^1), \omega _{{{\mathcal {D}}}}(u_g,r_{a_{gt}}^2))}, z=\min (k_{{\mathcal {C}}}(u_g,r_{a_{gt}}^1),k_{{\mathcal {D}}} (u_g,r_{a_{gt}}^2))e^{i\min (\psi _{{{\mathcal {C}}}}(u_g,r_{a_{gt}}^1), \psi _{{{\mathcal {D}}}}(u_g,r_{a_{gt}}^2))},$ if

$\langle (u_g,r_{a_{gt}}^1),s_{{\mathcal {C}}}(u_g,r_{a_{gt}}^1) e^{i\omega _{{{\mathcal {C}}}}(u_g,r_{a_{gt}}^1)},k_{{\mathcal {C}}} (u_g,r_{a_{gt}}^1)e^{i\psi _{{{\mathcal {C}}}}(u_g,r_{a_{gt}}^1)}\rangle \in {\mathcal {B}}_1(b_{t})$ and $\langle (u_g,r_{a_{gt}}^2),s_{{\mathcal {D}}}(u_g,r_{a_{gt}}^2) e^{i\omega _{{{\mathcal {D}}}}(u_g,r_{a_{gt}}^2)},k_{{\mathcal {D}}} (u_g,r_{a_{gt}}^2)e^{i\psi _{{{\mathcal {D}}}}(u_g,r_{a_{gt}}^2)}\rangle \in {\mathcal {B}}_2(b_{t}),~{\mathcal {C}}$ and ${\mathcal {D}}$ are CFFSs on ${\mathcal {F}}_{1}(b_t)$ and ${\mathcal {F}}_{2}(b_t),$ respectively.

### Example 3.8

The restricted union $(\hbar ,{\mathcal {Q}}_1 ~\cup _\mathfrak {\Re }~ {\mathcal {Q}}_2 ,5)$ of $({\mathcal {H}}_1,{\mathcal {Q}}_1,5)$ (see Table 15) and $({\mathcal {H}}_2,{\mathcal {Q}}_2,4)$ (see Table 16) is defined by Table 19.

**Table 19:** Tabulated form of $({\mathcal {H}}_1,{\mathcal {Q}}_1,5)\cup _{\mathfrak {R}}({\mathcal {H}}_2,{\mathcal {Q}}_2,4)$ in Example 3.8

$(\hbar ,{\mathcal {Q}}_1 \cup _\mathfrak {\Re } {\mathcal {Q}}_2 ,5)$	$b_{1}$	$b_{2}$
$x_{1}$	$\langle 4,(0.92e^{i1.88\pi },0.08e^{i0.36\pi })\rangle$	$\langle 3,(0.77e^{i1.58\pi },0.29e^{i0.67\pi })\rangle$
$x_{2}$	$\langle 3,(0.73e^{i1.46\pi },0.27e^{i0.78\pi })\rangle$	$\langle 4,(0.97e^{i1.96\pi },0.15e^{i0.29\pi })\rangle$
$x_{3}$	$\langle 4,(0.89e^{i1.76\pi },0.09e^{i0.36\pi })\rangle$	$\langle 2,(0.49e^{i1.27\pi },0.57e^{i1.18\pi })\rangle$
$x_{4}$	$\langle 3,(0.93e^{i1.88\pi },0.15e^{i0.25\pi })\rangle$	$\langle 1,(0.38e^{i0.67\pi },0.76e^{i1.29\pi })\rangle$

### Definition 3.15

Let *U* be a universe of discourse and $({\mathcal {H}}_1,{\mathcal {Q}}_1,N_1)$ and $({\mathcal {H}}_2,{\mathcal {Q}}_2,N_2)$ be two CFFNS${_f}$Ss over non-empty set *U*,  where ${\mathcal {Q}}_1=({\mathcal {F}}_1,{\mathcal {B}}_1,N_1)$ and ${\mathcal {Q}}_2=({\mathcal {F}}_2,{\mathcal {B}}_2,N_2)$ are $\hbox {NS}_f$Ss on *U*,  then their *extended union* is denoted by $({\mathcal {H}}_1,{\mathcal {Q}}_1,N_1) \cup _{\mathfrak {E}} ({\mathcal {H}}_2,{\mathcal {Q}}_2,N_2)$ and is defined as $(\zeta ,{\mathcal {Q}}_1 ~\cup _\mathfrak {\epsilon }~ {\mathcal {Q}}_2 ,\max (N_1,N_2)),$ where ${\mathcal {Q}}_1 \cup _\mathfrak {\epsilon } {\mathcal {Q}}_2=({\mathfrak {Y}},{\mathcal {B}}_1\cup {\mathcal {B}}_2,\max (N_1,N_2)),$ and $\zeta (b_t)$ is given by:

$$\zeta (b_t)=\left\{ \begin{array}{ll} {\mathcal {H}}_1(b_t), &{} \hbox {if } b_t\in {\mathcal {B}}_1-{\mathcal {B}}_2, \\ {\mathcal {H}}_2(b_t), &{} \hbox {if }b_t\in {\mathcal {B}}_2-{\mathcal {B}}_1, \\ \langle (u_g,r_{a_{gt}}),y,z\rangle , &{} \hbox {such that }r_{a_{gt}} =\max (r_{a_{gt}}^1,r_{a_{gt}}^2),\\ &{}{y=\max (s_{{\mathcal {C}}}(u_g,r_{a_{gt}}^1),s_{{\mathcal {D}}} (u_g,r_{a_{gt}}^2))e^{i\max (\omega _{{{\mathcal {C}}}}(u_g,r_{a_{gt}}^1), \omega _{{{\mathcal {D}}}}(u_g,r_{a_{gt}}^2))},}\\ &{}{z=\min (k_{{\mathcal {C}}}(u_g,r_{a_{gt}}^1),k_{{\mathcal {D}}} (u_g,r_{a_{gt}}^2))e^{i\min (\psi _{{{\mathcal {C}}}}(u_g,r_{a_{gt}}^1), \psi _{{{\mathcal {D}}}}(u_g,r_{a_{gt}}^2))},}\\ &{}\hbox {where }\langle (u_g,r_{a_{gt}}^1),s_{{\mathcal {C}}}(u_g,r_{a_{gt}}^1) e^{i\omega _{{{\mathcal {C}}}}(u_g,r_{a_{gt}}^1)},k_{{\mathcal {C}}} (u_g,r_{a_{gt}}^1)e^{i\psi _{{{\mathcal {C}}}}(u_g,r_{a_{gt}}^1)} \rangle \in {\mathcal {B}}_1(b_{t})\\ &{}\hbox {and }\langle (u_g,r_{a_{gt}}^2),s_{{\mathcal {D}}}(u_g,r_{a_{gt}}^2) e^{i\omega _{{{\mathcal {D}}}}(u_g,r_{a_{gt}}^2)},k_{{\mathcal {D}}} (u_g,r_{a_{gt}}^2)e^{i\psi _{{{\mathcal {D}}}}(u_g,r_{a_{gt}}^2)}\rangle \in {\mathcal {B}}_2(b_{t}),\\ &{}{\mathcal {C}}\hbox { and }{\mathcal {D}} \hbox { are CFFSs on } {\mathcal {F}}_{1}(b_t) \hbox { and }{\mathcal {F}}_{2}(b_t), \hbox { respectively.} \end{array} \right.$$

### Example 3.9

The extended union $(\zeta ,{\mathcal {Q}}_1 ~\cup _\mathfrak {\epsilon }~ {\mathcal {Q}}_2 ,5)$ of $({\mathcal {H}}_1,{\mathcal {Q}}_1,5)$ (see Table 15) and $({\mathcal {H}}_2,{\mathcal {Q}}_2,4)$ (see Table 16) is represented by Table 20.

**Table 20:** Tabulated form of $({\mathcal {H}}_1,{\mathcal {Q}}_1,5)~\cup _{\mathfrak {E}}~({\mathcal {H}}_2,{\mathcal {Q}}_2,4)$ given in Example 3.9

$(\zeta ,{\mathcal {Q}}_1\cup _\mathfrak {\epsilon }{\mathcal {Q}}_2 ,5)$	$b_{1}$	$b_{2}$	$b_{3}$	$\upsilon$
$x_{1}$	$\langle 4,(0.92e^{i1.88\pi },0.08e^{i0.36\pi })\rangle$	$\langle 3,(0.77e^{i1.58\pi },0.29e^{i0.67\pi })\rangle$	$\langle 2,(0.61e^{i1.14\pi },0.42e^{i1.29\pi })\rangle$	$\langle 3,(0.88e^{i1.76\pi },0.05e^{i0.19\pi })\rangle$
$x_{2}$	$\langle 3,(0.73e^{i1.46\pi },0.27e^{i0.78\pi })\rangle$	$\langle 4,(0.97e^{i1.96\pi },0.15e^{i0.29\pi })\rangle$	$\langle 1,(0.37e^{i0.63\pi },0.59e^{i1.54\pi })\rangle$	$\langle 2,(0.69e^{i0.97\pi },0.38e^{i0.65\pi })\rangle$
$x_{3}$	$\langle 4,(0.89e^{i1.76\pi },0.09e^{i0.36\pi })\rangle$	$\langle 2,(0.49e^{i1.27\pi },0.57e^{i1.18\pi })\rangle$	$\langle 0,(0.05e^{i0.32\pi },0.92e^{i1.94\pi })\rangle$	$\langle 1,(0.29e^{i0.72\pi },0.58e^{i1.33\pi })\rangle$
$x_{4}$	$\langle 3,(0.93e^{i1.88\pi },0.15e^{i0.25\pi })\rangle$	$\langle 1,(0.38e^{i0.67\pi },0.76e^{i1.29\pi })\rangle$	$\langle 2,(0.55e^{i0.95\pi },0.61e^{i0.85\pi })\rangle$	$\langle 0,(0.12e^{i0.37\pi },0.91e^{i1.87\pi })\rangle$

### Definition 3.16

Suppose that *U* be a universe of discourse and $({\mathcal {H}},{\mathcal {Q}},N)$ be a CFFNS${_f}$S over non-empty set *U*,  where ${\mathcal {Q}}=({\mathcal {F}},{\mathcal {B}},N)$ is an $\hbox {NS}_f$S on *U*. Let $0<L<N$ be a threshold. A CFFS${_f}$S related with $({\mathcal {H}},{\mathcal {Q}},N)$ and *L*,  denoted by $({\mathcal {H}}^L,{\mathcal {B}}),$ is given as follows:

$${\mathcal {H}}^L(b_t)=\left\{ \begin{array}{ll} (s(u_g,r_{a_{gt}})e^{i\omega (u_g,r_{a_{gt}})},k(u_g,r_{a_{gt}}) e^{i\psi (u_g,r_{a_{gt}})}), &{} \hbox {if }{\mathcal {H}}(b_t)=\langle (u_g,r_{a_{gt}}),s(u_g,r_{a_{gt}}) e^{i\omega (u_g,r_{a_{gt}})},k(u_g,r_{a_{gt}}) \\ e^{i\psi (u_g,r_{a_{gt}})}\rangle , &{}\hbox { and }r_{a_{gt}}\ge L,\\ (0.0e^{i0.0\pi },1.0e^{i2.0\pi }) &{} \hbox {otherwise.} \end{array} \right.$$

Particularly, $({\mathcal {H}}^1,{\mathcal {B}})$ is called the bottom $\hbox {CFFS}_f$S linked with $({\mathcal {H}},{\mathcal {Q}},N)$ and $({\mathcal {H}}^{N-1},{\mathcal {B}})$ is called top $\hbox {CFFS}_f$S associated with $({\mathcal {H}},{\mathcal {Q}},N).$

### Definition 3.17

Let $0<L<N$ and $\partial \in [0,1]$ be two thresholds. The $\hbox {S}_f$S over *U* associated with $({\mathcal {H}},{\mathcal {Q}},N)$ and $(L,\partial )$ is $({\mathfrak {f}}^{(L,\partial )},{\mathcal {B}})$ given by: for each $b_t\in {\mathcal {B}},{\mathfrak {f}}^{(L,\partial )}(b_t)=\{u_g \in U: {\mathcal {H}}^L(b_t)>\partial \}.$

### Example 3.10

Consider the CFF5$\hbox {S}_f$S in Example 3.1, represented by Table 6. From Definition 3.16, we can find the associated $\hbox {CFFS}_f$Ss with CFF5$\hbox {S}_f$S. Let $0<L<5$ be threshold. Then the possible $\hbox {CFFS}_f$S associated with thresholds 1, 2, 3 and 4 are shown by Tables 21–24.

**Table 21:** $\hbox {CFFS}_f$S associated with CFF5$\hbox {S}_f$S and threshold 1

$({\mathcal {H}}^1, {\mathcal {B}})$	$b_{1}$	$b_{2}$	$b_{3}$
$x_{1}$	$(0.5e^{i0.9\pi },0.6e^{i1.2\pi })$	$(0.7e^{i1.5\pi },0.3e^{i0.5\pi })$	$(0.3e^{i0.6\pi },0.8e^{i1.6\pi })$
$x_{2}$	$(0.3e^{i0.5\pi },0.7e^{i1.5\pi })$	$(0.8e^{i1.6\pi },0.4e^{i0.6\pi })$	$(0.6e^{i0.9\pi },0.5e^{i1.2\pi })$
$x_{3}$	$(0.7e^{i1.4\pi },0.3e^{i0.5\pi })$	$(0.9e^{i1.8\pi },0.1e^{i0.3\pi })$	$(0.0e^{i0.0\pi },1.0e^{i2.0\pi })$
$x_{4}$	$(0.0e^{i0.0\pi },1.0e^{i2.0\pi })$	$(0.5e^{i0.9\pi },0.5e^{i1.0\pi })$	$(0.9e^{i1.9\pi },0.2e^{i0.3\pi })$

**Table 22:** $\hbox {CFFS}_f$S associated with CFF5$\hbox {S}_f$S and threshold 2

$({\mathcal {H}}^2, {\mathcal {B}})$	$b_{1}$	$b_{2}$	$b_{3}$
$x_{1}$	$(0.5e^{i0.9\pi },0.6e^{i1.2\pi })$	$(0.7e^{i1.5\pi },0.3e^{i0.5\pi })$	$(0.0e^{i0.0\pi },1.0e^{i2.0\pi })$
$x_{2}$	$(0.0e^{i0.0\pi },1.0e^{i2.0\pi })$	$(0.8e^{i1.6\pi },0.4e^{i0.6\pi })$	$(0.6e^{i0.9\pi },0.5e^{i1.2\pi })$
$x_{3}$	$(0.7e^{i1.4\pi },0.3e^{i0.5\pi })$	$(0.9e^{i1.8\pi },0.1e^{i0.3\pi })$	$(0.0e^{i0.0\pi },1.0e^{i2.0\pi })$
$x_{4}$	$(0.0e^{i0.0\pi },1.0e^{i2.0\pi })$	$(0.5e^{i0.9\pi },0.5e^{i1.0\pi })$	$(0.9e^{i1.9\pi },0.2e^{i0.3\pi })$

**Table 23:** $\hbox {CFFS}_f$S associated with CFF5$\hbox {S}_f$S and threshold 3

$({\mathcal {H}}^3, {\mathcal {B}})$	$b_{1}$	$b_{2}$	$b_{3}$
$x_{1}$	$(0.0e^{i0.0\pi },1.0e^{i2.0\pi })$	$(0.7e^{i1.5\pi },0.3e^{i0.5\pi })$	$(0.0e^{i0.0\pi },1.0e^{i2.0\pi })$
$x_{2}$	$(0.0e^{i0.0\pi },1.0e^{i2.0\pi })$	$(0.8e^{i1.6\pi },0.4e^{i0.6\pi })$	$(0.0e^{i0.0\pi },1.0e^{i2.0\pi })$
$x_{3}$	$(0.7e^{i1.4\pi },0.3e^{i0.5\pi })$	$(0.9e^{i1.8\pi },0.1e^{i0.3\pi })$	$(0.0e^{i0.0\pi },1.0e^{i2.0\pi })$
$x_{4}$	$(0.0e^{i0.0\pi },1.0e^{i2.0\pi })$	$(0.0e^{i0.0\pi },1.0e^{i2.0\pi })$	$(0.9e^{i1.9\pi },0.2e^{i0.3\pi })$

**Table 24:** $\hbox {CFFS}_f$S associated with CFF5$\hbox {S}_f$S and threshold 4

$({\mathcal {H}}^4, {\mathcal {B}})$	$b_{1}$	$b_{2}$	$b_{3}$
$x_{1}$	$(0.0e^{i0.0\pi },1.0e^{i2.0\pi })$	$(0.0e^{i0.0\pi },1.0e^{i2.0\pi })$	$(0.0e^{i0.0\pi },1.0e^{i2.0\pi })$
$x_{2}$	$(0.0e^{i0.0\pi },1.0e^{i2.0\pi })$	$(0.0e^{i0.0\pi },1.0e^{i2.0\pi })$	$(0.0e^{i0.0\pi },1.0e^{i2.0\pi })$
$x_{3}$	$(0.0e^{i0.0\pi },1.0e^{i2.0\pi })$	$(0.9e^{i1.8\pi },0.1e^{i0.3\pi })$	$(0.0e^{i0.0\pi },1.0e^{i2.0\pi })$
$x_{4}$	$(0.0e^{i0.0\pi },1.0e^{i2.0\pi })$	$(0.0e^{i0.0\pi },1.0e^{i2.0\pi })$	$(0.9e^{i1.9\pi },0.2e^{i0.3\pi })$

## Operations

### Definition 4.1

Let $\alpha _{a_{1t}}=\langle r_{a_{1t}},(s_{a_{1t}}e^{i\omega _{a_{1t}}},k_{a_{1t}}e^{i\psi _{a_{1t}}}) \rangle ~ (t = 1,2)$ and $\alpha =\langle r,(s e^{i\omega },k e^{i\psi })\rangle$ be three $\hbox {CFFNS}_f$Ns over *U* and $\xi > 0.$ Then, some operations for $\hbox {CFFNS}_f$Ns are:

1. $$\alpha _{a_{11}} \cup \alpha _{a_{12}}=\langle \max (r_{a_{11}},r_{a_{12}}),(\max (s_{a_{11}},s_{a_{12}})e^{i\max (\omega _{a_{11}},\omega _{a_{12}})},\min (k_{a_{11}},k_{a_{12}})e^{i\min (\psi _{a_{11}},\psi _{a_{12}})})\rangle$$
2. $$\alpha _{a_{11}} \cap \alpha _{a_{12}}=\langle \min (r_{a_{11}},r_{a_{12}}),(\min (s_{a_{11}},s_{a_{12}})e^{i\min (\omega _{a_{11}},\omega _{a_{12}})},\max (k_{a_{11}},k_{a_{12}})e^{i\max (\psi _{a_{11}},\psi _{a_{12}})})\rangle$$
3. $$\alpha ^c=\langle r,(k e^{i\psi },s e^{i\omega })\rangle$$
4. $$\alpha _{a_{11}} \bigoplus \alpha _{a_{12}}=\langle \max (r_{a_{11}},r_{a_{12}}),(\root 3 \of {(s_{a_{11}})^3+(s_{a_{12}})^3-(s_{a_{11}})^3(s_{a_{12}})^3}e^{i2\pi \root 3 \of {(\frac{\omega _{a_{11}}}{2\pi })^3+(\frac{\omega _{a_{12}}}{2\pi })^3-(\frac{\omega _{a_{11}}}{2\pi })^3(\frac{\omega _{a_{12}}}{2\pi })^3}},k_{a_{11}}k_{a_{12}}e^{i2\pi ({\frac{\psi _{a_{11}}}{2\pi }})({\frac{\psi _{a_{12}}}{2\pi }})})\rangle$$
5. $$\alpha _{a_{11}} \bigotimes \alpha _{a_{12}}=\langle \min (r_{a_{11}},r_{a_{12}}),(s_{a_{11}}s_{a_{12}}e^{i2\pi (\frac{\omega _{a_{11}}}{2\pi })(\frac{\omega _{a_{12}}}{2\pi })},\root 3 \of {(k_{a_{11}})^3+(k_{a_{12}})^3-(k_{a_{11}})^3(k_{a_{12}})^3}~e^{i2\pi \root 3 \of {(\frac{\psi _{a_{11}}}{2\pi })^3+(\frac{\psi _{a_{12}}}{2\pi })^3-(\frac{\psi _{a_{11}}}{2\pi })^3(\frac{\psi _{a_{12}}}{2\pi })^3}})\rangle$$
6. $$\xi \alpha =\big \langle r,\big (\root 3 \of {1-(1-s^3)^\xi } e^{i2\pi \root 3 \of {1-(1-(\frac{\omega }{2\pi })^3)^\xi }},k^\xi e^{i2\pi (\frac{\psi }{2\pi })^\xi }\big )\big \rangle$$
7. $$\alpha ^\xi =\big \langle r,\big (s^\xi e^{i2\pi {(\frac{\omega }{2\pi })^\xi }},\root 3 \of {1-(1-k^3)^\xi } e^{i2\pi \root 3 \of {1-(1-(\frac{\psi }{2\pi })^3)^\xi }}\big )\big \rangle$$

### Definition 4.2

Let $\alpha _{a_{1t}}=\langle r_{a_{1t}},(s_{a_{1t}}e^{i\omega _{a_{1t}}},k_{a_{1t}}e^{i\psi _{a_{1t}}})\rangle ~ (t = 1,2)$ and $\alpha =\langle r,(s e^{i\omega },k e^{i\psi })\rangle$ be three $\hbox {CFFNS}_f$Ns over *U* and $\xi > 0.$ Then, Yager operations for $\hbox {CFFNS}_f$Ns are:

1. $$\alpha _{a_{11}} \bigoplus \alpha _{a_{12}}=\langle \max (r_{a_{11}},r_{a_{12}}), (\root 3 \of {\min (1,(s_{a_{11}}^{3\varphi }+s_{a_{12}}^{3\varphi })^{\frac{1}{\varphi }})}~e^{i2\pi \root 3 \of {\min (1,((\frac{\omega _{a_{11}}}{2\pi })^{3\varphi }+(\frac{\omega _{a_{12}}}{2\pi })^{3\varphi })^{\frac{1}{\varphi }})}}, \root 3 \of {1-\min (1,((1-k_{a_{11}}^3)^\varphi +(1-k_{a_{12}}^3)^\varphi )^{\frac{1}{\varphi }})}~e^{i2\pi \root 3 \of {1-\min (1,((1-(\frac{\psi _{a_{11}}}{2\pi })^3)^\varphi +(1-(\frac{\psi _{a_{12}}}{2\pi })^3)^\varphi )^{\frac{1}{\varphi }})}})\rangle$$
2. $$\alpha _{a_{11}} \bigotimes \alpha _{a_{12}}=\langle \min (r_{a_{11}},r_{a_{12}}),(\root 3 \of {1-\min (1,((1-s_{a_{11}}^3)^\varphi +(1-s_{a_{12}}^3)^\varphi )^{\frac{1}{\varphi }})} e^{i2\pi \root 3 \of {1-\min (1,((1-(\frac{\omega _{a_{11}}}{2\pi })^3)^\varphi +(1-(\frac{\omega _{a_{12}}}{2\pi })^3)^\varphi )^{\frac{1}{\varphi }})}}, \root 3 \of {\min (1,(k_{a_{11}}^{3\varphi }+k_{a_{12}}^{3\varphi })^{\frac{1}{\varphi }})}e^{i2\pi \root 3 \of {\min (1,((\frac{\psi _{a_{11}}}{2\pi })^{3\varphi }+(\frac{\psi _{a_{12}}}{2\pi })^{3\varphi })^{\frac{1}{\varphi }})}})\rangle$$
3. $$\xi \alpha =\big \langle r, \big (\root 3 \of {\min (1,(\xi s^{3\varphi })^{\frac{1}{\varphi }})}e^{i2\pi \root 3 \of {\min (1,(\xi (\frac{\omega }{2\pi })^{3\varphi })^{\frac{1}{\varphi }})}}, \root 3 \of {1-\min (1,(\xi (1-k^3)^\varphi )^{\frac{1}{\varphi }})}e^{i2\pi \root 3 \of {1-\min (1,(\xi (1-(\frac{\psi }{2\pi })^3)^\varphi )^{\frac{1}{\varphi }})}}\big )\big \rangle$$
4. $$\alpha ^\xi =\big \langle r, \big (\root 3 \of {1-\min (1,(\xi (1-s^3)^\varphi )^{\frac{1}{\varphi }})}e^{i2\pi \root 3 \of {1-\min (1,(\xi (1-(\frac{\omega }{2\pi })^3)^\varphi )^{\frac{1}{\varphi }})}},\root 3 \of {\min (1,(\xi k^{3\varphi })^{\frac{1}{\varphi }})}e^{i2\pi \root 3 \of {\min (1,(\xi (\frac{\psi }{2\pi })^{3\varphi })^{\frac{1}{\varphi }})}})\rangle$$

- where $\varphi$ is the parameter and $\varphi \in (0,\infty ).$

### Remark 2

We have used the name “Yager operations” because these operations are derived from the use of the theoretical foundations of Yager s-norm and t-norm in the $\hbox {CFFNS}_f$S environment. These operations carry the accuracy feature and aggregation skills of the Yager norm for flexible model of $\hbox {CFFNS}_f$Ns.

### Example 4.1

Let $\alpha _1 = \langle 3,(0.8e^{i1.6\pi },0.4e^{i0.6\pi })\rangle$ and $\alpha _2=\langle 2,(0.6e^{i0.9\pi },0.5e^{i1.2\pi })\rangle$ be two $\hbox {CFFNS}_f$Ns, and then by using Definition 4.2 for $\xi =2$, $\varphi =4$ they are:

$$\begin{aligned} \alpha _1 \bigoplus \alpha _2= & {} \bigg \langle \max (3,2), \bigg (\root 3 \of {\min (1,(0.8^{12}+0.6^{12})^{\frac{1}{4}})}~e^{i2\pi \root 3 \of {\min (1,((\frac{1.6\pi }{2\pi })^{12}+(\frac{0.9\pi }{2\pi })^{12})^{\frac{1}{4}})}},\\&\root 3 \of {1-\min (1,((1-0.4^3)^4+(1-0.5^3)^4)^{\frac{1}{4}})}~e^{i2\pi \root 3 \of {1-\min (1,((1-(\frac{0.6\pi }{2\pi })^3)^4+(1-(\frac{1.2\pi }{2\pi })^3)^4)^{\frac{1}{4}})}}\bigg )\bigg \rangle \\= & {} \langle 3,(0.8e^{i1.6\pi },0e^{i0})\rangle \\ \alpha _1 \bigotimes \alpha _2= & {} \bigg \langle \min (3,2),\bigg (\root 3 \of {1-\min (1,((1-0.8^3)^4+(1-0.6^3)^4)^{\frac{1}{4}})}~e^{i2\pi \root 3 \of {1-\min (1,((1-(\frac{1.6\pi }{2\pi })^3)^4+(1-(\frac{0.9\pi }{2\pi })^3)^4)^{\frac{1}{4}})}},\\&\root 3 \of {\min (1,(0.4^{12}+0.5^{12})^{\frac{1}{4}})}~e^{i2\pi \root 3 \of {\min (1,((\frac{0.6\pi }{2\pi })^{12}+(\frac{1.2\pi }{2\pi })^{12})^{\frac{1}{4}})}}\bigg )\bigg \rangle \\= & {} \langle 2,(0.57e^{i0.83\pi },0.5e^{i1.2\pi })\rangle \\ 2\alpha _1= & {} \big \langle 3, \big (\root 3 \of {\min (1,(2(0.8)^{12})^{\frac{1}{4}})}~e^{i2\pi \root 3 \of {\min (1,(2(\frac{1.6\pi }{2\pi })^{12})^{\frac{1}{4}})}}, \root 3 \of {1-\min (1,(2(1-0.4^3)^4)^{\frac{1}{4}})}\\&e^{i2\pi \root 3 \of {1-\min (1,(2(1-(\frac{0.6\pi }{2\pi })^3)^4)^{\frac{1}{4}})}}\big )\big \rangle \\= & {} \langle 3,(0.85e^{i1.7\pi },0e^{i0})\rangle \\ \alpha _1^2= & {} \big \langle 3, \big (\root 3 \of {1-\min (1,(2(1-0.8^3)^4)^{\frac{1}{4}})}~e^{i2\pi \root 3 \of {1-\min (1,(2(1-(\frac{1.6\pi }{2\pi })^3)^4)^{\frac{1}{4}})}},\root 3 \of {\min (1,(2(0.4)^{12})^{\frac{1}{4}})}\\&e^{i2\pi \root 3 \of {\min (1,(2(\frac{0.6\pi }{2\pi })^{12})^{\frac{1}{4}})}})\rangle \\= & {} \langle 3,(0.75e^{i1.5\pi },0.42e^{i0.64\pi })\rangle \end{aligned}$$

## Algorithms and applications

In this section, we clarify the decision-making (DM) process for the constructed model. Firstly, we construct the procedures as shown in Algorithms 1–3 for problems that are described by $\hbox {CFFNS}_f$Ss. Then, we apply them to real circumstances to get the particular results. 
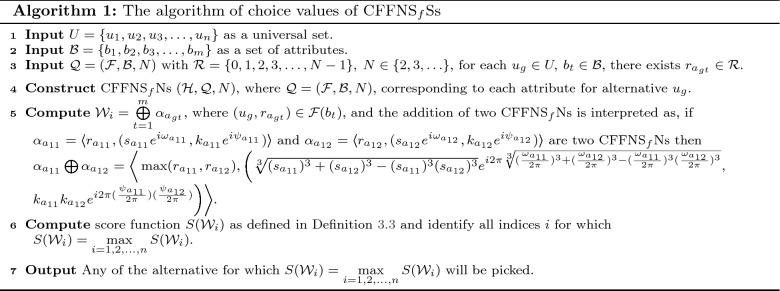

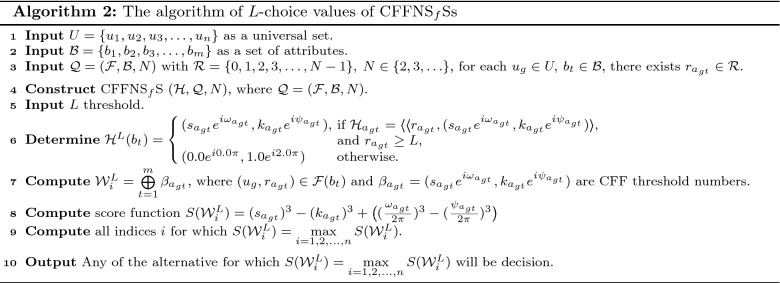

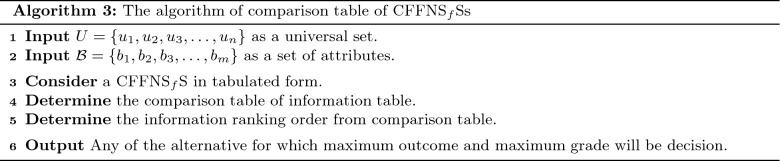


### Selection of buy new car

Selection of a car is a difficult task for an auto broker. Productive selection is possible only when there is an essential matching. By choosing the best car, the auto broker will get quality performance. In Example 3.1, different $\hbox {CFFNS}_f$Ns for the cars have been defined on the basis of their qualities, by the auto broker. Tabulated form of CFF5S $_f$S is represented by Table 25.

**Table 25:** Tabular form of the CFF5$\hbox {S}_f$S $({\mathcal {H}}, {\mathcal {Q}}, 5)$

$({\mathcal {H}}, {\mathcal {Q}}, 5)$	$b_{1}$	$b_{2}$	$b_{3}$
$x_{1}$	$\langle 2,(0.5e^{i0.9\pi },0.6e^{i1.2\pi })\rangle$	$\langle 3,(0.7e^{i1.5\pi },0.3e^{i0.5\pi })\rangle$	$\langle 1,(0.3e^{i0.6\pi },0.8e^{i1.6\pi })\rangle$
$x_{2}$	$\langle 1,(0.3e^{i0.5\pi },0.7e^{i1.5\pi })\rangle$	$\langle 3,(0.8e^{i1.6\pi },0.4e^{i0.6\pi })\rangle$	$\langle 2,(0.6e^{i0.9\pi },0.5e^{i1.2\pi })\rangle$
$x_{3}$	$\langle 3,(0.7e^{i1.4\pi },0.3e^{i0.5\pi })\rangle$	$\langle 4,(0.9e^{i1.8\pi },0.1e^{i0.3\pi })\rangle$	$\langle 0,(0.1e^{i0.2\pi },0.9e^{i1.9\pi })\rangle$
$x_{4}$	$\langle 0,(0.1e^{i0.2\pi },0.9e^{i1.8\pi })\rangle$	$\langle 2,(0.5e^{i0.9\pi },0.5e^{i1.0\pi })\rangle$	$\langle 4,(0.9e^{i1.9\pi },0.2e^{i0.3\pi })\rangle$

$\bullet$ **Choice value (CV) of CFF5**$\hbox {S}_f$ **S**

We can calculate the CV of CFF5$\hbox {S}_f$S of the car’s selection by using Algorithm 1 and calculated results are given in Table 26, where

$$\begin{aligned} S({\mathcal {W}}_i)=(\frac{r_{a_{gt}}}{N-1})^3+(s_{a_{gt}})^3 -(k_{a_{gt}})^3+\big ((\frac{\omega _{a_{gt}}}{2\pi })^3 -(\frac{\psi _{a_{gt}}}{2\pi })^3\big ). \end{aligned}$$

**Table 26:** Tabular form of CV of CFF5$\hbox {S}_f$S $({\mathcal {H}}, {\mathcal {Q}}, 5)$

$({\mathcal {H}}, {\mathcal {Q}}, 5)$	$b_{1}$	$b_{2}$	$b_{3}$	${\mathcal {W}}_i$	$S({\mathcal {W}}_i)$
$x_{1}$	$\langle 2,(0.5e^{i0.9\pi },0.6e^{i1.2\pi })\rangle$	$\langle 3,(0.7e^{i1.5\pi },0.3e^{i0.5\pi })\rangle$	$\langle 1,(0.3e^{i0.6\pi },0.8e^{i1.6\pi })\rangle$	$\langle 3, (0.76e^{i1.58\pi },0.14e^{i0.24\pi })\rangle$	1.35
$x_{2}$	$\langle 1,(0.3e^{i0.5\pi },0.7e^{i1.5\pi })\rangle$	$\langle 3,(0.8e^{i1.6\pi },0.4e^{i0.6\pi })\rangle$	$\langle 2,(0.6e^{i0.9\pi },0.5e^{i1.2\pi })\rangle$	$\langle 3,(0.86e^{i1.65\pi },0.14e^{i0.27\pi }) \rangle$	1.61
$x_{3}$	$\langle 3,(0.7e^{i1.4\pi },0.3e^{i0.5\pi })\rangle$	$\langle 4,(0.9e^{i1.8\pi },0.1e^{i0.3\pi })\rangle$	$\langle 0,(0.1e^{i0.2\pi },0.9e^{i1.9\pi })\rangle$	$\langle 4,(0.94e^{i1.87\pi },0.03e^{i0.07\pi }) \rangle$	2.65
$x_{4}$	$\langle 0,(0.1e^{i0.2\pi },0.9e^{i1.8\pi })\rangle$	$\langle 2,(0.5e^{i0.9\pi },0.5e^{i1.0\pi })\rangle$	$\langle 4,(0.9e^{i1.9\pi },0.2e^{i0.3\pi })\rangle$	$\langle 4,(0.91e^{i1.91\pi },0.09e^{i0.14\pi }) \rangle$	2.62

From Table 26, it is concluded that according to $S({\mathcal {W}}_i)$ values, $x_3>x_4>x_2>x_1$ and hence $x_3$ has maximum value. So, the auto broker will choose the car $x_3.$

$\bullet$ *L***-Choice value (L-CV) of** **CFF5**$\hbox {S}_f$**S**

Now, we will choose the threshold *L* and will calculate the CV by using Algorithm 2, where

$$\begin{aligned} S({\mathcal {W}}_i^L)=(s_{a_{gt}})^3-(k_{a_{gt}})^3 +\big ((\frac{\omega _{a_{gt}}}{2\pi })^3-(\frac{\psi _{a_{gt}}}{2\pi })^3\big ). \end{aligned}$$

The result is shown by Table 27.

**Table 27:** Tabular form of 2-CV of the CFF5$\hbox {S}_f$S $({\mathcal {H}}, {\mathcal {Q}}, 5)$

$({\mathcal {H}}^2, {\mathcal {B}})$	$b_{1}$	$b_{2}$	$b_{3}$	${\mathcal {W}}_i$	$S({\mathcal {W}}_i^L)$
$x_{1}$	$(0.5e^{i0.9\pi },0.6e^{i1.2\pi })$	$(0.7e^{i1.5\pi },0.3e^{i0.5\pi })$	$(0.0e^{i0.0\pi },1.0e^{i2.0\pi })$	$(0.75e^{i1.56\pi },0.18e^{i0.30\pi })$	0.89
$x_{2}$	$(0.0e^{i0.0\pi },1.0e^{i2.0\pi })$	$(0.8e^{i1.6\pi },0.4e^{i0.6\pi })$	$(0.6e^{i0.9\pi },0.5e^{i1.2\pi })$	$(0.85e^{i1.65\pi },0.20e^{i0.36\pi })$	1.16
$x_{3}$	$(0.7e^{i1.4\pi },0.3e^{i0.5\pi })$	$(0.9e^{i1.8\pi },0.1e^{i0.3\pi })$	$(0.0e^{i0.0\pi },1.0e^{i2.0\pi })$	$(0.94e^{i1.87\pi },0.03e^{i0.08\pi })$	1.65
$x_{4}$	$(0.0e^{i0.0\pi },1.0e^{i2.0\pi })$	$(0.5e^{i0.9\pi },0.5e^{i1.0\pi })$	$(0.9e^{i1.9\pi },0.2e^{i0.3\pi })$	$(0.91e^{i1.91\pi },0.10e^{i0.15\pi })$	1.62

In Table 27, we took $L=2$ for DM and get the 2-CV of CFF5$\hbox {S}_f$S. It can observe from Table 27 that the car $x_3$ has highest output value. So, $x_3$ will be selected by the auto broker.

$\bullet$ **Comparison table of** **CFF5**$\hbox {S}_f$**S**

*Comparison table* is a square table in which rows and columns are represented by the name of objects of universe such as $u_1, u_2, u_3, \ldots , u_n$ and $q_{gt}=$ the $\hbox {CFFNS}_f$ values of the attributes for which the value of score function of $u_d \ge u_j.$

Membership and non-membership values of Table 25 are given in tabular form in Table 28.

**Table 28:** Tabular form of the membership and non-membership values of CFF5$\hbox {S}_f$S $({\mathcal {H}}, {\mathcal {Q}}, 5)$

$({\mathcal {H}}, {\mathcal {Q}}, 5)$	$b_{1}$	$b_{2}$	$b_{3}$
$x_{1}$	$(0.5e^{i0.9\pi },0.6e^{i1.2\pi })$	$(0.7e^{i1.5\pi },0.3e^{i0.5\pi })$	$(0.3e^{i0.6\pi },0.8e^{i1.6\pi })$
$x_{2}$	$(0.3e^{i0.5\pi },0.7e^{i1.5\pi })$	$(0.8e^{i1.6\pi },0.4e^{i0.6\pi })$	$(0.6e^{i0.9\pi },0.5e^{i1.2\pi })$
$x_{3}$	$(0.7e^{i1.4\pi },0.3e^{i0.5\pi })$	$(0.9e^{i1.8\pi },0.1e^{i0.3\pi })$	$(0.1e^{i0.2\pi },0.9e^{i1.9\pi })$
$x_{4}$	$(0.1e^{i0.2\pi },0.9e^{i1.8\pi })$	$(0.5e^{i0.9\pi },0.5e^{i1.0\pi })$	$(0.9e^{i1.9\pi },0.2e^{i0.3\pi })$

The comparison table of Table 28 is given by Table 29.

**Table 29:** Comparison table of CFF5$\hbox {S}_f$S $({\mathcal {H}}, {\mathcal {Q}}, 5)$

	$x_{1}$	$x_{2}$	$x_{3}$	$x_{4}$
$x_{1}$	3	1	1	2
$x_{2}$	2	3	1	2
$x_{3}$	2	2	3	2
$x_{4}$	1	1	1	3

The result will be derived by subtracting the row and column sum of Table 29.

**Table 30:** Ranking Table

	Grade sum $(G_s)$	Row sum $(R_s)$	Column sum $(C_s)$	Final Rank $(R_s-C_s)$
$x_{1}$	6	7	8	$-1$
$x_{2}$	6	8	7	1
$x_{3}$	7	9	6	3
$x_{4}$	6	6	9	$-3$

From Table 30, it is concluded that the highest rank and grade sum is 3 and 7, respectively, which is obtained by $x_3.$ So, $x_3$ car is selected by the auto broker.

### Selection of the best cellular telecommunication company in Pakistan

Since due to COVID-19, everything has shifted to the online mode. So, the usage of the internet has increased. Due to that, in market, the competition among different network provider companies has been tough day by day. All companies are presenting different internet packages according to the needs of the customers. So, it’s a difficult task for customers to choose the sim card of the best telecommunication company. Suppose that a student, in Pakistan, decides to purchase a new sim card to attend online classes. Before buying the sim, the student has collected some relevant rating based information related to the internet packages and internet speed about five different network companies such as Zong, Ufone, Telenor, Jazz, and Warid. Each network company has different prices of internet packages depending on time as well. Let $Y=\{y_1= \mathrm{zong},~y_2=\mathrm{SCOM},~y_3=\mathrm{telenor},~y_4={jazz},~ y_5=\mathrm{ufone}\}$ be a universal set and ${\mathcal {O}}=\{o_1 = \mathrm{3G/4G~speed}, ~o_2 = \mathrm{Packages~time~duration}, ~o_3 = \mathrm{Monthly~cost}, ~o_4={Signal~strength} \}\subseteq {\mathcal {A}}$ be a set of attributes, which are used to assign grades to network companies. The ratings are on the basis of internet packages and speed provided in last year and users reviews. It may be noted that the ranking of alternatives with respect to parameters may get affected and altered if the time and location is different for a particular network company. The initial survey is organized in Table 31, where:

- five $\blacklozenge$ represents ‘outstanding’,
- four $\blacklozenge$ represents ‘very good’,
- three $\blacklozenge$ represents ‘good’,
- two $\blacklozenge$ represents ‘average’,
- one $\blacklozenge$ represents ‘subpar’,
- $$\lozenge$$ represents ‘poor’.

The set of grades ${\mathcal {R}}=\{0,1,2,3,4,5\}$ can be easily associated with $\blacklozenge$ and $\lozenge$ as follows:

- 0 stands for $$`\lozenge$$’,
- 1 stands for $$`\blacklozenge$$’,
- 2 stands for $$`\blacklozenge \blacklozenge$$’,
- 3 stands for $$`\blacklozenge \blacklozenge \blacklozenge$$’,
- 4 stands for $$`\blacklozenge \blacklozenge \blacklozenge \blacklozenge$$’,
- 5 stands for $$`\blacklozenge \blacklozenge \blacklozenge \blacklozenge \blacklozenge$$’.

Based on the overall qualities of the network companies, the student gives evaluation scores to the sim cards which is shown as Table 31 and the tabular representation of its associated 6-soft set is given in Table 32.

**Table 31:** Information extracted from the related data

${Y}/{\mathcal {O}}$	$o_{1}$	$o_{2}$	$o_{3}$	$o_{4}$
$y_{1}$	$\blacklozenge \blacklozenge \blacklozenge \blacklozenge \blacklozenge$	$\blacklozenge \blacklozenge \blacklozenge \blacklozenge$	$\blacklozenge \blacklozenge \blacklozenge$	$\blacklozenge \blacklozenge \blacklozenge \blacklozenge \blacklozenge$
$y_{2}$	$\lozenge$	$\blacklozenge \blacklozenge$	$\blacklozenge \blacklozenge$	$\blacklozenge$
$y_{3}$	$\blacklozenge \blacklozenge \blacklozenge$	$\blacklozenge \blacklozenge \blacklozenge \blacklozenge$	$\blacklozenge \blacklozenge \blacklozenge$	$\blacklozenge \blacklozenge$
$y_{4}$	$\blacklozenge$	$\blacklozenge \blacklozenge$	$\blacklozenge \blacklozenge \blacklozenge$	$\blacklozenge \blacklozenge$
$y_{5}$	$\blacklozenge \blacklozenge$	$\blacklozenge$	$\lozenge$	$\blacklozenge \blacklozenge \blacklozenge \blacklozenge$

**Table 32:** Tabular form of the 6-soft set $({\mathcal {F}}, {\mathcal {B}}, 6)$

$({\mathcal {F}}, {\mathcal {B}}, 6)$	$o_{1}$	$o_{2}$	$o_{3}$	$o_{4}$
$y_{1}$	5	4	3	5
$y_{2}$	1	2	2	1
$y_{3}$	3	4	3	2
$y_{4}$	0	2	3	2
$y_{5}$	2	1	0	4

Although it is easy to extract the grade data in actual information, the data possess the fuzzy uncertainty characteristics. In order to address the ambiguity of data, we construct $\hbox {CFFNS}_f$S by using a certain grade. This evaluation of sim cards by students complies with the guidelines as follows:

$$\begin{aligned} -2.0\le & {} S(Y)< -1.4~~~ \mathrm{when~ grade~} 0,\\ -1.4\le & {} S(Y)< -0.5~~~ \mathrm{when ~grade~} 1,\\ -0.5\le & {} S(Y)<~~ 0.0~~~ \mathrm{when ~grade~} 2, \\ 0.0\le & {} S(Y)<~~0.5~~~ \mathrm{when ~grade~} 3, \\ 0.5\le & {} S(Y) <~~ 1.4~~~ \mathrm{when ~grade~} 4,\\ 1.4\le & {} S(Y) \le ~~ 2.0~~~ \mathrm{when ~grade~} 5. \end{aligned}$$

By Definition 3.1, the CFF6$\hbox {S}_f$S $({\mathcal {H}},{\mathcal {Q}},6)$ can be defined as follows:

$$\begin{aligned} h(o_1)= & {} \{\langle (y_1,5),0.95e^{i1.78\pi },0.05e^{i0.24\pi }\rangle ,\langle (y_2,1),0.32e^{i0.54\pi },0.89e^{i1.74\pi }\rangle ,\langle (y_3,3),0.48e^{i1.06\pi },0.17e^{i0.30\pi }\rangle ,\\&\langle (y_4,0),0.21e^{i0.04\pi },0.83e^{i1.94\pi }\rangle \},\langle (y_5,2),0.29e^{i0.84\pi },0.57e^{i1.16pi}\rangle \}, \\ h(o_2)= & {} \{\langle (y_1,4),0.76e^{i1.69\pi },0.39e^{i0.29\pi }\rangle ,\langle (y_2,2),0.24e^{i0.64\pi },0.43e^{i1.22\pi }\rangle ,\langle (y_3,4),0.7e^{i1.71\pi },0.32e^{i0.21\pi }\rangle ,\\&\langle (y_4,2),0.26e^{i0.56\pi },0.43e^{i1.21\pi }\rangle \}, \langle (y_5,1),0.34e^{i0.84\pi },0.95e^{i1.71\pi }\rangle \},\\ h(o_3)= & {} \{\langle (y_1,3),0.58e^{i1.38\pi },0.23e^{i0.52\pi }\rangle ,\langle (y_2,2),0.29e^{i0.54\pi },0.49e^{i0.98\pi }\rangle ,\langle (y_3,3),0.52e^{i1.44\pi },0.32e^{i0.72\pi }\rangle ,\\&\langle (y_4,3),0.69e^{i1.28\pi },0.45e^{i0.86\pi }\rangle \},\langle (y_5,0),0.07e^{i0.24\pi },0.99e^{i1.88\pi }\rangle \}, \\ h(o_4)= & {} \{\langle (y_1,5),0.91e^{i1.93\pi },0.02e^{i0.05\pi }\rangle ,\langle (y_2,1),0.43e^{i0.66\pi },0.85e^{i1.46\pi }\rangle ,\langle (y_3,2),0.44e^{i0.44\pi },0.35e^{i1.08\pi }\rangle ,\\&\langle (y_4,2),0.19e^{i0.38\pi },0.37e^{i1.06\pi }\rangle \},\langle (y_5,4),0.81e^{i1.53\pi },0.12e^{i0.34\pi }\rangle \}. \end{aligned}$$

The CFF6$\hbox {S}_f$S $({\mathcal {H}},{\mathcal {Q}},6)$ can be represented more clearly in tabular form shown as in Table 33.

**Table 33:** Tabular form of the CFF6$\hbox {S}_f$S $({\mathcal {H}}, {\mathcal {Q}}, 6)$

$({\mathcal {H}}, {\mathcal {Q}}, 6)$	$o_{1}$	$o_{2}$	$o_{3}$	$o_{4}$
$y_{1}$	$\langle 5,(0.95e^{i1.78\pi },0.05e^{i0.24\pi })\rangle$	$\langle 4,(0.76e^{i1.69\pi },0.39e^{i0.29\pi })\rangle$	$\langle 3,(0.58e^{i1.38\pi },0.23e^{i0.52\pi })\rangle$	$\langle 5,(0.91e^{i1.93\pi },0.02e^{i0.05\pi })\rangle$
$y_{2}$	$\langle 1,(0.32e^{i0.54\pi },0.89e^{i1.74\pi })\rangle$	$\langle 2,(0.24e^{i0.64\pi },0.43e^{i1.22\pi })\rangle$	$\langle 2,(0.29e^{i0.54\pi },0.49e^{i0.98\pi })\rangle$	$\langle 1,(0.43e^{i0.66\pi },0.85e^{i1.46\pi })\rangle$
$y_{3}$	$\langle 3,(0.48e^{i1.06\pi },0.17e^{i0.30\pi })\rangle$	$\langle 4,(0.70e^{i1.71\pi },0.32e^{i0.21\pi })\rangle$	$\langle 3,(0.52e^{i1.44\pi },0.32e^{i0.72\pi })\rangle$	$\langle 2,(0.44e^{i0.44\pi },0.35e^{i1.08\pi })\rangle$
$y_{4}$	$\langle 0,(0.21e^{i0.04\pi },0.83e^{i1.94\pi })\rangle$	$\langle 2,(0.26e^{i0.56\pi },0.43e^{i1.21\pi })\rangle$	$\langle 3,(0.69e^{i1.28\pi },0.45e^{i0.86\pi })\rangle$	$\langle 2,(0.19e^{i0.38\pi },0.37e^{i1.06\pi })\rangle$
$y_{5}$	$\langle 2,(0.29e^{i0.84\pi },0.57e^{i1.16\pi })\rangle$	$\langle 1,(0.34e^{i0.84\pi },0.95e^{i1.71\pi })\rangle$	$\langle 0,(0.07e^{i0.24\pi },0.99e^{i1.88\pi })\rangle$	$\langle 4,(0.81e^{i1.53\pi },0.12e^{i0.34\pi })\rangle$

$\bullet$ **Choice value (CV) of** **CFF6**$\hbox {S}_f$**S**

We can calculate the CV of CFF6$\hbox {S}_f$S of the sim card’s selection by using Algorithm 1 as given by Table 34, where

$$\begin{aligned} S({\mathcal {W}}_i)=(\frac{r_{a_{gt}}}{N-1})^3+(s_{a_{gt}})^3 -(k_{a_{gt}})^3+\big ((\frac{\omega _{a_{gt}}}{2\pi })^3 -(\frac{\psi _{a_{gt}}}{2\pi })^3\big ). \end{aligned}$$

**Table 34:** Tabular form of the CV of CFF6$\hbox {S}_f$S $({\mathcal {H}}, {\mathcal {Q}}, 6)$

$({\mathcal {H}}, {\mathcal {Q}}, 6)$	$o_{1}$	$o_{2}$	$o_{3}$	$o_{4}$	${\mathcal {W}}_i$	$S({\mathcal {W}}_i)$
$y_{1}$	$\langle 5,(0.95e^{i1.78\pi },0.05e^{i0.24\pi })\rangle$	$\langle 4,(0.76e^{i1.69\pi },0.39e^{i0.29\pi })\rangle$	$\langle 3,(0.58e^{i1.38\pi },0.23e^{i0.52\pi })\rangle$	$\langle 5,(0.91e^{i1.93\pi },0.02e^{i0.05\pi })\rangle$	$\langle 5,(0.99e^{i1.99\pi },8.97\times 10^{-5}e^{i2.26\times 10^{-4}\pi })\rangle$	2.96
$y_{2}$	$\langle 1,(0.32e^{i0.54\pi },0.89e^{i1.74\pi })\rangle$	$\langle 2,(0.24e^{i0.64\pi },0.43e^{i1.22\pi })\rangle$	$\langle 2,(0.29e^{i0.54\pi },0.49e^{i0.98\pi })\rangle$	$\langle 1,(0.43e^{i0.66\pi },0.85e^{i1.46\pi })\rangle$	$\langle 2,(0.52e^{i0.94\pi },0.16e^{i0.38\pi })\rangle$	0.30
$y_{3}$	$\langle 3,(0.48e^{i1.06\pi },0.17e^{i0.30\pi })\rangle$	$\langle 4,(0.70e^{i1.71\pi },0.32e^{i0.21\pi })\rangle$	$\langle 3,(0.52e^{i1.44\pi },0.32e^{i0.72\pi })\rangle$	$\langle 2,(0.44e^{i0.44\pi },0.35e^{i1.08\pi })\rangle$	$\langle 4,(0.82e^{i1.86\pi },6.09\times 10^{-3}e^{i6.12\times 10^{-3}\pi })\rangle$	1.87
$y_{4}$	$\langle 0,(0.21e^{i0.04\pi },0.83e^{i1.94\pi })\rangle$	$\langle 2,(0.26e^{i0.56\pi },0.43e^{i1.21\pi })\rangle$	$\langle 3,(0.69e^{i1.28\pi },0.45e^{i0.86\pi })\rangle$	$\langle 2,(0.19e^{i0.38\pi },0.37e^{i1.06\pi })\rangle$	$\langle 3,(0.70e^{i1.32\pi },0.06e^{i0.27\pi })\rangle$	0.84
$y_{5}$	$\langle 2,(0.29e^{i0.84\pi },0.57e^{i1.16\pi })\rangle$	$\langle 1,(0.34e^{i0.84\pi },0.95e^{i1.71\pi })\rangle$	$\langle 0,(0.07e^{i0.24\pi },0.99e^{i1.88\pi })\rangle$	$\langle 4,(0.81e^{i1.53\pi },0.12e^{i0.34\pi })\rangle$	$\langle 4,(0.82e^{i1.62\pi },0.06e^{i0.16\pi })\rangle$	1.59

From Table 34, it is concluded that according to $S({\mathcal {W}}_i),$ $y_1>y_3>y_5>y_4>y_2$ and hence $y_1$ has maximum value. So, the student will choose the sim card of zong.

$\bullet$ *L***-Choice value (L-CV) of CFF6**$\hbox {S}_f$**S**

Now, by using the second procedure as given by Algorithm 2, *L* value will be chosen and the results are given by Table 35, where

$$\begin{aligned} S({\mathcal {W}}_i^L)=(s_{a_{gt}})^3-(k_{a_{gt}})^3 +\big ((\frac{\omega _{a_{gt}}}{2\pi })^3-(\frac{\psi _{a_{gt}}}{2\pi })^3\big ). \end{aligned}$$

**Table 35:** Tabular form of the 3-CV of CFF6$\hbox {S}_f$S $({\mathcal {H}}, {\mathcal {Q}}, 6)$

$({\mathcal {H}}^3, {\mathcal {B}})$	$o_{1}$	$o_{2}$	$o_{3}$	$o_{4}$	${\mathcal {W}}_i^3$	$S({\mathcal {W}}_i^3)$
$y_{1}$	$(0.95e^{i1.78\pi },0.05e^{i0.24\pi })$	$(0.76e^{i1.69\pi },0.39e^{i0.29\pi })$	$(0.58e^{i1.38\pi },0.23e^{i0.52\pi })$	$(0.91e^{i1.93\pi },0.02e^{i0.05\pi })$	$(0.99e^{i1.99\pi },8.97\times 10^{-5}e^{i2.26\times 10^{-4}\pi })$	1.96
$y_{2}$	$(0.00e^{i0.00\pi },1.00e^{i2.00\pi })$	$(0.00e^{i0.00\pi },1.00e^{i2.00\pi })$	$(0.00e^{i0.00\pi },1.00e^{i2.00\pi })$	$(0.00e^{i0.00\pi },1.00e^{i2.00\pi })$	$(0.00e^{i0.00\pi },1.00e^{i2.00\pi })$	$-2.00$
$y_{3}$	$(0.48e^{i1.06\pi },0.17e^{i0.30\pi })$	$(0.70e^{i1.71\pi },0.32e^{i0.21\pi })$	$(0.52e^{i1.44\pi },0.32e^{i0.72\pi })$	$(0.00e^{i0.00\pi },1.00e^{i2.00\pi })$	$(0.79e^{i1.86\pi },0.02e^{i0.01\pi })$	1.30
$y_{4}$	$(0.00e^{i0.00\pi },1.00e^{i2.00\pi })$	$(0.00e^{i0.00\pi },1.00e^{i2.00\pi })$	$(0.69e^{i1.28\pi },0.45e^{i0.86\pi })$	$(0.00e^{i0.00\pi },1.00e^{i2.00\pi })$	$(0.69e^{i1.28\pi },0.45e^{i0.86\pi })$	0.42
$y_{5}$	$(0.00e^{i0.00\pi },1.00e^{i2.00\pi })$	$(0.00e^{i0.00\pi },1.00e^{i2.00\pi })$	$(0.00e^{i0.00\pi },1.00e^{i2.00\pi })$	$(0.81e^{i1.53\pi },0.12e^{i0.34\pi })$	$(0.81e^{i1.53\pi },0.12e^{i0.34\pi })$	0.97

In Table 35, we have chosen $L=3$ for DM and get the 3-CV of CFF6$\hbox {S}_f$S. It can observe from Table 35 that the telecommunication company $y_1= ~ \mathrm{zong}$ has highest output value. So, $y_1$ will be selected by the student.

$\bullet$ **Comparison table of CFF6**$\hbox {S}_f$**S**

Now to apply the third procedure as shown in Algorithm 3, membership and non-membership values of Table 33 are given by Table 36.

**Table 36:** Tabular form of the membership and non-membership values of CFF6$\hbox {S}_f$S $({\mathcal {H}}, {\mathcal {Q}}, 6)$

$({\mathcal {H}}, {\mathcal {Q}}, 6)$	$o_{1}$	$o_{2}$	$o_{3}$	$o_{4}$
$y_{1}$	$(0.95e^{i1.78\pi },0.05e^{i0.24\pi })$	$(0.76e^{i1.69\pi },0.39e^{i0.29\pi })$	$(0.58e^{i1.38\pi },0.23e^{i0.52\pi })$	$(0.91e^{i1.93\pi },0.02e^{i0.05\pi })$
$y_{2}$	$(0.32e^{i0.54\pi },0.89e^{i1.74\pi })$	$(0.24e^{i0.64\pi },0.43e^{i1.22\pi })$	$(0.29e^{i0.54\pi },0.49e^{i0.98\pi })$	$(0.43e^{i0.66\pi },0.85e^{i1.46\pi })$
$y_{3}$	$(0.48e^{i1.06\pi },0.17e^{i0.30\pi })$	$(0.70e^{i1.71\pi },0.32e^{i0.21\pi })$	$(0.52e^{i1.44\pi },0.32e^{i0.72\pi })$	$(0.44e^{i0.44\pi },0.35e^{i1.08\pi })$
$y_{4}$	$(0.21e^{i0.04\pi },0.83e^{i1.94\pi })$	$(0.26e^{i0.56\pi },0.43e^{i1.21\pi })$	$(0.69e^{i1.28\pi },0.45e^{i0.86\pi })$	$(0.19e^{i0.38\pi },0.37e^{i1.06\pi })$
$y_{5}$	$(0.29e^{i0.84\pi },0.57e^{i1.16\pi })$	$(0.34e^{i0.84\pi },0.95e^{i1.71\pi })$	$(0.07e^{i0.24\pi },0.99e^{i1.88\pi })$	$(0.81e^{i1.53\pi },0.12e^{i0.34\pi })$

The comparison table of Table 36 is given by Table 37.

**Table 37:** Comparison table of CFF6$\hbox {S}_f$S $({\mathcal {H}}, {\mathcal {Q}}, 6)$

	$y_{1}$	$y_{2}$	$y_{3}$	$y_{4}$	$y_{5}$
$y_{1}$	4	4	4	4	4
$y_{2}$	0	4	0	2	2
$y_{3}$	0	4	4	4	3
$y_{4}$	0	2	0	4	2
$y_{5}$	0	2	1	2	4

The result will be derived by subtracting the row and column sum of Table 37.

**Table 38:** Ranking Table

	Grade sum $(G_s)$	Row sum $(R_s)$	Column sum $(C_s)$	Final rank $(R_s-C_s)$
$y_{1}$	17	20	4	16
$y_{2}$	6	8	16	$-8$
$y_{3}$	12	15	9	6
$y_{4}$	7	8	16	$-8$
$y_{5}$	7	9	15	$-6$

From Table 38, it is concluded that the highest rank and grade sum is 17 and 16, respectively, which is obtained by $y_1=~ \mathrm{zong}.$ So, sim card $y_1$ is selected by the student.

## Comparison analysis

To certify the rationality of our proposed model, we solve the same example “Selection of the best cellular telecommunication company in Pakistan” using $\hbox {FFY}_w$A (Garg et al. 2020) and $\hbox {FFY}_w$G (Garg et al. 2020) operators:

- **Step 1.** The membership and non-membership terms of amplitude part are the same as given in Table 33, but their grades have neglected and phase terms in all $\hbox {CFFNS}_f$Ns have taken to be zero given by Table 39.
- **Step 2.** Let $\tau _k=(0.2,0.4,0.1,0.3)^t$ is the weight vector on alternatives.
- **Step 3.** The entries of aggregated values $W_i$ of the alternatives by using $\hbox {FFY}_w$A (Garg et al. 2020) operator defined as follows:
  $$\begin{aligned} {\mathcal {W}}_i= \bigg \langle \root 3 \of {\min (1, (\sum \limits _{t=1}^n (\tau _{t}s_{gt}^{3\varsigma }))^{\frac{1}{\varsigma }})}, \root 3 \of {1-\min (1,(\sum \limits _{t=1}^n (\tau _{t}(1-k^{3}_{gt})^{\varsigma }))^{\frac{1}{\varsigma }})}\bigg \rangle \end{aligned}$$
  For $\varsigma =3,$ the values are:
  $$\begin{aligned} {\mathcal {W}}_1= & {} (0.87, 0.29) \\ {\mathcal {W}}_2= & {} (0.38, 0.64) \\ {\mathcal {W}}_3= & {} (0.64, 0.31) \\ {\mathcal {W}}_4= & {} (0.53, 0.51) \\ {\mathcal {W}}_5= & {} (0.71, 0.64) \end{aligned}$$
- **Step 4.** The entries of aggregated values ${\mathcal {W}}_i$ of the alternatives by using $\hbox {FFY}_w$G (Garg et al. 2020) operator defined as follows:
  $$\begin{aligned} {\mathcal {W}}_i= \bigg \langle \root 3 \of {1-\min (1,(\sum \limits _{t=1}^n (\tau _{t}(1-s^{3}_{gt})^{\phi }))^{\frac{1}{\phi }})}, \root 3 \of {\min (1, (\sum \limits _{t=1}^n (\tau _{t}k_{gt}^{3\phi })^{\frac{1}{\phi }}))}\bigg \rangle \end{aligned}$$
  For $\phi =3,$ the values are:
  $$\begin{aligned} {\mathcal {W}}_1= & {} (0.79, 0.35) \\ {\mathcal {W}}_2= & {} (0.33, 0.80) \\ {\mathcal {W}}_3= & {} (0.57, 0.33) \\ {\mathcal {W}}_4= & {} (0.33, 0.69) \\ {\mathcal {W}}_5= & {} (0.50, 0.89) \end{aligned}$$
- **Step 5.** The score of each executed value from $\hbox {FFY}_w$A and $\hbox {FFY}_w$G operators are assembled in Table 40.

**Table 39:** Fermatean fuzzy decision matrix

${Y}/{\mathcal {O}}$	$o_{1}$	$o_{2}$	$o_{3}$	$o_{4}$
$y_{1}$	(0.95, 0.05)	(0.76, 0.39)	(0.58, 0.23)	(0.91, 0.02)
$y_{2}$	(0.32, 0.89)	(0.24, 0.43)	(0.29, 0.49)	(0.43, 0.85)
$y_{3}$	(0.48, 0.17)	(0.70, 0.32)	(0.52, 0.32)	(0.44, 0.35)
$y_{4}$	(0.21, 0.83)	(0.26, 0.43)	(0.69, 0.45)	(0.19, 0.37)
$y_{5}$	(0.29, 0.57)	(0.34, 0.95)	(0.07, 0.99)	(0.81, 0.12)

**Table 40:** Score values

Methods	$S({\mathcal {W}}_1)$	$S({\mathcal {W}}_2)$	$S({\mathcal {W}}_3)$	$S({\mathcal {W}}_4)$	$S({\mathcal {W}}_5)$	Best alternative
$\hbox {FFY}_w$A operator	0.63	$-0.21$	0.23	0.02	0.10	$y_1$
$\hbox {FFY}_w$G operator	0.45	$-0.48$	0.15	$-0.29$	$-0.58$	$y_1$

### Discussion

**Fig. 1:**
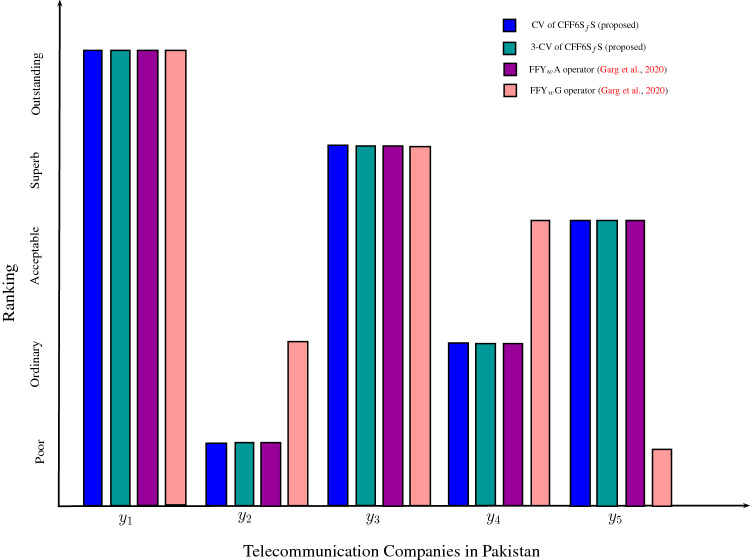
Comparison chart

1. We present a comparative study with existing MADM techniques, namely, $\hbox {FFY}_w$A and $\hbox {FFY}_w$G operators which manifest the proficiency and adeptness of proposed methods. The ranking of alternatives by applying the proposed and compared techniques are given in Table 41.
2. According to Table 41, Zong $(y_1)$ is the best alternative obtained from the extant and proposed model which shows the validity and authenticity of proposed MADM methods.
3. Figure 1 skillfully depicts the comparison between the outcomes of proposed and existing decision-making methodologies by displaying an illustrated bar chart among network companies and their order of ranking, demonstrating the consistency and competency of the presented MADM techniques.
4. Our proposed approaches provide the most comprehensive and adaptable methodologies because they effectively incorporate the existing proficient MADM methods, namely, $\hbox {FFY}_w$A operators and $\hbox {FFY}_w$G operators, by taking phase term equal to zero and neglecting the parameterized grading of alternatives. On the contrary, existing techniques cannot handle the two-dimensional parameterized fuzzy information. They are designed to deal with one-dimensional information only.

**Table 41:** Comparison analysis with existing methods

Methods	$S({\mathcal {W}}_1)$	$S({\mathcal {W}}_2)$	$S({\mathcal {W}}_3)$	$S({\mathcal {W}}_4)$	$S({\mathcal {W}}_5)$	Ranking order	Best alternative
CV of CFF6$\hbox {S}_f$S (proposed)	2.96	0.30	1.87	0.84	1.59	$y_1>y_3>y_5>y_4>y_2$	$y_1$
3-CV of CFF6$\hbox {S}_f$S (proposed)	1.96	$-2.00$	1.30	0.42	0.97	$y_1>y_3>y_5>y_4>y_2$	$y_1$
$\hbox {FFY}_w$A operator (Garg et al. 2020)	0.63	$-0.21$	0.23	0.02	0.10	$y_1>y_3>y_5>y_4>y_2$	$y_1$
$\hbox {FFY}_w$G operator (Garg et al. 2020)	0.45	$-0.48$	0.15	$-0.29$	$-0.58$	$y_1>y_3>y_4>y_2>y_5$	$y_1$

## Development of $\hbox {CFFNS}_f$-TOPSIS method for MAGDM problems

In this section, we aim to renovate the TOPSIS approach for the environment of $\hbox {CFFNS}_f$ to account for MAGDM problems. The chief idea of the proposed $\hbox {CFFNS}_f$-TOPSIS technique is to find the most appropriate alternative having maximum distance from negative ideal solution (NIS) and shortest distance from positive ideal solution (PIS). Mathematical steps of MAGDM are as follows:

Let ${\mathfrak {D}}=\{{\mathfrak {D}}_1, {\mathfrak {D}}_2, \ldots , {\mathfrak {D}}_l\}$ be the set of *l* distinct experts which are appointed for the selection of best alternative from ${\mathfrak {I}}=\{{\mathfrak {I}}_1,{\mathfrak {I}}_2,{\mathfrak {I}}_3,\ldots , {\mathfrak {I}}_n\}$ relating to some specific attributes. ${\mathfrak {B}}=\{{\mathfrak {B}}_1,{\mathfrak {B}}_2,{\mathfrak {B}}_3,\ldots ,{\mathfrak {B}}_m\}$ represents the collection of attributes which is selected by the experts according to the necessities of decision-making problem and $\zeta =(\zeta _1, \zeta _2, \ldots , \zeta _l)^T$ be the weight vector, represents the weightage of experts such that $\sum \limits _{\mathfrak {{\mathfrak {c}}}=1}^l \zeta _{\mathfrak {c}} =1.$

The step-by-step procedure of $\hbox {CFFNS}_f$-TOPSIS method is as follows:

- **Step 1.** Firstly, decision-makers will give grades corresponding to the linguistic terms according to the importance of alternatives on the basis of attributes. Then each expert ${\mathfrak {D}}_{\mathfrak {c}}$ will assign $\hbox {CFFNS}_f$N, corresponding to each grade in $\hbox {NS}_f$S $({\mathcal {H}}^{({\mathfrak {c}})}, {\mathcal {Q}}, N)$, according to the defined criteria for ranking. The $\hbox {CFFNS}_f$Ns allocated by the decision-maker ${\mathfrak {D}}_{\mathfrak {c}}$ are adapted in complex Fermatean fuzzy $\hbox {NS}_f$ decision matrix ($\hbox {CFFNS}_f$DM) ${\mathfrak {G}}^{({\mathfrak {c}})}=({\mathfrak {G}}_{gt}^{({\mathfrak {c}})})_{n \times m}.$ Hence, $\hbox {CFFNS}_f$DMs ${\mathfrak {G}}^{(1)}, {\mathfrak {G}}^{(2)}, \ldots , {\mathfrak {G}}^{(l)}$ are arranged by *l* decision-experts as follows:
  1 $$\begin{aligned} {\mathfrak {G}}^{({\mathfrak {c}})} \nonumber =\left( \begin{array}{cccc} \langle r_{a_{11}}^{({\mathfrak {c}})}, (\mu _{a_{11}}^{({\mathfrak {c}})}, \nu _{a_{11}}^{({\mathfrak {c}})}) \rangle &{} \langle r_{a_{12}}^{({\mathfrak {c}})}, (\mu _{a_{12}}^{({\mathfrak {c}})}, \nu _{a_{12}}^{({\mathfrak {c}})}) \rangle &{} \cdots &{} \langle r_{a_{1m}}^{({\mathfrak {c}})}, (\mu _{a_{1m}}^{({\mathfrak {c}})}, \nu _{a_{1m}}^{({\mathfrak {c}})}) \rangle \\ \langle r_{a_{21}}^{({\mathfrak {c}})}, (\mu _{a_{21}}^{({\mathfrak {c}})}, \nu _{a_{21}}^{({\mathfrak {c}})}) \rangle &{} \langle r_{a_{22}}^{({\mathfrak {c}})}, (\mu _{a_{22}}^{({\mathfrak {c}})}, \nu _{a_{22}}^{({\mathfrak {c}})}) \rangle &{} \cdots &{} \langle r_{a_{2m}}^{({\mathfrak {c}})}, (\mu _{a_{2m}}^{({\mathfrak {c}})}, \nu _{a_{2m}}^{({\mathfrak {c}})}) \rangle \\ \vdots &{} \vdots &{} \ddots &{} \vdots \\ \langle r_{a_{n1}}^{({\mathfrak {c}})}, (\mu _{a_{n1}}^{({\mathfrak {c}})}, \nu _{a_{n1}}^{({\mathfrak {c}})}) \rangle &{} \langle r_{a_{n2}}^{({\mathfrak {c}})}, (\mu _{a_{n2}}^{({\mathfrak {c}})}, \nu _{a_{n2}}^{({\mathfrak {c}})}) \rangle &{} \cdots &{} \langle r_{a_{nm}}^{({\mathfrak {c}})}, (\mu _{a_{nm}}^{({\mathfrak {c}})}, \nu _{a_{nm}}^{({\mathfrak {c}})}) \rangle \\ \end{array} \right) \\ \end{aligned}$$
  where $${\mathfrak {c}}=\{1,2,\ldots ,l\}.$$
  Each entry of the $\hbox {CFFNS}_f$DM has the form ${\mathfrak {G}}_{gt}^{({\mathfrak {c}})}=\langle r_{a_{gt}}^{({\mathfrak {c}})}, (\mu _{a_{gt}}^{({\mathfrak {c}})}, \nu _{a_{gt}}^{({\mathfrak {c}})}) \rangle =\langle r_{a_{gt}}^{({\mathfrak {c}})}, (s_{a_{gt}}^{({\mathfrak {c}})}e^{i\omega _{a_{gt}}^{({\mathfrak {c}})}}, k_{a_{gt}}^{({\mathfrak {c}})}e^{i\psi _{a_{gt}}^{({\mathfrak {c}})}}) \rangle .$
- **Step 2.** For MAGDM, the individual opinions of the decision-makers are organized to have a generic opinion of all experts about an alternative related to the attributes. This directs to the formation of aggregated $\hbox {CFFNS}_f$DM ($\hbox {ACFFNS}_f$DM) ${\mathfrak {G}}=({\mathfrak {G}})_{n \times m}$. The $\hbox {CFFNS}_f$DM of all experts are assembled with the help of $\hbox {CFFNS}_f$ weighted average ($\hbox {CFFNS}_f$WA) operator as follows:
  2 $$\begin{aligned} {\mathfrak {G}}_{gt}= & {} CFFNS_fWA_\zeta ({\mathfrak {G}}_{gt}^{(1)},{\mathfrak {G}}_{gt}^{(2)}, \ldots , {\mathfrak {G}}_{gt}^{(l)}) \nonumber \\= & {} \zeta _1{\mathfrak {G}}_{gt}^{(1)}\oplus \zeta _2 {\mathfrak {G}}_{gt}^{(2)} \oplus \ldots \oplus \zeta _l{\mathfrak {G}}_{gt}^{(l)}\nonumber \\= & {} \bigg \langle \max \limits _{{\mathfrak {c}}=1}^l r_{a_{gt}}^{({\mathfrak {c}})}, \bigg (\root 3 \of {1-\prod \limits _{{\mathfrak {c}}=1}^l(1 -(s_{a_{gt}}^{({\mathfrak {c}})})^3)^{\zeta _{\mathfrak {c}}}}e^{i2\pi \root 3 \of {1-\prod \limits _{{\mathfrak {c}}=1}^l(1 -(\frac{\omega _{a_{gt}}^{({\mathfrak {c}})}}{2\pi })^3)^{\zeta _{\mathfrak {c}}}}}, \prod \limits _{{\mathfrak {c}}=1}^l (k_{a_{gt}}^{({\mathfrak {c}})})^{\zeta _{\mathfrak {c}}}e^{i2\pi \prod \limits _{{\mathfrak {c}}=1}^l(\frac{\psi _{a_{gt}}^{({\mathfrak {c}})}}{2\pi })^{\zeta _{\mathfrak {c}}}}\bigg )\bigg \rangle , \end{aligned}$$
  where ${\mathfrak {G}}_{gt}=\langle r_{a_{gt}}, (\mu _{a_{gt}}, \nu _{a_{gt}}) \rangle =\langle r_{a_{gt}}, (s_{a_{gt}}e^{i\omega _{a_{gt}}},k_{a_{gt}}e^{i\psi _{a_{gt}}}) \rangle , g=1,2,3, \dots , n$, and $t=1,2,3, \dots , m.$ The $\hbox {ACFFNS}_f$DM can be form as follows:
  $$\begin{aligned} {\mathfrak {G}}=\left( \begin{array}{cccc} \langle r_{a_{11}}, (\mu _{a_{11}}, \nu _{a_{11}}) \rangle &{} \langle r_{a_{12}}, (\mu _{a_{12}}, \nu _{a_{12}}) \rangle &{} \cdots &{} \langle r_{a_{1m}}, (\mu _{a_{1m}}, \nu _{a_{1m}}) \rangle \\ \langle r_{a_{21}}, (\mu _{a_{21}}, \nu _{a_{21}}) \rangle &{} \langle r_{a_{22}}, (\mu _{a_{22}}, \nu _{a_{22}}) \rangle &{} \cdots &{} \langle r_{a_{2m}}, (\mu _{a_{2m}}, \nu _{a_{2m}}) \rangle \\ \vdots &{} \vdots &{} \ddots &{} \vdots \\ \langle r_{a_{n1}}, (\mu _{a_{n1}}, \nu _{a_{n1}}) \rangle &{} \langle r_{a_{n2}}, (\mu _{a_{n2}}, \nu _{a_{n2}}) \rangle &{} \cdots &{} \langle r_{a_{nm}}, (\mu _{a_{nm}}, \nu _{a_{nm}}) \rangle \\ \end{array} \right) \end{aligned}$$
- **Step 3.** The attributes nominated by experts may not be equally important and valuable in a MAGDM problem. Therefore, each decision-maker ranks these attributes and assigns a $\hbox {CFFNS}_f$ weightage according to the grading criteria defined by the experts. Let $\kappa _t^{({\mathfrak {c}})}= \langle r_{a_t}^{({\mathfrak {c}})}, (\mu _{a_t}^{({\mathfrak {c}})}, \nu _{a_t}^{({\mathfrak {c}})}) \rangle = \langle r_{a_t}^{({\mathfrak {c}})}, (s_{a_t}^{({\mathfrak {c}})}e^{i\omega _{a_t}^{({\mathfrak {c}})}}, k_{a_t}^{({\mathfrak {c}})}e^{i\psi _{a_t}^{({\mathfrak {c}})}} \rangle$ be the $\hbox {CFFNS}_f$ weight assigned by decision-maker ${\mathfrak {D}}_{\mathfrak {c}}$ to the attribute ${\mathfrak {B}}_t.$ To compute the $\hbox {CFFNS}_f$ weight vector $\kappa =(\kappa _1, \kappa _2, \kappa _3, \ldots , \kappa _m)^T$ of attributes, the $\hbox {CFFNS}_f$Ns corresponding to the grade values, assigned by decision-makers are aggregated as follows:
  3 $$\begin{aligned} \kappa _t= & {} CFFNS_fWA_{\zeta }(\kappa ^{(1)}_t,\kappa ^{(2)}_t, \ldots , \kappa ^{(l)}_t) \nonumber \\&= {} \zeta _1\kappa ^{(1)}_t \oplus \zeta _2\kappa ^{(2)}_t \oplus \ldots \oplus \zeta _l\kappa ^{(l)}_t\nonumber \\&= {} \bigg \langle \max \limits _{{\mathfrak {c}}=1}^l r_{a_{t}}^{({\mathfrak {c}})}, \bigg (\root 3 \of {1-\prod \limits _{{\mathfrak {c}}=1}^l(1 -(s_{a_{t}}^{({\mathfrak {c}})})^3)^{\zeta _{\mathfrak {c}}}}e^{i2\pi \root 3 \of {1-\prod \limits _{{\mathfrak {c}}=1}^l(1 -(\frac{\omega _{a_{t}}^{({\mathfrak {c}})}}{2 \pi })^3)^{\zeta _{\mathfrak {c}}}}}, \prod \limits _{{\mathfrak {c}}=1}^l (k_{a_{t}}^{({\mathfrak {c}})})^{\zeta _{\mathfrak {c}}}e^{i2\pi \prod \limits _{{\mathfrak {c}}=1}^l(\frac{\psi _{a_{t}}^{({\mathfrak {c}})}}{2\pi })^{\zeta _{\mathfrak {c}}}}\bigg )\bigg \rangle ,\nonumber \\&= {} \langle r_{a_{t}}, (\mu _{a_{t}}, \nu _{a_{t}}) \rangle , \nonumber \\&= {} \langle r_{a_{t}},(s_{a_{t}}e^{i\omega _{a_{t}}},k_{a_{t}}e^{i\psi _{a_{t}}}) \rangle , \end{aligned}$$
  where $$t=1,2,3,\dots ,m.$$
- **Step 4.** Construct the aggregated weighted $\hbox {CFFNS}_f$DM ($\hbox {AWCFFNS}_f$DM) $\widehat{{\mathfrak {G}}}=(\widehat{{\mathfrak {G}}}_{gt})_{n \times m}$ by using $\hbox {CFFNS}_f$DM ${\mathfrak {G}}$ and the weight vector $\kappa _t$ of attributes, as follows:
  4 $$\begin{aligned} \widehat{{\mathfrak {G}}}_{gt}= & {} {\mathfrak {G}}_{gt} \otimes \kappa _t \nonumber \\= & {} \bigg \langle \min (r_{a_{gt}},r_{a_{t}}), \bigg (s_{a_{gt}}s_{a_{t}}e^{i2\pi (\frac{\omega _{a_{gt}}}{2\pi }) (\frac{\omega _{a_{t}}}{2\pi })},\root 3 \of {(k_{a_{gt}})^3+(k_{a_{t}})^3 -(k_{a_{gt}})^3(k_{a_{t}})^3}\nonumber \\&e^{i2\pi \root 3 \of {(\frac{\psi _{a_{gt}}}{2\pi })^3 +(\frac{\psi _{a_{t}}}{2\pi })^3-(\frac{\psi _{a_{gt}}}{2\pi })^3 (\frac{\psi _{a_{t}}}{2\pi })^3}}\bigg )\bigg \rangle ,\nonumber \\= & {} \langle {\hat{r}}_{a_{gt}}, ({\hat{\mu }}_{a_{gt}}, {\hat{\nu }}_{a_{gt}}) \rangle , \nonumber \\= & {} \langle {\hat{r}}_{a_{gt}}, ({\hat{s}}_{a_{gt}}e^{i{\hat{\omega }}_{a_{gt}}}, {\hat{k}}_{a_{gt}}e^{i{\hat{\psi }}_{a_{gt}}}) \rangle . \end{aligned}$$
  The $\hbox {AWCFFNS}_f$DM is constructed as follows:
  $$\begin{aligned} \widehat{{\mathfrak {G}}} =\left( \begin{array}{cccc} \langle {\hat{r}}_{a_{11}}, ({\hat{\mu }}_{a_{11}}, {\hat{\nu }}_{a_{11}}) \rangle &{} \langle {\hat{r}}_{a_{12}}, ({{\hat{\mu }}}_{a_{12}}, {{\hat{\nu }}}_{a_{12}}) \rangle &{} \cdots &{} \langle {\hat{r}}_{a_{1m}}, ({\hat{\mu }}_{a_{1m}}, {\hat{\nu }}_{a_{1m}}) \rangle \\ \langle {\hat{r}}_{a_{21}}, ({\hat{\mu }}_{a_{21}}, {\hat{\nu }}_{a_{21}}) \rangle &{} \langle {\hat{r}}_{a_{22}}, ({\hat{\mu }}_{a_{22}}, {\hat{\nu }}_{a_{22}}) \rangle &{} \cdots &{} \langle {\hat{r}}_{a_{2m}}, ({\hat{\mu }}_{a_{2m}}, {\hat{\nu }}_{a_{2m}}) \rangle \\ \vdots &{} \vdots &{} \ddots &{} \vdots \\ \langle {\hat{r}}_{a_{n1}}, ({\hat{\mu }}_{a_{n1}}, {\hat{\nu }}_{a_{n1}}) \rangle &{} \langle {\hat{r}}_{a_{n2}}, ({\hat{\mu }}_{a_{n2}}, {\hat{\nu }}_{a_{n2}}) \rangle &{} \cdots &{} \langle {\hat{r}}_{a_{nm}}, ({\hat{\mu }}_{a_{nm}}, {\hat{\nu }}_{a_{nm}}) \rangle \\ \end{array} \right) \\ \end{aligned}$$
- **Step 5.** Let ${\mathfrak {B}}^-$ and ${\mathfrak {B}}^+$ represent the collection of cost-type and benefit-type attributes, respectively. Then $\hbox {CFFNS}_f$ positive ideal solution ($\hbox {CFFNS}_f$-PIS) $\widetilde{{\mathfrak {G}}}_t=\langle {\tilde{r}}_{a_t},({\tilde{\mu }}_{a_t}, {\tilde{\nu }}_{a_t})\rangle$ related to attribute ${\mathfrak {B}}_t$ can be chosen as follows:
  5 $$\begin{aligned} \widetilde{{\mathfrak {G}}}_t=\left\{ \begin{array}{ll} \max \limits _{1\le g \le n} \widehat{{\mathfrak {G}}}_{gt}, &{} \hbox {if }{\mathfrak {B}}_t \in {\mathfrak {B}}^+, \\ \min \limits _{1\le g \le n} \widehat{{\mathfrak {G}}}_{gt}, &{} \hbox {if }{\mathfrak {B}}_t \in {\mathfrak {B}}^-. \end{array} \right. \end{aligned}$$
  The $\hbox {CFFNS}_f$ negative ideal solution ($\hbox {CFFNS}_f$-NIS) $\breve{{\mathfrak {G}}}_t=\langle \breve{r}_{a_t},(\breve{\mu }_{a_t}, \breve{\nu }_{a_t})\rangle$ with respect to the attribute ${\mathfrak {B}}_t$ can be determined as follows:
  6 $$\begin{aligned} \breve{{\mathfrak {G}}}_t=\left\{ \begin{array}{ll} \min \limits _{1\le g \le n} \widehat{{\mathfrak {G}}}_{gt}, &{} \hbox {if }{\mathfrak {B}}_t \in {\mathfrak {B}}^+, \\ \max \limits _{1\le g \le n} \widehat{{\mathfrak {G}}}_{gt}, &{} \hbox {if }{\mathfrak {B}}_t \in {\mathfrak {B}}^-. \end{array} \right. \end{aligned}$$
  The $\hbox {CFFNS}_f$Ns are compared on the basis of accuracy function and score function to obtain $\hbox {CFFNS}_f$-PIS and $\hbox {CFFNS}_f$-NIS.
- **Step 6.** Now, to find the optimal alternative which is away from $\hbox {CFFNS}_f$-NIS and closest to $\hbox {CFFNS}_f$-PIS, we evaluate the distance of each alternative ${\mathfrak {I}}_g$ from $\hbox {CFFNS}_f$-PIS and $\hbox {CFFNS}_f$-NIS. The distance between any of the alternative and $\hbox {CFFNS}_f$-PIS can be calculated as follows:
  7 $$\begin{aligned} d({\mathfrak {I}}_g, \widetilde{{\mathfrak {G}}}_t) = \sum \limits _{t=1}^m&\sqrt{\frac{1}{3} \bigg \{\big (\frac{{\hat{r}}_{a_{gt}}}{N-1} -\frac{{\tilde{r}}_{a_t}}{N-1}\big )^2+({\hat{s}}_{a_{gt}}^3 -{\tilde{s}}_{a_t}^3)^2+ ({\hat{k}}_{a_{gt}}^3-{\tilde{k}}_{a_{t}}^3)^2 +\frac{1}{64\pi ^6}}\nonumber \\&\sqrt{\bigg (({{\hat{\omega }}_{a_{gt}}}^3-{{\tilde{\omega }}_{a_t}}^3)^2+ ({{\hat{\psi }}_{a_{gt}}}^3-{{\tilde{\psi }}_{a_{t}}}^3)^2\bigg )\bigg \}} \end{aligned}$$
  Similarly, the distance between any of the alternative and $\hbox {CFFNS}_f$-NIS can be calculated as follows:
  8 $$\begin{aligned} d({\mathfrak {I}}_g, \breve{{\mathfrak {G}}}_t)= \sum \limits _{t=1}^m&\sqrt{\frac{1}{3} \bigg \{\big (\frac{{\hat{r}}_{a_{gt}}}{N-1} -\frac{\breve{r}_{a_t}}{N-1}\big )^2+({\hat{s}}_{a_{gt}}^3 -\breve{s}_{a_t}^3)^2+ ({\hat{k}}_{a_{gt}}^3-\breve{k}_{a_{t}}^3)^2 +\frac{1}{64\pi ^6}}\nonumber \\&\sqrt{\bigg (({\hat{\omega }}_{a_{gt}}^3-\breve{\omega }_{a_t}^3)^2+ ({\hat{\psi }}_{a_{gt}}^3-\breve{\psi }_{a_{t}}^3)^2\bigg )\bigg \}} \end{aligned}$$
- **Step 7.** To find the most suitable alternative, we compare the alternative by some ranking index. The revised closeness index (Vencheh and Mirjaberi 2014) corresponding to the alternative ${\mathfrak {I}}_g$ can be evaluated by utilizing the formula:
  9 $$\begin{aligned} \varPsi ({\mathfrak {I}}_g)= \frac{d({\mathfrak {I}}_g, \breve{{\mathfrak {G}}}_t)}{d_{\max }({\mathfrak {I}}_g, \breve{{\mathfrak {G}}}_t)}-\frac{d({\mathfrak {I}}_g, \widetilde{{\mathfrak {G}}}_t)}{d_{\min }({\mathfrak {I}}_g, \widetilde{{\mathfrak {G}}}_t)}, \end{aligned}$$
  where $$g=1,2,3,\ldots ,n$$, and
  $$\begin{aligned} d_{\max }({\mathfrak {I}}_g, \breve{{\mathfrak {G}}}_t)= & {} \max \limits _{1\le g\le n}({\mathfrak {I}}_g, \breve{{\mathfrak {G}}}_t), \\ d_{\min }({\mathfrak {I}}_g, \breve{{\mathfrak {G}}}_t)= & {} \min \limits _{1\le g\le n}({\mathfrak {I}}_g, \widetilde{{\mathfrak {G}}}_t). \end{aligned}$$
- **Step 8.** After the evaluated results of closeness index, the alternatives are arranged in an ascending order with respect to revised closeness index. The alternative having maximum value of closeness index will be the optimal solution of MAGDM problem.

**Fig. 2:**
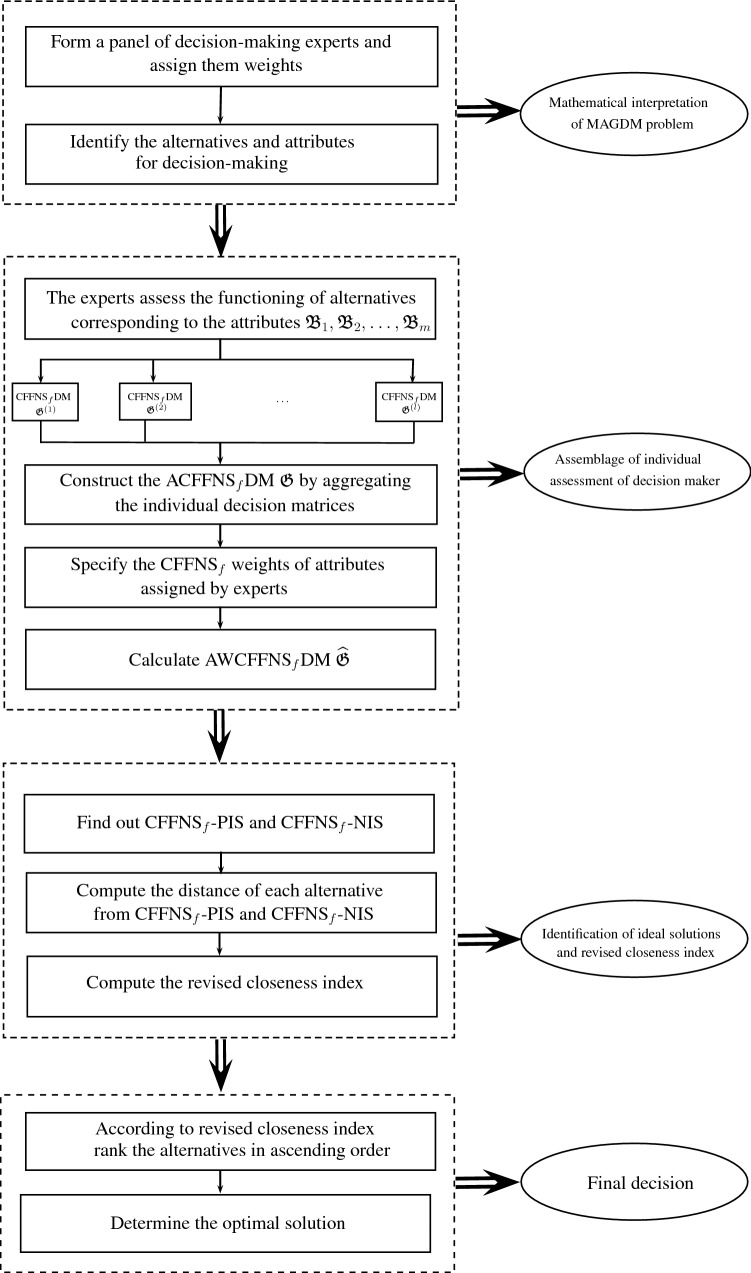
Flow chart of $\hbox {CFFNS}_f$-TOPSIS method

The general steps of $\hbox {CFFNS}_f$-TOPSIS method are summarized in Figure 2.

## Selection of the most suitable city in the USA for farming

Suppose that an investor *X* wants to purchase land for agriculture farming in a favorable city of the USA. For this purpose, he arranged a panel of four decision-makers ${\mathfrak {D}}_1, {\mathfrak {D}}_2, {\mathfrak {D}}_3$ and ${\mathfrak {D}}_4$ to thoroughly judge the essential needs of the best land for farming. Since, each decision-maker has his own importance and opinions, so, $\zeta =(0.24~~0.35~~0.23~~0.18)^T$ represents the weightage of experts in the decision-making panel. Clearly, $\sum \limits _{{\mathfrak {c}}=1}^4 \zeta _{{\mathfrak {c}}}=1.$ The following cities are under consideration as alternatives for this problem:

- $${\mathfrak {I}}_1:$$ Boston, Massachusetts
- $${\mathfrak {I}}_2:$$ Portland, Oregon
- ${\mathfrak {I}}_3:$ New York city, New York
- $${\mathfrak {I}}_4:$$ Minneapolis, Minnesota

After discussion, all decision-makers identify the following factors as the attributes for this MAGDM problem:

- $${\mathfrak {B}}_1:$$ Initial cost
- $${\mathfrak {B}}_2:$$ Environmental destruction
- $${\mathfrak {B}}_3:$$ Topography
- ${\mathfrak {B}}_4:$ Climate of the Area
- $${\mathfrak {B}}_5:$$ Maintenance cost
- $${\mathfrak {B}}_6:$$ Soil
- ${\mathfrak {B}}_7:$ Water quality & availability

The stepwise solution of this MAGDM problem by following $\hbox {CFFNS}_f$-TOPSIS method is given as follows:

- **Step 1.** According to the above-mentioned attributes, each expert assesses the alternatives regarding all attributes using 5-soft, given in Table 42, where:

- $$\heartsuit \heartsuit \heartsuit \heartsuit$$ represent ‘Outstanding’,
- $$\heartsuit \heartsuit \heartsuit$$ represent ‘Good’,
- $$\heartsuit \heartsuit$$ represent ‘Average’,
- $$\heartsuit$$ represents ‘Satisfactory’,
- $\diamond$ represents ‘Below average.

The experts ${\mathfrak {D}}_1, {\mathfrak {D}}_2, {\mathfrak {D}}_3$ and ${\mathfrak {D}}_4$ will use the grading criteria given by Table 5, to assign the $\hbox {CFFNS}_f$N corresponding to each rank. The individual $\hbox {CFFNS}_f$DMs of the experts are arranged by Tables 43–46.

**Table 42:** Expert’s assessment of alternatives corresponding to each attribute

Attributes	Alternatives	${\mathfrak {D}}_1$	${\mathfrak {D}}_2$	${\mathfrak {D}}_3$	${\mathfrak {D}}_4$
${\mathfrak {B}}_1$	${\mathfrak {I}}_1$	$\heartsuit =1$	$\heartsuit =1$	$\heartsuit \heartsuit =2$	$\heartsuit \heartsuit =2$
	${\mathfrak {I}}_2$	$\heartsuit \heartsuit =2$	$\heartsuit \heartsuit \heartsuit =3$	$\heartsuit \heartsuit \heartsuit =3$	$\heartsuit \heartsuit \heartsuit =3$
	${\mathfrak {I}}_3$	$\heartsuit \heartsuit \heartsuit =3$	$\heartsuit \heartsuit \heartsuit \heartsuit =4$	$\heartsuit \heartsuit \heartsuit \heartsuit =4$	$\heartsuit \heartsuit \heartsuit \heartsuit =4$
	${\mathfrak {I}}_4$	$\heartsuit \heartsuit =2$	$\heartsuit \heartsuit =2$	$\heartsuit \heartsuit =2$	$\heartsuit \heartsuit =2$
${\mathfrak {B}}_2$	${\mathfrak {I}}_1$	$\heartsuit \heartsuit =2$	$\heartsuit \heartsuit =2$	$\heartsuit \heartsuit =2$	$\heartsuit \heartsuit =2$
	${\mathfrak {I}}_2$	$\heartsuit \heartsuit =2$	$\heartsuit =1$	$\heartsuit \heartsuit =2$	$\heartsuit \heartsuit =2$
	${\mathfrak {I}}_3$	$\heartsuit \heartsuit =2$	$\heartsuit \heartsuit =2$	$\heartsuit \heartsuit =2$	$\heartsuit \heartsuit \heartsuit =3$
	${\mathfrak {I}}_4$	$\heartsuit \heartsuit =2$	$\heartsuit \heartsuit =2$	$\heartsuit \heartsuit =2$	$\heartsuit \heartsuit \heartsuit =3$
${\mathfrak {B}}_3$	${\mathfrak {I}}_1$	$\heartsuit \heartsuit \heartsuit =3$	$\heartsuit \heartsuit \heartsuit =3$	$\heartsuit \heartsuit \heartsuit =3$	$\heartsuit \heartsuit \heartsuit \heartsuit =4$
	${\mathfrak {I}}_2$	$\heartsuit \heartsuit =2$	$\heartsuit \heartsuit =2$	$\heartsuit \heartsuit =2$	$\heartsuit \heartsuit =2$
	${\mathfrak {I}}_3$	$\heartsuit =1$	$\heartsuit =1$	$\diamond =0$	$\diamond =0$
	${\mathfrak {I}}_4$	$\heartsuit \heartsuit =2$	$\heartsuit \heartsuit =2$	$\heartsuit \heartsuit =2$	$\heartsuit \heartsuit =2$
${\mathfrak {B}}_4$	${\mathfrak {I}}_1$	$\heartsuit \heartsuit =2$	$\heartsuit \heartsuit =2$	$\heartsuit =1$	$\heartsuit =1$
	${\mathfrak {I}}_2$	$\heartsuit \heartsuit \heartsuit =3$	$\heartsuit \heartsuit \heartsuit =3$	$\heartsuit \heartsuit \heartsuit =3$	$\heartsuit \heartsuit \heartsuit \heartsuit =4$
	${\mathfrak {I}}_3$	$\heartsuit \heartsuit \heartsuit =3$	$\heartsuit \heartsuit \heartsuit \heartsuit =4$	$\heartsuit \heartsuit \heartsuit \heartsuit =4$	$\heartsuit \heartsuit \heartsuit =3$
	${\mathfrak {I}}_4$	$\heartsuit \heartsuit \heartsuit =3$	$\heartsuit \heartsuit \heartsuit =3$	$\heartsuit \heartsuit \heartsuit =3$	$\heartsuit \heartsuit \heartsuit \heartsuit =4$
${\mathfrak {B}}_5$	${\mathfrak {I}}_1$	$\heartsuit \heartsuit \heartsuit =3$	$\heartsuit \heartsuit \heartsuit \heartsuit =4$	$\heartsuit \heartsuit \heartsuit \heartsuit =4$	$\heartsuit \heartsuit \heartsuit \heartsuit =4$
	${\mathfrak {I}}_2$	$\heartsuit \heartsuit =2$	$\heartsuit \heartsuit \heartsuit =3$	$\heartsuit \heartsuit =2$	$\heartsuit \heartsuit \heartsuit =3$
	${\mathfrak {I}}_3$	$\diamond =0$	$\diamond =0$	$\diamond =0$	$\diamond =0$
	${\mathfrak {I}}_4$	$\heartsuit \heartsuit \heartsuit =3$	$\heartsuit \heartsuit \heartsuit =3$	$\heartsuit \heartsuit \heartsuit =3$	$\heartsuit \heartsuit =2$
${\mathfrak {B}}_6$	${\mathfrak {I}}_1$	$\heartsuit \heartsuit \heartsuit =3$	$\heartsuit \heartsuit \heartsuit =3$	$\heartsuit \heartsuit \heartsuit \heartsuit =4$	$\heartsuit \heartsuit \heartsuit =3$
	${\mathfrak {I}}_2$	$\heartsuit \heartsuit =2$	$\heartsuit =1$	$\heartsuit \heartsuit =2$	$\heartsuit =1$
	${\mathfrak {I}}_3$	$\heartsuit \heartsuit =2$	$\heartsuit =1$	$\heartsuit =1$	$\heartsuit \heartsuit =2$
	${\mathfrak {I}}_4$	$\heartsuit \heartsuit \heartsuit =3$	$\heartsuit \heartsuit =2$	$\heartsuit \heartsuit =2$	$\heartsuit \heartsuit =2$
${\mathfrak {B}}_7$	${\mathfrak {I}}_1$	$\heartsuit \heartsuit \heartsuit \heartsuit =4$	$\heartsuit \heartsuit \heartsuit \heartsuit =4$	$\heartsuit \heartsuit \heartsuit \heartsuit =4$	$\heartsuit \heartsuit \heartsuit \heartsuit =4$
	${\mathfrak {I}}_2$	$\heartsuit \heartsuit =2$	$\heartsuit \heartsuit =2$	$\heartsuit \heartsuit \heartsuit =3$	$\heartsuit \heartsuit =2$
	${\mathfrak {I}}_3$	$\diamond =0$	$\diamond =0$	$\heartsuit =1$	$\heartsuit =1$
	${\mathfrak {I}}_4$	$\heartsuit \heartsuit \heartsuit =3$	$\heartsuit \heartsuit \heartsuit =3$	$\heartsuit \heartsuit \heartsuit \heartsuit =4$	$\heartsuit \heartsuit \heartsuit \heartsuit =4$

**Table 43:**
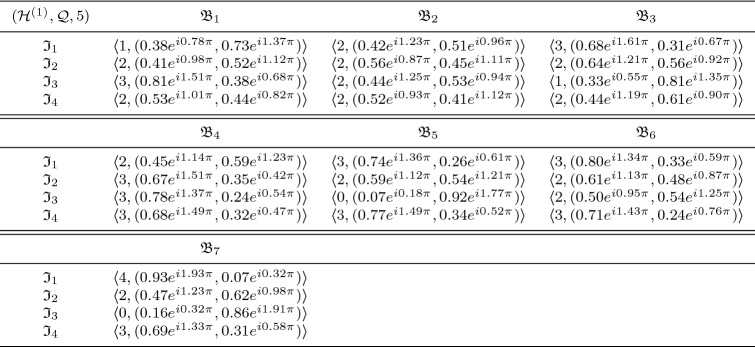
Tabulated form of $\hbox {CFFNS}_f$DM ${\mathfrak {G}}^{(1)}$ of expert ${\mathfrak {D}}_1$

**Table 44:**
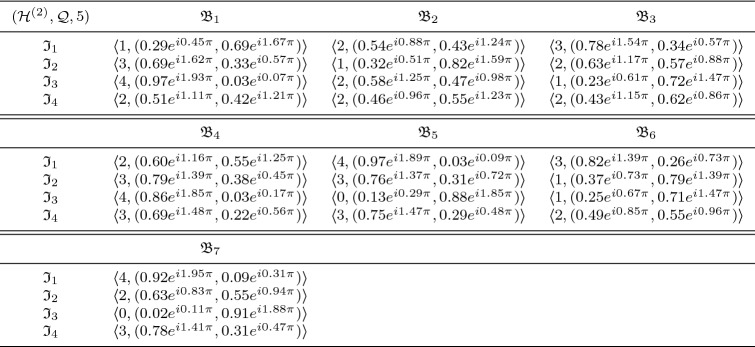
Tabulated form of $\hbox {CFFNS}_f$DM ${\mathfrak {G}}^{(2)}$ of expert ${\mathfrak {D}}_2$

**Table 45:**
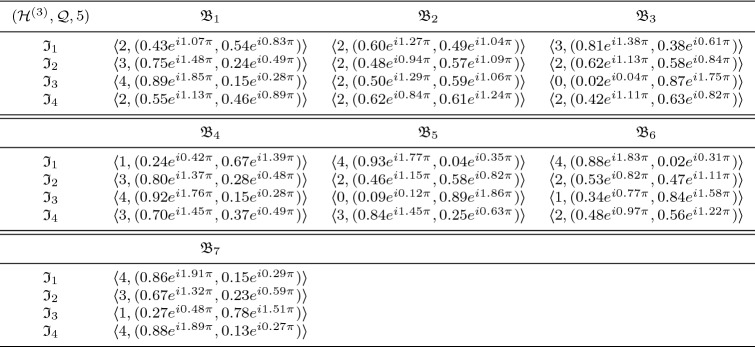
Tabulated form of $\hbox {CFFNS}_f$DM ${\mathfrak {G}}^{(3)}$ of expert ${\mathfrak {D}}_3$

**Table 46:**
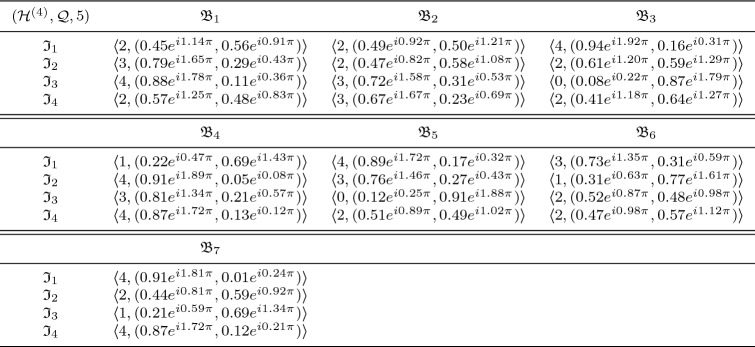
Tabulated form of $\hbox {CFFNS}_f$DM ${\mathfrak {G}}^{(4)}$ of expert ${\mathfrak {D}}_4$

**Table 47:**
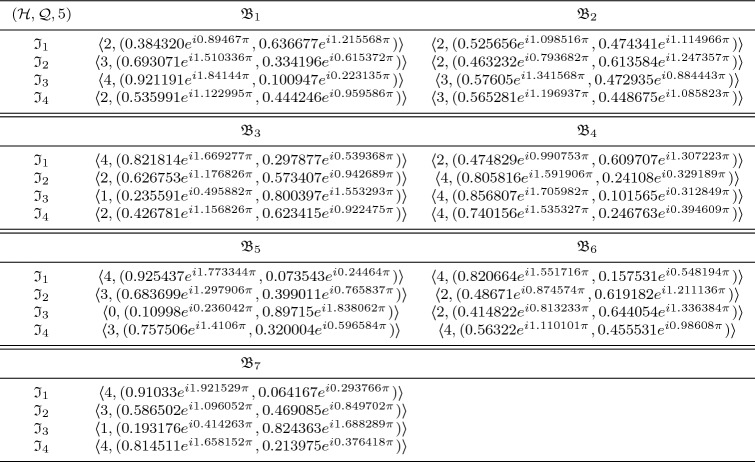
Tabulated form of $\hbox {ACFFNS}_f$DM ${\mathfrak {G}}$

- **Step 2.** The individual opinions of all decision-makers are assembled by employing the $\hbox {CFFNS}_f$WA operator, as defined in Equation 2 and the results are accumulated in the $\hbox {ACFFNS}_f$DM as shown in Table 47.
- **Step 3.** Experts associate $\hbox {CFFNS}_f$N to each attribute indicating the importance of that attribute in MAGDM problems which are summarized in Table 48. The $\hbox {CFFNS}_f$ weight of each attribute is accumulated by $\hbox {CFFNS}_f$WA operator defined in Equation 3 to form a $\hbox {CFFNS}_f$ weight vector $\kappa ,$ given by:
  10 $$\begin{aligned} \kappa =\left( \begin{array}{c} 3,(0.731811e^{i1.527149\pi },0.304928e^{i0.582725\pi }) \\ 4,(0.945072e^{i1.861182\pi },0.054923e^{i0.236347\pi }) \\ 4,(0.860568e^{i1.651356\pi },0.189314e^{i0.416769\pi }) \\ 3,(0.545443e^{i1.019597\pi },0.613887e^{i1.185280\pi }) \\ 4,(0.826558e^{i1.645141\pi },0.185730e^{i0.402961\pi }) \\ 3,(0.739877e^{i1.434762\pi },0.312240e^{i0.694595\pi }) \\ 3,(0.670899e^{i1.289629\pi },0.423652e^{i0.802981\pi }) \\ \end{array} \right) \end{aligned}$$

**Table 48:**
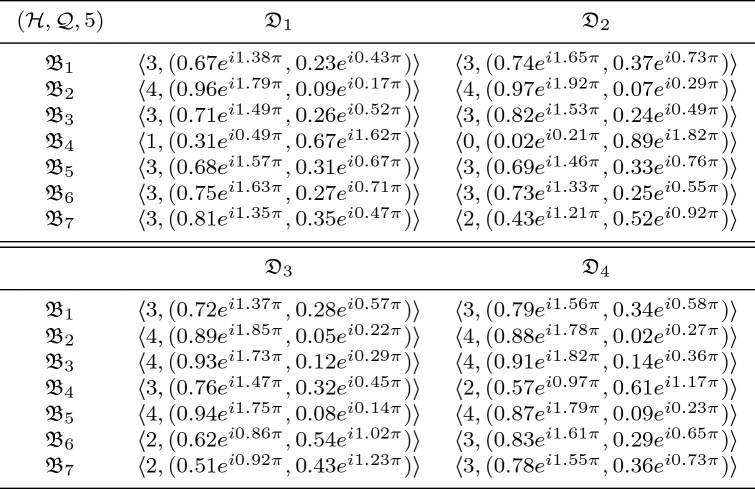
Importance weights of each attributes

- **Step 4.** The entries of $\hbox {AWCFFNS}_f$DM $\widehat{{\mathfrak {G}}}$ are obtained by Equation 4 by utilizing $\hbox {ACFFNS}_f$DM, given by Table 47, and the weight vector $\kappa$ of attributes in Equation 10. These entries are tabulated, as shown in Table 49.

**Table 49:**
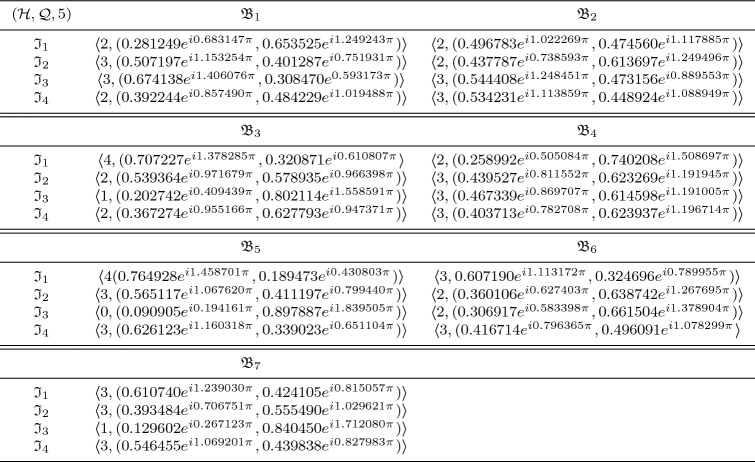
Tabular form of $\hbox {AWCFFNS}_f$DM $\widehat{{\mathfrak {G}}}$

- **Step 5.** In the proposed MAGDM problem, the attributes topography, soil and water quality & availability are benefit-type attributes whereas initial cost, environmental destruction, climate of the area and maintenance cost are cost-type attributes. $\hbox {CFFNS}_f$-PIS and $\hbox {CFFNS}_f$-NIS relative to each attribute, opted by Equations 5 and 6 , are arranged in Table 50.

**Table 50:** $\hbox {CFFNS}_f$-PIS and $\hbox {CFFNS}_f$-NIS

Attributes	$\hbox {CFFNS}_f$-PIS ($\widetilde{{\mathfrak {G}}}_t$)	$\hbox {CFFNS}_f$-NIS ($\breve{{\mathfrak {G}}}_t$)
${\mathfrak {B}}_1$	$\langle 2,(0.281249e^{i0.683147\pi },0.653525e^{i1.249243\pi })\rangle$	$\langle 3,(0.674138e^{i1.406076\pi },0.308470e^{i 0.593173\pi })\rangle$
${\mathfrak {B}}_2$	$\langle 2,(0.437787e^{i0.738593\pi },0.613697e^{i1.249496\pi })\rangle$	$\langle 3,(0.544408e^{i1.248451\pi },0.473156e^{i 0.889553\pi } )\rangle$
${\mathfrak {B}}_3$	$\langle 4,(0.707227e^{i1.378285\pi },0.320871e^{i0.610807\pi })\rangle$	$\langle 1,(0.202742e^{i0.409439\pi },0.802114e^{i 1.558591\pi })\rangle$
${\mathfrak {B}}_4$	$\langle 2,(0.258992e^{i0.505084\pi },0.740208e^{i1.508697\pi })\rangle$	$\langle 3,(0.467339e^{i0.869707\pi },0.614598e^{i 1.191005})\rangle$
${\mathfrak {B}}_5$	$\langle 0,(0.090905e^{i0.194161\pi },0.897887e^{i1.839505\pi })\rangle$	$\langle 4,(0.764928e^{i1.458701\pi },0.189473e^{i 0.430803\pi })\rangle$
${\mathfrak {B}}_6$	$\langle 3,(0.607190e^{i1.113172\pi },0.324696e^{i0.789955\pi })\rangle$	$\langle 2,(0.360106e^{i0.627403\pi },0.638742e^{i 1.267695\pi })\rangle$
${\mathfrak {B}}_7$	$\langle 3,(0.610740e^{i1.239030\pi },0.424105e^{i0.815057\pi })\rangle$	$\langle 1,(0.129602e^{i0.267123\pi },0.840450e^{i 1.712080\pi })\rangle$

- **Step 6.** Distance of each alternative from $\hbox {CFFNS}_f$-PIS and $\hbox {CFFNS}_f$-NIS is calculated by employing Equations 7 and 8 , respectively. These distance measures are tabulated in Table 51.

**Table 51:** Distance of each alternative from ideal solution

Alternatives	$d({\mathfrak {I}}_g, \widehat{{\mathfrak {G}}}_t)$	$d({\mathfrak {I}}_g, \breve{{\mathfrak {G}}}_t)$
${\mathfrak {I}}_1$	1.002077	2.187073
${\mathfrak {I}}_2$	1.955608	1.434256
${\mathfrak {I}}_3$	2.274187	0.950996
${\mathfrak {I}}_4$	1.851251	1.589221

- **Step 7.** Table 52 represents the revised closeness index corresponding to each alternative, evaluated by using Equation 9.

**Table 52:** Revised closeness index of each alternative

Alternatives	$\varPsi ({\mathfrak {I}}_g)$
${\mathfrak {I}}_1$	0
${\mathfrak {I}}_2$	$-1.29577$
${\mathfrak {I}}_3$	$-1.83465$
${\mathfrak {I}}_4$	$-1.12077$

- **Step 8.** The ranking of cities on the basis of revised closeness index is shown by Table 53. Since ${\mathfrak {I}}_1$ has maximum index value. Hence the experts will give suggestions to the investor to select Boston, Massachusetts for farming.

**Table 53:** Ranking of each alternative

Alternatives	${\mathfrak {I}}_1$	${\mathfrak {I}}_2$	${\mathfrak {I}}_3$	${\mathfrak {I}}_4$
Ranking	4	2	1	3

## Comparative analysis of $\hbox {CFFNS}_f$-TOPSIS technique

In this section, we solve the MAGDM problem “Selection of the most suitable city in the USA for farming” by Fermatean fuzzy TOPSIS (FF-TOPSIS) method, proposed by Senapati and Yager (2020), to authenticate the importance and validity of proposed model. The step wise solution of MAGDM problem following the Fermatean fuzzy TOPSIS method is given as follows:

- **Step 1.** The linguistic terms along with grades are same as given in Table 42. Since the existing technique only deals with multi-attribute decision-making (MADM) problems. Hence, the aggregated opinion of all experts, given in Table 47 is used by the investor but the grading part is excluded and CFFNs have taken to be zero to apply FF-TOPSIS method. Fermatean fuzzy decision matrix (FFDM) is arranged in Table 54. Moreover, to determine the role of each criteria, the decision-maker sets the weights of attributes as follows:
  $$\begin{aligned} \lambda =(0.15~~0.2~~0.17~~0.1~~0.16~~0.13~~0.09)^T \end{aligned}$$

**Table 54:**
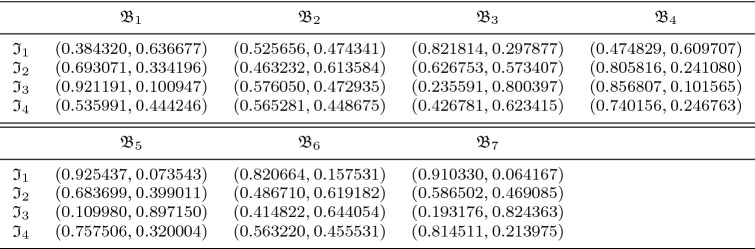
Tabular representation of FFDM $\mathbf {\vec{\mathfrak {G}}}$

- **Step 2.** The score of all FF numbers (FFNs) are determined to identify the Fermatean fuzzy positive and negative ideal solutions. The score of a FFN can be calculated by the following formula (Senapati and Yager 2020):
  11 $$\begin{aligned} S_c(\mathbf {{\mathfrak {G}}}_{a_{gt}})=s_{a_{gt}}^3-k_{a_{gt}}^3. \end{aligned}$$
  The score values of all entries of FFDM are assembled in Table 55. Table 56 represents the FF-PIS and FF-NIS relative to each attribute.

**Table 55:** Score values of FFNs to opt ideal solutions

	${\mathfrak {B}}_{1}$	${\mathfrak {B}}_{2}$	${\mathfrak {B}}_{3}$	${\mathfrak {B}}_4$	${\mathfrak {B}}_5$	${\mathfrak {B}}_6$	${\mathfrak {B}}_7$
${\mathfrak {I}}_{1}$	$-0.20132$	0.038520	0.528605	$-0.11960$	0.792178	0.548798	0.754126
${\mathfrak {I}}_{2}$	0.295590	$-0.131603$	0.057666	0.509237	0.256065	$-0.12209$	0.098530
${\mathfrak {I}}_{3}$	0.780688	0.085372	$-0.49969$	0.627949	$-0.72077$	$-0.19578$	-0.55301
${\mathfrak {I}}_{4}$	0.066309	0.090309	$-0.16455$	0.390455	0.401899	0.084136	0.530573

**Table 56:** FF-PIS and FF-NIS

Attributes	FF-PIS (${{\mathfrak {G}}}^+$)	$\hbox {B}_p\hbox {FNS}_f$-NIS (${{\mathfrak {G}}}^-$)
${\mathfrak {B}}_1$	(0.384320, 0.636677)	(0.921191, 0.100947)
${\mathfrak {B}}_2$	(0.463232, 0.613584)	(0.565281, 0.448675)
${\mathfrak {B}}_3$	(0.821814, 0.297877)	(0.235591, 0.800397)
${\mathfrak {B}}_4$	(0.474829, 0.609707)	(0.856807, 0.101565)
${\mathfrak {B}}_5$	(0.109980, 0.897150)	(0.925437, 0.073543)
${\mathfrak {B}}_6$	(0.820664, 0.157531)	(0.414822, 0.644054)
${\mathfrak {B}}_7$	(0.910330, 0.064167)	(0.193176, 0.824363)

- **Step 3.** Distance of each alternative ${\mathfrak {I}}_g$ from FF-PIS ${\mathfrak {B}}^+$ and FF-NIS ${\mathfrak {B}}^-$ is computed by employing the equations as follows (Senapati and Yager 2020):

$$\begin{aligned}&d({\mathfrak {I}}_g,{\mathfrak {G}}^+) = \frac{1}{2} \sum \limits _{t=1}^m \lambda _t \sqrt{\frac{1}{2} \bigg \{(\mathbf {s}_{a_{gt}}^3-({s}_{a_t}^+)^3)^2+ (\mathbf {k}_{a_{gt}}^3-({k}_{a_{t}}^+)^3)^2+((\mathbf {\pi }_{a_{gt}}^3 -({\pi }_{a_t}^+)^3)^2)\bigg \}}\\&d({\mathfrak {I}}_g,{\mathfrak {G}}^-) = \frac{1}{2} \sum \limits _{t=1}^m \lambda _t \sqrt{\frac{1}{2} \bigg \{(\mathbf {s}_{a_{gt}}^3-({s}_{a_t}^-)^3)^2+(\mathbf {k}_{a_{gt}}^3 -({k}_{a_{t}}^-)^3)^2+((\mathbf {\pi }_{a_{gt}}^3-({\pi }_{a_t}^-)^3)^2)\bigg \}} \end{aligned}$$

The results are tabulated in Table 57.

**Table 57:** Distance of each alternative from ideal solution

Alternatives	$d({\mathfrak {I}}_g, {{\mathfrak {G}}}^+)$	$d({\mathfrak {I}}_g, {{\mathfrak {G}}}^-)$
${\mathfrak {I}}_1$	0.071595	0.174813
${\mathfrak {I}}_2$	0.152928	0.130212
${\mathfrak {I}}_3$	0.182976	0.062972
${\mathfrak {I}}_4$	0.151493	0.137461

- **Step 4.** To find out the most suitable alternative, Table 58 represents the closeness index corresponding to each alternative which is evaluated by utilizing the Equation 9.

**Table 58:** Revised closeness index of each alternative

Alternatives	$\psi ({\mathfrak {I}}_g)$
${\mathfrak {I}}_1$	0
${\mathfrak {I}}_2$	$-1.39116$
${\mathfrak {I}}_3$	$-2.19548$
${\mathfrak {I}}_4$	$-1.32965$

- **Step 5.** The increasing order ranking of cities is shown by Table 59, where 1 is for minimum closeness index value and 4 is for highest index value. Since ${\mathfrak {I}}_1$ has the maximum index value. Hence the investor will select Boston, Massachusetts for farming.

**Table 59:** Ranking of each alternative

Alternatives	${\mathfrak {I}}_1$	${\mathfrak {I}}_2$	${\mathfrak {I}}_3$	${\mathfrak {I}}_4$
Ranking	4	2	1	3

### Results

**Fig. 3:**
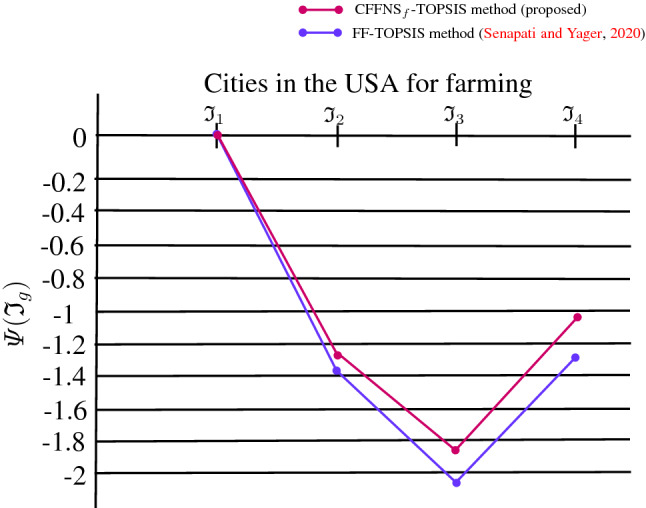
Comparative analysis

1. Now, we present a comparison of the proposed technique with the existing FF-TOPSIS method (Senapati and Yager 2020) to assess the accuracy of $\hbox {CFFNS}_f$-TOPSIS method. Despite the difference in revised closeness index calculated by both techniques, the final ranking of cities is the same. Thus, the same city is proclaimed as the most suitable one for farming in both methods. The results of the proposed and existing methods, including the final ranking and best alternative, are summarized in Table 60 as follows:
2. A comparison chart is designed in Figure 3 to envision the conformity of final results of compared and proposed MAGDM approaches which shows the effectuality and accountability of our proposed technique.
3. It is clear from the figure that both techniques elucidate the same outcome and ranking order that indicates the feasibility and sustain-ability of the presented technique.
4. Our proposed $\hbox {CFFNS}_f$-TOPSIS technique has capability to handle the vagueness and periodicity involve in the data simultaneously, but the compared FF-TOPSIS technique is limited to capture the ambiguity of non-periodic data that may cause to the inconsistency and specious outcomes. This extraordinary trait of the proposed strategy depicts that it is the more effective and generalized MAGDM strategy.
5. Due to the inadequacy of multi-valued grades and periodic terms, FF-TOPSIS cannot deal with $\hbox {CFFNS}_f$ information. On the other hand, $\hbox {CFFNS}_f$-TOPSIS method has potential to handle the FF information by taking phase terms equal to zero and neglecting the grades. Since the results are same in both cases which depicts the proposed method more adaptable than existing methods.

**Table 60:** Comparative analysis

Methods	Ranking of the most suitable city for farming	Best city
$\hbox {CFFNS}_f$-TOPSIS method (proposed)	${\mathfrak {I}}_3 \prec {\mathfrak {I}}_2 \prec {\mathfrak {I}}_4 \prec {\mathfrak {I}}_1$	${\mathfrak {I}}_1$
FF-TOPSIS method (Senapati and Yager 2020)	${\mathfrak {I}}_3 \prec {\mathfrak {I}}_2 \prec {\mathfrak {I}}_4 \prec {\mathfrak {I}}_1$	${\mathfrak {I}}_1$

## Merits of $\hbox {CFFNS}_f$S model and $\hbox {CFFNS}_f$-TOPSIS approach

1. In the modern era, the performance appraisal system is commonly used for the rating of restaurant management, schools, candidates for job, online services, online applications, products and websites, etc. The proposed model is designed to handle the rating-based assessment framework along with imprecise and vague two-dimensional information.
2. In this article, the robust technique of $\hbox {CFFNS}_f$-TOPSIS is developed for determining the best solution obtaining the closest distance from PIS and far away from NIS. The framework of the presented MAGDM strategy has remarkable aspects: it merges the fascinating advantages of TOPSIS with the hybrid model of $\hbox {CFFNS}_f$S. The advantage of the hybrid model is that it has the potency to handle vagueness and periodicity of parameterized graded information simultaneously.
3. The proposed model shows the same accuracy when applied to the existing models inclusive of FF, CIF, CPF, $\hbox {FFS}_f$, $\hbox {CIFS}_f$, $\hbox {CPFS}_f$, $\hbox {FFNS}_f$, $\hbox {CIFNS}_f$, and $\hbox {CPFNS}_f$ by taking either $N=2$ or substituting phase terms equal to zero or by applying both strategies. Hence, the developed technique deprives a adaptable tool that skillfully and efficiently accomplishes its decision-making chores with preciseness under traditional as well as two-dimensional vague information along with finely-graded parameters.

## Conclusion

Decision-making methods play an important role in the real life of human beings. The process of choosing the best option among a set of possible options is present in all human activities. In this paper, a new theory has been developed that serves as a mathematical tool which deals with the two-dimensional vague information, and which is a generalization of the fuzzy *N*-soft set. We have advanced a model, $\hbox {CFFNS}_f$S, that assesses the uncertain and vague data which has complex membership and non-membership values, parameterized information, and ordinal ranking systems. To establish a comparison between two $\hbox {CFFNS}_f$Ns, we have developed score and accuracy functions in a $\hbox {CFFNS}_f$ environment. We have defined some basic operations for the $\hbox {CFFNS}_f$S model that include: complement (weak complement, $\hbox {CFFNS}_f$S complement, and weak $\hbox {CFFNS}_f$S complement), union (extended union and restricted union), intersection (extended intersection and restricted intersection). We have also included relevant examples for these operations. In addition, we have presented algebraic and Yager operations for $\hbox {CFFNS}_f$Ns.

Moreover, we have accomplished three algorithms to resolve multi-attribute decision-making problems. These algorithms have been validated by two real-life examples related to the selection of cars and the selection of the best telecommunication company in Pakistan.

Furthermore, in order to analyze the validity, feasibility, and reliability of the proposed model, we have conducted a comparative study of our approach with two operators: the $\hbox {FFY}_w$G operator and the $\hbox {FFY}_w$A operator.

With respect to the proposed $\hbox {CFFNS}_f$-TOPSIS method, our method possesses the MAGDM potential of TOPSIS along with the adequacy of the proposed $\hbox {CFFNS}_f$ model to improve the exactness of decision-making results. The proposed method’s primary dominance is due to its capability to tackle two-dimensional imprecise information along with level of attributes based on alternative with the help of *N*-soft grading values as well as complex membership and non-membership values. The basic principle of the $\hbox {CFFNS}_f$-TOPSIS method is to find out the best solution possessing the proximity distance from the ideal solutions.

In the presented approach, the primary information has been equipped by ordered grades and their corresponding $\hbox {CFFNS}_f$Ns. The individual opinions have been aggregated by employing $\hbox {CFFNS}_f$WA operator. Further, the $\hbox {AWCFFNS}_f$DM has been acquired by the multiplication of $\hbox {CFFNS}_f$ weight vector of criteria and $\hbox {CFFNS}_f$DM. After examining the $\hbox {CFFNS}_f$-PIS and $\hbox {CFFNS}_f$-NIS, distance of each alternative from ideal solutions have been computed. Further, the revised closeness index of each alternative has been calculated by evolving the discrepancy of these variables from the ideal solution. After the evaluated results of the closeness index, the alternatives are arranged in an ascending order. The alternative having maximum value of closeness index will be the optimal solution of the MAGDM problem. The proposed approach has been endorsed by a numerical example related to the selection of the suitable city in the USA for farming.

Along with beneficial characteristics of the proposed technique based on TOPSIS method for MAGDM problems in two-dimensional data, it ensures the same level of authenticity under Fermatean fuzzy environment by eliminating the grades and substituting phase terms equal to zero. On the contrary, the adeptness of the FF-TOPSIS method is restricted to handle one dimensional phenomena, also it is unable to deal with MAGDM problems.

Moreover, the proposed $\hbox {CFFNS}_f$-TOPSIS method has an edge over the extant decision-making approaches as the $\hbox {CFFNS}_f$S model can effectively apply in the environments of FFS, CIFS, CPFS, $\hbox {FFS}_f$S, $\hbox {FNS}_f$, $\hbox {IFNS}_f$S, $\hbox {PFNS}_f$S, $\hbox {CPFNS}_f$ and so forth by taking either $N=2$ or substituting phase terms equal to zero or by applying both strategies.
